# Supplementary material for: Public awareness of and responses to media coverage of invitation errors in the Breast Screening Programme in England: a cross-sectional population survey
Source: BMJ Open. 2019 Sep 6;9(9):e028040. doi: 10.1136/bmjopen-2018-028040 (PMC6731953; doi:10.1136/bmjopen-2018-028040)
Supplement: Supplementary data [file bmjopen-2018-028040supp001.pdf]

APPENDIX 1 - SURVEY

[All Adults 16+ in England. Participants were shown the tablet screen and the following text was read out by interviewers]

Q.A In this part of the survey, I am going to ask you some questions related to health, including cancer, and recent news stories. These questions are asked on behalf of researchers from University College London. If you do not wish to answer a particular question during any part of this survey, you may refuse to answer and we will move to the next question. All your answers will be kept strictly confidential and you will be anonymous to the researchers.

The NHS currently offers breast cancer screening with mammography once every three years, to women aged between about 50 to 70 years in England.

Are you okay to continue with these questions?

1: Yes

2: No

As the questions can be perceived as sensitive, you can answer the questions on this machine yourself. I would now like to show you how to use the machine by going through a practice question with you.

[All Adults 16+ in England willing to continue. “Don’t know”/“Not sure”/“Prefer not to say” appeared at the top of the screen, out of view of participants, except for questions that participants completed themselves. Interviewers showed the screen to participants]

This is an example of a single-coded question

Q.B What is your favourite colour?

1: Red

2: Yellow

3: Blue

4: Green

Other colour (PEN -WRITE IN)

Don't know

Refused

[All females aged 16-69 in England willing to continue. Interviewers handed tablets to participants and stepped away from viewing the screen]

Q.1 Do you think you will go for breast screening when you are next offered it? REMEMBER TO TAP OK TO CONTINUE

1: Yes, definitely

2: Yes, probably

3: No, probably not

4: No, definitely not

Not sure

Prefer not to say

[All females aged 47+ in England willing to continue]

Q.2 Have you ever been invited for breast screening before? If you've only ever been offered a mammogram to investigate symptoms separately to the screening programme, please respond 'no'.

1: Yes

2: No

Not sure

Prefer not to say

[All females aged 47+ in England who have been invited for breast screening before]

Q.3 Have you ever been for breast screening as part of the screening programme?

1: Yes

2: No

Not sure

Prefer not to say

[All Adults 16+ in England willing to continue. This question allowed more than one response option. “None of the above”/”Prefer not to say” were mutually exclusive with other responses]

Q.4 Which of the following, if any, have you been diagnosed with? Please choose all that apply.

1: Bowel cancer

2: Lung cancer

3: Breast cancer

4: Cervical cancer

5: Prostate cancer

Other type of cancer - PEN WRITE IN

None of the above

Prefer not to say

[All Adults 16+ in England willing to continue. This question allowed more than one response option. “None of the above”/”Prefer not to say” were mutually exclusive with other responses]

Q.5 Has anyone you know ever been diagnosed with breast cancer? Please choose all that apply.

1: A close family member

2: Any other family member

3: A friend

4: A colleague

5: Any other person

Not sure

Prefer not to say

[All females aged 16+ in England willing to continue]

Q.6 How often do you worry about your chances of getting breast cancer yourself?

1: Never

2: Occasionally

3: Sometimes

4: Often

5: Very often

Not sure

Prefer not to say

Thank you for answering these questions - this is the end of this section for you.

[All Adults 16+ in England willing to continue. Participants handed the tablet back to the interviewer, who showed the screen and either read out or allowed participants to read subsequent questions]

Q.7 In May, it was reported that a computer algorithm failure had meant that a number of women did not receive invitations to their final routine breast cancer screening. The Health Secretary, Jeremy Hunt, said that women affected will be contacted by letter with an invitation for a catch-up screening test but some of the women who were not invited for their final appointment may have had their lives shortened.

Do you recall seeing or hearing anything about this news story before now?

1: Yes

2: No

Not sure

[All who recall seeing or hearing anything about this news story before now. This was a multiple choice question. The order of response options was randomised with “other websites” always following both “online news websites” and “social media websites”]

Q.8 Do you recall where you saw or heard this news story? Please choose all that apply.

1: Television

2: Print newspaper(s)

3: Radio

4: Online news websites

5: Social media websites

6: Other websites

7: Word of mouth

Other sources – PEN WRITE IN

Not sure

[All who recall seeing or hearing anything about this news story before now]

Q.9 Did you discuss or share the story with anyone else?

1: Yes

2: No

Not sure

[All who recall seeing or hearing anything about this news story before now. Participants were randomised to one of two orders of response options (1:1)]

Q.10 The Health Secretary, Jeremy Hunt, gave an estimate of the number of women who had failed to get invitations since 2009.

Which of the following do you think is the estimate that he gave?

1: 4,500 women

2: 45,000 women

3: 450,000 women

4: 4,500,000 women

Not sure

[All who recall seeing or hearing anything about this news story before now. Participants randomised to one of two orders of response options (1:1)]

Q.11 The Health Secretary also gave an estimate, based on computer modelling, of the number of women who may have had their lives shortened.

Which of the following do you think is the estimate that he gave?

1: Between 13 and 27 women

2: Between 135 and 270 women

3: Between 13 and 2,700 women

4: Between 1,350 and 2,700 women

Not sure

[All who gave an estimate in Q1 or Q11]

Q.12 How much did you trust these statistics when you heard them in the news?

1: Not at all

2: A little

3: Somewhat

4: A lot

Not sure

[All who do not trust the statistic]

Q.13 What were your reasons for not trusting these statistics when you heard them in the news?

PROBE: Any other reasons?

OPEN ENDED

[All who recall seeing or hearing anything about this news story before now]

Q.14 It was also reported that some health experts have said breast cancer screening can do "more harm than good" because they believe "breast screening…has no impact on all-cause death".

Do you recall seeing or hearing anything about this aspect of the news story before now?

1: Yes

2: No

Not sure

[All who recall seeing or hearing anything about this news story before now]

Q.15 The estimate of the number of women who may have had their lives shortened that the Health Secretary gave was between 135 and 270. It was also reported that one statistics expert has said this claim is "misleading" because they believe "there is only weak evidence that screening helps prolong life, particularly for older women" and that "contrary to popular belief, screening also does harm...for every 200 women attending screening between 50 and 70, we would expect one to have her early death from breast cancer prevented, but three to be unnecessarily treated for a harmless cancer that would not have troubled them".

Do you recall seeing or hearing anything about this aspect of the news story before now?

1: Yes

2: No

Not sure

[All Adults 16+ in England willing to continue]

Q.16 In general, how much do you trust your general practitioner?

1: Not at all

2: A little

3: Somewhat

4: A lot

Not sure

[All Adults 16+ in England willing to continue]

Q.17 In general, how much do you trust the NHS?

1: Not at all

2: A little

3: Somewhat

4: A lot

Not sure

[All Adults 16+ in England willing to continue]

Q.18 Routine screening means testing healthy people to find cancer before they have any symptoms.

Do you think routine cancer screening tests for healthy people are almost always a good idea?

1: Yes

2: No

Not sure

[Participants were handed an information card with the following text and asked to read it]

You may have some questions about breast cancer screening after this part of the survey. You can find out more by calling the NHS on a Freephone number (0800 169 2692) or via the web on https:\\www.nhs.uk\conditions\breast-cancer-screening\missed-invitations\.

[All Adults 16+ in England willing to continue]

Q.19 It was also reported in May that a volcano had erupted in Hawaii, leading to officials declaring a state of emergency and mandatory evacuation of 1,700 residents in the area.

Do you recall seeing or hearing anything about this news story before now?

1: Yes

2: No

Not sure

[All Adults 16+ in England willing to continue]

Q.20 The results of local elections held in England were also reported in May. The Labour Party won 2,350 seats, the Conservative Party won 1,332 seats, and the Liberal Democrats won 536 seats.

Do you recall seeing or hearing anything about this news story before now?

1: Yes

2: No

Not sure

[All Adults 16+ in England willing to continue]

Q.21 Please can I ask you what is the highest level of qualification you have received?

1: A. Graduate level qualifications and above: including higher degrees, professional qualifications at HE standard (e.g. chartered accountant, surveyor, Nursing, Teaching), NVQ and SVQ Level 4 or 5, Higher Education Diplomas, HNC and HND and BTEC Higher, RSA Higher Diploma

2: B. A-levels and AS levels and equivalents: including SCE Higher, Scottish Certificate 6th Year Studies, NVQ and SVQ and GSVQ level 3, GNVQ Advanced, ONC and OND and BTEC National, City and Guilds Advanced Craft, City and Guilds Final level or Part III, RSA Advanced Diploma

3: C. Trade apprenticeships

4: D. GCSEs and equivalents: including O level, SCE Standard, CSEs, NVQ and SVQ and GSVQ level 1 and 2, GNVQ and BTEC and SCOTVEC first, General diploma, City and Guilds Ordinary level, City and Guilds Ordinary level Part II, RSA State I-III or Diploma, SCOTVEC modules

5: E. Other qualifications (including overseas)

6: F. No formal qualifications

Don’t know

Refused
