# Supplementary material for: Public awareness of and responses to media coverage of invitation errors in the Breast Screening Programme in England: a cross-sectional population survey
Source: BMJ Open. 2019 Sep 6;9(9):e028040. doi: 10.1136/bmjopen-2018-028040 (PMC6731953; doi:10.1136/bmjopen-2018-028040)
Supplement: Supplementary data [file bmjopen-2018-028040supp002.pdf]

APPENDIX 2 – SUPPLMENTARY TABLES

Table A – Summary statistics describing the sample

Measure	Total* (n=1,894)	%	(95% CI)
**Awareness of the news about breast screening**			
Aware of the main story and both follow-up commentaries	250	13.2	11.7 to 14.8
Aware of the main story and overdiagnosis follow-up	188	9.9	8.6 to 11.3
Aware of the main story and all-cause mortality follow-up	117	6.2	5.2 to 7.3
Aware of the main story only	709	37.4	35.3 to 39.6
Unaware of the story	630	33.3	31.2 to 35.4
**Recruitment wave**			
Wave 2: 20-26th June	606	32.0	29.9 to 34.1
Wave 1: 6-10th June	1,288	68.0	65.9 to 70.1
**Gender**			
Male	801	42.3	40.1 to 44.5
Female	1,093	57.7	55.5 to 59.9
**Ethnicity**			
White British	1,555	82.4	80.7 to 84.1
Other groups	331	17.6	15.9 to 19.3
**Marital status**			
Married or living as a married	1,039	54.9	52.6 to 57.1
Widowed, divorced and separated	382	20.2	18.4 to 22.0
Single	473	25.0	23.1 to 27.0
**Highest level of education**			
Graduate level qualifications and above	530	28.2	26.2 to 30.3
A-levels and AS levels and equivalents	461	24.6	22.7 to 26.5
GCSEs and equivalents	459	24.5	22.5 to 26.4
Trade apprenticeships or other qualifications	93	5.0	4.0 to 6.0
No formal qualifications	334	17.8	16.1 to 19.6
**Social grade**			
Grade A or B	336	17.7	16.1 to 19.5
Grade C1	539	28.5	26.5 to 30.5
Grade C2	423	22.3	20.5 to 24.3
Grade D or E	596	31.5	29.4 to 33.6
**Employment status**			
Working	862	45.5	43.3 to 47.8
Not working	1,032	54.5	52.2 to 56.7
**Area type**			
Urban	1,542	81.4	79.6 to 83.1
Rural	352	18.6	16.9 to 20.4
**Personal diagnosis of cancer**			
Yes	156	8.4	7.2 to 9.7
No	1,705	91.6	90.3 to 92.8
**Personal experience of breast screening**			
Taken part	441	23.7	21.8 to 25.7
Invited, never taken part	56	3.0	2.3 to 3.9
Not eligible or not invited	1,364	73.3	71.2 to 75.3
**Belief that screening is almost always a good idea**			
Yes	1,737	91.7	90.4 to 92.9
No or not sure	157	8.3	7.1 to 9.6
**Awareness of the news about the volcanic eruption**			
Yes	1,435	75.8	73.8 to 77.7
No or not sure	459	24.2	22.3 to 26.2
**Awareness of the news about the local elections**			
Yes	1,198	63.3	61.1 to 65.4
No or not sure	696	36.7	34.6 to 38.9
**General level of trust in participants' GPs**			
A lot	1,009	55.2	52.9 to 57.5
Somewhat	540	29.6	27.5 to 31.7
A little	219	12.0	10.6 to 13.5
Not at all	59	3.2	2.5 to 4.1
**General level of trust in the NHS**			

Ghanouni A, *et al. BMJ Open* 2019; 9:e028040. doi: 10.1136/bmjopen-2018-028040

A lot	1,016	54.2	54.9 to 59.5
Somewhat	619	33.0	32.7 to 37.1
A little	184	9.8	9.0 to 11.8
Not at all	56	3.0	2.4 to 4.0
**Frequency of worry about breast cancer**			
Very often	46	4.6	3.4 to 6.0
Often	55	5.4	4.2 to 7.0
Sometimes	216	21.4	18.9 to 24.0
Occasionally	302	29.9	27.1 to 32.8
Never	391	38.7	35.7 to 41.7
**Breast screening intentions for next invitation**			
Yes, definitely	690	84.7	82.1 to 87.0
Yes, probably	88	10.8	8.8 to 13.1
No, probably not	18	2.2	1.4 to 3.4
No, definitely not	19	2.3	1.5 to 3.5

Table B – Full results of the binary logistic regression model testing for variables associated with whether participants responded to questions on the survey module

		**Responded vs. Did not respond to the survey questions: n (%)**		**Adjusted OR, 95% CI**	**p-value**
**Characteristic**	**Total (n=2,665)**	**Did not respond (n=779; 29.2%)**	**Responded (n=1,886; 70.8%)**	**Responded to questions (vs. Did not respond)**	
**Recruitment wave**					
Wave 2: 20-26th June	908	303 (33.4)	605 (66.6)	0.73, 0.61 to 0.87	**<.0005***
vs. Wave 1: 6-10th June	1,757	476 (27.1)	1,281 (72.9)		
**Gender**					
Male	1,270	474 (37.3)	796 (62.7)	0.46, 0.39 to 0.55	**<.0005**
vs. Female	1,395	305 (21.9)	1,090 (78.1)		
**Ethnicity**					
White British	2,139	584 (27.3)	1,555 (72.7)	1.69, 1.37 to 2.10	**<.0005**
vs. Other groups	526	195 (37.1)	331 (62.9)		
**Marital status**					Overall: **.001**
Married/Living as a couple	1,441	407 (28.2)	1,034 (71.8)	1.48, 1.18 to 1.85	**.001**
Widowed/Divorced/Separated	517	135 (26.1)	382 (73.9)	1.65, 1.21 to 2.24	**.002**
vs. Single	707	237 (33.5)	470 (66.5)		
**Social grade**					Overall: **.003**
Grade A or B	450	115 (25.6)	335 (74.4)	1.54, 1.18 to 2.02	**.002**
Grade C1	726	190 (26.2)	536 (73.8)	1.44, 1.15 to 1.81	**.002**
Grade C2	596	174 (29.2)	422 (70.8)	1.28, 1.01 to 1.63	**.045**
vs. Grade D or E	893	300 (33.6)	593 (66.4)		
**Employment status**					
Working	1,225	366 (29.9)	859 (70.1)	0.79, 0.65 to 0.97	**.026**
vs. Not working	1,440	413 (28.7)	1,027 71.3)		
**Area type**					
Urban	2,164	629 (29.1)	1,535 (70.9)	1.14, 0.91 to 1.42	.246
vs. Rural	501	150 (29.9)	351 (70.1)		
**Age (in years)**	2,665	52.1 (21.0)	50.8 (20.5)	0.99, 0.98 to 1.00	**<.0005**

*Bold denotes p<.05

Ghanouni A, *et al. BMJ Open* 2019; 9:e028040. doi: 10.1136/bmjopen-2018-028040

Table C – Sources of news about the breast screening story

**Source of information**	**Total (n=1,264)**	**%**	**(95% CI)**
Television	971	76.8	74.4 to 79.1
Radio	271	21.4	19.2 to 23.8
Print newspaper(s)	169	13.4	11.6 to 15.3
Online news websites	134	10.6	9.0 to 12.4
Social media websites	68	5.4	4.2 to 6.7
Other websites	11	0.9	0.5 to 1.5
Word of mouth	43	3.4	2.5 to 4.5
Other sources	8	0.6	0.3 to 1.2
Discussed or shared the story with someone else	450	35.6	33.0 to 38.3

Ghanouni A, *et al. BMJ Open* 2019; 9:e028040. doi: 10.1136/bmjopen-2018-028040

Table D – Full results of the binary logistic regression model testing for an association between awareness of the breast screening media coverage and trust in participants' GPs

		A lot vs. Not at all; a little; somewhat: n (%)		Adjusted OR, 95% CI	p-value
**Characteristic**	**Total (n=1,746)**	**Less than a lot (n=781; 44.7%)**	**A lot (n=965; 55.3%)**	**A lot (vs. Less than a lot)**	
**Screening story awareness**					Overall: .729
Aware of the main story and both follow-up commentaries	238	98 (41.2)	140 (58.8)	1.10, 0.74 to 1.64	.653
Aware of the main story and overdiagnosis follow-up	172	66 (38.4)	106 (61.6)	1.31, 0.85 to 2.03	.218
Aware of the main story and all-cause mortality follow-up	107	49 (45.8)	58 (54.2)	1.21, 0.73 to 2.02	.459
Aware of the main story only	655	280 (42.7)	375 (57.3)	1.17, 0.88 to 1.57	.283
vs. Unaware of the story	574	288 (50.2)	286 (49.8)		
**Recruitment wave**					
Wave 2: 20-26th June	557	255 (45.8)	663 (55.8)	0.81, 0.64 to 1.04	.097
vs. Wave 1: 6-10th June	1,189	526 (44.2)	302 (54.2)		
**Gender**					
Male	754	317 (42.0)	437 (58.0)	1.15, 0.86 to 1.54	.334
vs. Female	992	464 (46.8)	528 (53.2)		
**Ethnicity**					
White British	1,450	614 (42.3)	836 (57.7)	1.17, 0.85 to 1.61	.328
vs. Other groups	296	167 (54.4)	129 (43.6)		
**Marital status**					Overall: .504
Married/Living as a couple	964	422 (42.8)	542 (56.2)	1.04, 0.77 to 1.42	.782
Widowed/Divorced/Separated	341	139 (40.8)	202 (59.2)	0.86, 0.57 to 1.29	.460
vs. Single	441	220 (49.9)	221 (50.1)		
**Highest level of education**					Overall: .056
Graduate level/Above	494	230 (46.6)	264 (53.4)	0.64, 0.42 to 0.98	.042
A-levels/AS levels/Equivalents	438	201 (45.9)	237 (54.1)	0.70, 0.46 to 1.06	.089
GCSEs/Equivalents	429	213 (49.7)	216 (50.3)	0.63, 0.43 to 0.94	.022
Trade apprenticeships/Other	86	41 (47.7)	45 (52.3)	0.44, 0.25 to 0.80	.007
vs. No formal qualifications	299	96 (32.1)	203 (67.9)		
**Social grade**					Overall: .711
Grade A or B	317	128 (40.4)	189 (59.6)	1.20, 0.82 to 1.76	.342
Grade C1	505	231 (45.7)	274 (54.3)	1.02, 0.74 to 1.39	.923
Grade C2	385	181 (47.0)	204 (53.0)	0.97, 0.70 to 1.36	.874
vs. Grade D or E	539	241 (44.7)	298 (55.3)		
**Employment status**					
Working	806	411 (51.0)	395 (49.0)	0.82, 0.63 to 1.07	.135
vs. Not working	940	370 (39.4)	570 (60.6)		
**Area type**					
Urban	1,420	635 (44.7)	785 (55.3)	1.12, 0.84 to 1.50	.430
vs. Rural	326	146 (44.8)	180 (55.2)		
**Personal diagnosis of cancer**					
Yes	1,599	718 (44.9)	881 (55.1)	0.84, 055 to 1.28	.404
vs. No	147	63 (42.9)	84 (57.1)		
**Personal experience of breast screening**					Overall: .284
Taken part	411	159 (38.7)	252 (61.3)	1.33, 0.91 to 1.95	.145
Invited, never taken part	48	21 (43.8)	27 (56.3)	0.95, 0.46 to 1.98	.894
vs. Not eligible or not invited	1,287	601 (46.7)	686 (53.3)		
**Belief that screening is almost always a good idea**					
Yes	1,609	701 (43.6)	908 (56.4)	1.30, 0.85 to 1.97	.230
vs. No or not sure	137	80 (58.4)	57 (41.6)		
**Awareness of volcano news**					
Yes	1,332	565 (42.4)	767 (57.6)	1.04, 0.77 to 1.40	.789
vs. No or not sure	414	216 (52.2)	198 (47.8)		

4

**Awareness of election news**					
Yes	1,114	467 (41.9)	647 (58.1)	1.19, 0.93 to 1.54	.172
vs. No or not sure	632	314 (49.7)	318 (50.3)		
**General level of trust in the NHS**					Overall: **<.0005***
A lot	943	202 (21.4	741 (78.6)	13.53, 6.65 to 27.54	**<.0005**
Somewhat	589	409 (69.4)	180 (30.6)	1.62, 0.80 to 3.31	.183
A little	163	130 (79.8)	33 (20.2)	1.00, 0.46 to 2.21	.994
vs. Not at all	51	40 (78.4)	11 (21.6)		
**Age (in years)**	1,746	46.9 (19.1)	53.9 (21.0)	1.01, 1.00 to 1.02	.087

*Bold denotes p<.05

Ghanouni A, *et al. BMJ Open* 2019; 9:e028040. doi: 10.1136/bmjopen-2018-028040

Table E – Full results of the binary logistic regression model testing for an association between awareness of the breast screening media coverage and trust in the NHS

		A lot vs. Not at all; a little; somewhat: n (%)		Adjusted OR, 95% CI	p-value
**Characteristic**	**Total (n=1,746)**	**Less than a lot (n=803; 46.0%)**	**A lot (n=943; 54.0%)**	**A lot (vs. Less than a lot)**	
**Screening story awareness**					Overall: .290
Aware of the main story and both follow-up commentaries	238	102 (42.9)	136 (57.1)	0.87, 0.59 to 1.30	.503
Aware of the main story and overdiagnosis follow-up	172	76 (44.2)	96 (55.8)	0.78, 0.51 to 1.21	.267
Aware of the main story and all-cause mortality follow-up	107	57 (53.3)	50 (46.7)	0.58, 0.35 to 0.97	.039
Aware of the main story only	655	299 (45.6)	356 (54.4)	0.81, 0.60 to 1.09	.160
vs. Unaware of the story	574	269 (46.9)	305 (53.1)		
**Recruitment wave**					
Wave 2: 20-26th June	557	248 (44.5)	309 (55.5)	1.21, 0.95 to 1.55	.118
vs. Wave 1: 6-10th June	1,189	555 (46.7)	634 (53.3)		
**Age**					Overall: .052
65+	530	198 (37.4)	332 (62.6)	1.04, 0.61 to 1.79	.880
55-64	245	111 (45.3)	134 (54.7)	0.76, 0.44 to 1.30	.309
45-54	235	115 (48.9)	120 (51.1)	0.83, 0.50 to 1.39	.484
35-44	245	135 (55.1)	110 (44.9)	0.59, 0.36 to 0.96	.035
25-34	265	152 (57.4)	113 (42.6)	0.56, 0.35 to 0.91	.018
vs. 16-24	226	92 (40.7)	134 (59.3)		
**Gender**					
Male	754	319 (42.3)	435 (57.7)	0.99, 0.73 to 1.33	.985
vs. Female	992	484 (48.8)	508 (51.2)		
**Ethnicity**					
White British	1,450	634 (43.7)	816 (56.3)	1.47, 1.07 to 2.02	**.019***
vs. Other groups	296	169 (57.1)	127 (42.9)		
**Marital status**					Overall: .870
Married/Living as a couple	964	440 (45.6)	524 (54.4)	1.07, 0.78 to 1.47	.685
Widowed/Divorced/Separated	341	151 (44.3)	190 (55.7)	1.00, 0.66 to 1.50	.990
vs. Single	441	212 (48.1)	229 (51.9)		
**Highest level of education**					Overall: .076
Graduate level/Above	494	233 (47.2)	261 (52.8)	1.20, 0.79 to 1.83	.386
A-levels/AS levels/Equivalents	438	199 (45.4)	239 (54.6)	1.12, 0.75 to 1.67	.582
GCSEs/Equivalents	429	224 (52.2)	205 (47.8)	0.86, 0.59 to 1.26	.447
Trade apprenticeships/Other	86	32 (37.2)	54 (62.8)	1.85, 1.01 to 3.39	.047
vs. No formal qualifications	299	115 (38.5)	184 (61.5)		
**Social grade**					Overall: .990
Grade A or B	317	140 (44.2)	177 (55.8)	0.96, 0.65 to 1.41	.828
Grade C1	505	232 (45.9)	273 (54.1)	0.99, 0.72 to 1.37	.968
Grade C2	385	179 (46.5)	206 (53.5)	1.02, 0.73 to 1.43	.892
vs. Grade D or E	539	252 (46.8)	206 (53.5)		
**Employment status**					
Working	806	410 (50.9)	396 (49.1)	0.94, 0.71 to 1.25	.673
vs. Not working	940	393 (41.8)	547 (58.2)		
**Area type**					
Urban	1,420	656 (46.2)	764 (53.8)	0.96, 0.72 to 1.29	.795
vs. Rural	326	147 (45.1)	179 (54.9)		
**Personal diagnosis of cancer**					
Yes	1,599	737 (46.1)	862 (53.9)	1.00, 0.66 to 1.50	.994
vs. No	147	66 (44.9)	81 (55.1)		
**Personal experience of breast screening**					Overall: .062
Taken part	411	193 (47.0)	218 (53.0)	0.65, 0.43 to 0.97	.035
Invited, never taken part	48	20 (41.7)	28 (58.3)	1.10, 0.51 to 2.35	.813
vs. Not eligible or not invited	1,287	590 (45.8)	697 (54.2)		

Ghanouni A, *et al. BMJ Open* 2019; 9:e028040. doi: 10.1136/bmjopen-2018-028040

**Belief that screening is almost always a good idea**					
Yes	1,609	715 (44.4)	894 (55.6)	1.96, 1.28 to 3.00	**.002**
vs. No or not sure	137	88 (64.2)	49 (35.8)		
**Awareness of volcano news**					
Yes	1,332	594 (44.6)	738 (55.4)	1.00, 0.74 to 1.35	.987
vs. No or not sure	414	209 (50.5)	205 (49.5)		
**Awareness of election news**					
Yes	1,114	491 (44.1)	623 (55.9)	1.03, 0.80 1.33	.817
vs. No or not sure	632	312 (49.4)	320 (50.6)		
**General level of trust in participants' GPs**					Overall: **<.0005**
A lot	965	224 (23.2)	741 (76.8)	11.98, 6.07 to 23.64	**<.0005**
Somewhat	513	370 (72.1)	143 (27.9)	1.39, 0.70 to 2.76	.350
A little	212	165 (77.8)	47 (22.2)	1.12, 0.54 to 2.33	.770
vs. Not at all	56	44 (78.6)	12 (21.4)		

*Bold denotes p<.05

Ghanouni A, *et al. BMJ Open* 2019; 9:e028040. doi: 10.1136/bmjopen-2018-028040

Table F – Full results of the binary logistic regression model testing for an association between awareness of the breast screening media coverage and frequency of worry about breast cancer

		Never; occasionally vs. Sometimes; often; very often: n (%)/M (SD)		Adjusted OR, 95% CI	p-value
Characteristic	Total (n=700)	Never; occasionally (n=441; 63.0%)	Sometimes; often; very often (n=259; 37.0%)	Sometimes; often; very often (vs. Never; occasionally)	
**Screening story awareness**					Overall: .198
Aware of the main story and both follow-up commentaries	88	65 (73.9)	23 (26.1)	0.85, 0.46 to 1.58	.614
Aware of the main story and overdiagnosis follow-up	63	42 (66.7)	21 (33.3)	1.05, 0.55 to 2.01	.878
Aware of the main story and all-cause mortality follow-up	36	25 (69.4)	11 (30.6)	1.10, 0.49 to 2.49	.819
Aware of the main story only	270	153 (56.7)	117 (43.3)	1.49, 0.98 to 2.25	.062
vs. Unaware of the story	243	156 (64.2)	87 (35.8)		
**Recruitment wave**					
Wave 2: 20-26th June	229	152 (66.4)	77 (33.6)	0.83, 0.58 to 1.19	.304
vs. Wave 1: 6-10th June	471	289 (61.4)	182 (38.6)		
**Ethnicity**					
White British	563	354 (62.9)	209 (37.1)	0.90, 0.58 to 1.39	.635
vs. Other groups	137	87 (63.5)	50 (36.5)		
**Marital status**					Overall: .272
Married/Living as a couple	403	255 (63.3)	148 (36.7)	1.14, 0.76 to 1.72	.519
Widowed/Divorced/Separated	100	58 (58.0)	42 (42.0)	1.61, 0.90 to 2.87	.110
vs. Single	197	128 (65.0)	69 (35.0)		
**Highest level of education**					Overall: **.026***
Graduate level/Above	230	149 (64.8)	81 (35.2)	0.40, 0.21 to 0.79	**.008**
A-levels/AS levels/Equivalents	206	138 (67.0)	68 (33.0)	0.67, 0.19 to 0.70	**.002**
GCSEs/Equivalents	176	105 (59.7)	71 (40.3)	0.57, 0.31 to 1.04	.068
Trade apprenticeships/Other	17	12 (70.6)	5 (29.4)	0.33, 0.10 to 1.10	.072
vs. No formal qualifications	71	37 (52.1)	34 (47.9)		
**Social grade**					Overall: .704
Grade A or B	125	84 (67.2)	41 (32.8)	1.07, 0.60 to 1.90	.819
Grade C1	209	123 (58.9)	86 (41.1)	1.31, 0.80 to 2.13	.282
Grade C2	165	105 (63.6)	60 (36.4)	1.11, 0.67 to 1.83	.691
vs. Grade D or E	201	129 (64.2)	72 (35.8)		
**Employment status**					
Working	392	239 (61.0)	153 (39.0)	1.15, 0.81 to 1.64	.435
vs. Not working	308	202 (65.6)	106 (34.4)		
**Area type**					
Urban	574	366 (63.8)	208 (36.2)	0.83, 054 to 1.26	.378
vs. Rural	126	75 (59.5)	51 (40.5)		
**Personal diagnosis of cancer**					
Yes	44	26 (59.1)	18 (40.9)	1.62, 0.82 to 3.22	.169
vs. No	656	415 (63.3)	241 (36.7)		
**Personal experience of breast screening**					Overall: .428
Taken part	221	151 (68.3)	70 (31.7)	0.66, 0.36 to 1.23	.193
Invited, never taken part	34	23 (67.6)	11 (32.4)	0.75, 0.30 to 1.89	.537
vs. Not eligible or not invited	445	267 (60.0)	178 (40.0)		
**Belief that screening is almost always a good idea**					
Yes	660	412 (62.4)	248 (37.6)	1.12, 0.50 to 2.51	.779
vs. No or not sure	40	29 (72.5)	11 (27.5)		
**Awareness of volcano news**					
Yes	505	323 (64.0)	182 (36.0)	0.91, 0.61 to 1.36	.645
vs. No or not sure	195	118 (60.5)	77 (39.5)		
**Awareness of election news**					
Yes	424	275 (64.9)	149 (35.1)	0.84, 0.59 to 1.20	.340

Ghanouni A, *et al. BMJ Open* 2019; 9:e028040. doi: 10.1136/bmjopen-2018-028040

vs. No or not sure	276	166 (60.1)	110 (39.9)		
**General level of trust in participants' GPs**					Overall: .791
A lot	339	220 (64.9)	119 (35.1)	1.26, 0.50 to 3.15	.626
Somewhat	221	131 (59.3)	90 (40.7)	1.47, 0.58 to 3.72	.412
A little	109	69 (63.3)	40 (36.7)	1.25, 0.49 to 3.22	.641
vs. Not at all	31	21 (67.7)	10 (32.3)		
**General level of trust in the NHS**					Overall: .744
A lot	344	223 (64.8)	121 (35.2)	1.14, 0.41 to 3.21	.802
Somewhat	257	151 (58.8)	106 (41.2)	1.39, 0.49 to 3.91	.535
A little	76	51 (67.1)	25 (32.9)	1.13, 0.38 to 3.36	.833
vs. Not at all	23	16 (69.6)	7 (30.4)		
**Breast screening intentions for next invitation**					Overall: .163
Yes, definitely	601	369 (61.4)	232 (38.6)	1.48, 0.47 to 4.68	.503
Yes, probably	70	49 (70.0)	21 (30.0)	0.93, 0.27 to 3.25	.913
No, probably not	13	12 (92.3)	1 (7.7)	0.25, 0.02 to 2.70	.254
vs. No, definitely not	16	11 (68.8)	5 (31.3)		
**Age (in years)**	700	43.7 (15.5)	41.8 (14.3)	0.99, 0.97 to 1.01	.463

*Bold denotes p<.05

Ghanouni A, *et al. BMJ Open* 2019; 9:e028040. doi: 10.1136/bmjopen-2018-028040

Table G – Full results of the binary logistic regression model testing for an association between awareness of the breast screening media coverage and breast screening intentions

		Yes, definitely vs. Yes, probably; no, probably not; no, definitely not: n (%)		Adjusted OR, 95% CI	p-value
**Characteristic**	**Total (n=700)**	**No definite intention (n=99; 14.1%)**	**Definite intention (n=601; 85.9%)**	**Definite intention (vs. No definite intention)**	
**Screening story awareness**					Overall: .108
Aware of the main story and both follow-up commentaries	88	10 (11.4)	78 (88.6)	2.01, 0.74 to 5.48	.172
Aware of the main story and overdiagnosis follow-up	63	4 (4.3)	59 (93.7)	2.66, 0.79 to 8.89	.113
Aware of the main story and all-cause mortality follow-up	36	6 (16.7)	30 (83.3)	0.66, 0.20 to 2.13	.486
Aware of the main story only	270	22 (8.1)	248 (91.9)	1.88, 0.99 to 3.57	.054
vs. Unaware of the story	243	57 (23.5)	186 (76.5)		
**Recruitment wave**					
Wave 2: 20-26th June	229	40 (17.5)	189 (82.5)	0.71, 0.42 to 1.21	.211
vs. Wave 1: 6-10th June	471	59 (12.5)	412 (87.5)		
**Age**					Overall: .050
65+	69	7 (10.1)	62 (89.9)	0.84, 0.13 to 5.37	.855
55-64	123	5 (4.1)	118 (95.9)	2.49, 0.42 to 14.74	.313
45-54	128	10 (7.8)	118 (92.2)	3.59, 1.10 to 11.69	.034
35-44	143	21 (14.7)	122 (85.3)	2.72, 1.16 to 6.41	.022
25-34	144	29 (20.1)	115 (79.9)	2.82, 1.24 to 6.42	.014
vs. 16-24	93	27 (29.0)	66 (71.0)		
**Ethnicity**					
White British	563	72 (12.8)	491 (87.2)	0.96, 0.51 to 1.83	.905
vs. Other groups	137	27 (19.7)	110 (80.3)		
**Marital status**					Overall: .321
Married/Living as a couple	403	49 (12.2)	354 (87.8)	0.99, 0.53 to 1.84	.970
Widowed/Divorced/Separated	100	6 (6.0)	94 (94.0)	2.18, 0.73 to 6.53	.163
vs. Single	197	44 (22.3)	153 (77.7)		
**Highest level of education**					Overall: .169
Graduate level/Above	230	35 (15.2)	195 (84.8)	0.69, 0.22 to 2.20	.533
A-levels/AS levels/Equivalents	206	35 (17.0)	171 (83.0)	0.87, 0.29 to 2.62	.808
GCSEs/Equivalents	176	19 (10.8)	157 (89.2)	1.92, 0.62 to 5.92	.259
Trade apprenticeships/Other	17	3 (17.6)	14 (82.4)	1.38, 0.24 to 0.81	.720
vs. No formal qualifications	71	7 (9.9)	64 (90.1)		
**Social grade**					Overall: .186
Grade A or B	125	14 (11.2)	111 (88.8)	1.87, 0.76 to 4.61	.177
Grade C1	209	25 (12.0)	184 (88.0)	2.36, 1.08 to 5.16	.032
Grade C2	165	24 (14.5)	141 (85.5)	1.85, 0.86 to 3.98	.118
vs. Grade D or E	201	36 (17.9)	165 (82.1)		
**Employment status**					
Working	392	56 (14.3)	336 (85.7)	0.70, 0.39 to 1.26	.238
vs. Not working	308	43 (14.0)	265 (86.0)		
**Area type**					
Urban	574	83 (14.5)	491 (85.5)	0.79, 0.39 to 1.59	.512
vs. Rural	126	16 (12.7)	110 (87.3)		
**Personal diagnosis of cancer**					
Yes	44	7 (15.9)	37 (84.1)	0.94, 0.30 to 2.98	.918
vs. No	656	92 (14.0)	564 (86.0)		
**Personal experience of breast screening**					Overall: **.013***
Taken part	221	9 (4.1)	212 (95.9)	6.12, 1.37 to 27.33	**.018**
Invited, never taken part	34	6 (17.6)	28 (82.4)	0.99, 0.21 to 4.61	.986
vs. Not eligible or not invited	445	84 (18.9)	361 (81.1)		
**Belief that screening is almost always a good idea**					

Yes	660	81 (12.3)	579 (87.7)	9.08, 3.77 to 21.88	**<.0005**
vs. No or not sure	40	18 (45.0)	22 (55.0)		
**Awareness of volcano news**					
Yes	505	54 (10.7)	451 (89.3)	1.34, 0.74 to 2.41	.335
vs. No or not sure	195	45 (23.1)	150 (76.9)		
**Awareness of election news**					
Yes	424	47 (11.1)	377 (88.9)	1.42, 0.82 to 2.46	.208
vs. No or not sure	276	52 (18.8)	224 (81.2)		
**General level of trust in participants' GPs**					Overall: .025
A lot	339	31 (9.1)	308 (90.9)	1.55, 0.45 to 5.29	.487
Somewhat	221	44 (19.9)	177 (80.1)	0.76, 0.23 to 5.67	.663
A little	109	16 (14.7)	93 (85.3)	2.48, 0.69 to 8.90	.163
vs. Not at all	31	8 (25.8)	23 (74.2)		
**General level of trust in the NHS**					Overall: .007
A lot	344	34 (9.9)	310 (90.1)	1.16, 0.29 to 4.64	.832
Somewhat	257	42 (16.3)	215 (83.7)	0.70, 0.18 to 2.79	.614
A little	76	18 (23.7)	58 (76.3)	0.27, 0.06 to 1.11	.068
vs. Not at all	23	5 (21.7)	18 (78.3)		
**Frequency of worry about breast cancer**					Overall: **.028**
Very often	39	3 (7.7)	36 (92.3)	3.00, 0.72 to 12.51	.132
Often	43	5 (9.3)	39 (90.7)	2.95, 0.85 to 10.26	.089
Sometimes	177	20 (11.3)	157 (88.7)	2.59, 1.31 to 5.15	**.006**
Occasionally	231	27 (11.7)	204 (88.3)	2.15, 1.15 to 4.02	**.016**
vs. Never	210	45 (21.4)	165 (78.6)		

*Bold denotes p<.05

Table H – Number of participants with missing data for each variable of interest

**Measure**	**Total (n=1,894)**
Awareness of the news about breast screening	0
Recruitment wave	0
Gender	0
Ethnicity	8
Marital status	0
Highest level of education	17
Social grade	0
Employment status	0
Area type	0
Personal diagnosis of cancer	33
Personal experience of breast screening	33
Belief that screening is almost always a good idea	0
Awareness of the news about the volcanic eruption	0
Awareness of the news about the local elections	0
General level of trust in participants' GPs	67
General level of trust in the NHS	19
Frequency of worry about breast cancer	22
Breast screening intentions for next invitation	9

Ghanouni A, *et al. BMJ Open* 2019; 9:e028040. doi: 10.1136/bmjopen-2018-028040
