# Supplementary material for: Public awareness of and responses to media coverage of invitation errors in the Breast Screening Programme in England: a cross-sectional population survey
Source: BMJ Open. 2019 Sep 6;9(9):e028040. doi: 10.1136/bmjopen-2018-028040 (PMC6731953; doi:10.1136/bmjopen-2018-028040)
Supplement: Author's manuscript [file bmjopen-2018-028040.draft_revisions.pdf]

BMJ Open is committed to open peer review. As part of this commitment we make the peer review history of every article we publish publicly available.

When an article is published we post the peer reviewers' comments and the authors' responses online. We also post the versions of the paper that were used during peer review. These are the versions that the peer review comments apply to.

The versions of the paper that follow are the versions that were submitted during the peer review process. They are not the versions of record or the final published versions. They should not be cited or distributed as the published version of this manuscript.

BMJ Open is an open access journal and the full, final, typeset and author-corrected version of record of the manuscript is available on our site with no access controls, subscription charges or pay-per-view fees (http://bmjopen.bmj.com).

If you have any questions on BMJ Open's open peer review process please email info.bmjopen@bmj.com

BMJ Open

Public awareness of and responses to media coverage of invitation errors in the Breast Screening Programme in England: A population survey

Journal:	*BMJ Open*
Manuscript ID	bmjopen-2018-028040
Article Type:	Research
Date Submitted by the Author:	19-Nov-2018
Complete List of Authors:	Ghanouni, Alex; University College London, Research Department of Behavioural Science and Health<br>von Wagner, Christian; UCL, Research Department of Behavioural Science and Health<br>Waller, Jo; UCL, Research Department of Behavioural Science and Health
Keywords:	Organisation of health services < HEALTH SERVICES ADMINISTRATION & MANAGEMENT, JOURNALISM (see Medical Journalism), PUBLIC HEALTH, Breast imaging < RADIOLOGY & IMAGING

SCHOLARONE™
Manuscripts

FULL TITLE PAGE

Title: Public awareness of and responses to media coverage of invitation errors in the Breast Screening Programme in England: A population survey

Manuscript type: Research article

Authors:

Alex Ghanouni[a] a.ghanouni@ucl.ac.uk

Christian von Wagner[a] c.wagner@ucl.ac.uk

Jo Waller[ab] j.waller@ucl.ac.uk | Tel: +44 (0)20 7679 5958 | Fax: +44 (0)20 7679 8354

Affiliations:

[a]Research Department of Behavioural Science and Health, University College London, Gower Street, London, WC1E 6BT, United Kingdom

[b]Corresponding author

For peer review only

ABSTRACT

Objectives: In May 2018, the British Health Secretary announced the 'serious failure' that 450,000 women had missed out on invitations to breast screening in England, leading to extensive media coverage. This study measured public awareness of the story and tested for associated factors (e.g. educational level and trust in the NHS).

Design: A computer-assisted face-to-face survey in June 2018.

Setting: Participants completed the survey in their homes.

Participants: Males and females aged 16 years or older in England.

Primary and secondary outcome measures: Awareness of aspects of the media coverage and reported statistics. Other data included demographics (e.g. ethnicity), awareness of unrelated contemporaneous news stories, trust in participants' GPs and the NHS, and (among women) worry about breast cancer and future breast screening intentions.

Results: Descriptive statistics showed that 66.7% of 1,894 participants reported being aware of the media coverage. Regression analyses showed that those who were aware of other news stories, were white British, and had a higher level of education or social class grade were more likely to be aware. In contrast, only 36.0% correctly identified at least one of two headline statistics. This study did not find evidence that awareness was negatively associated with trust in participants' GPs or the NHS, breast cancer worry or future breast cancer screening intentions.

Conclusions: Awareness of the breast screening news story was high but recall of reported statistics was much lower: the public may have retained only the gist of quantitative information. Associations between story awareness and attitudes or behaviour were not apparent.

Keywords: Breast imaging; Journalism; Organisation of health services; Public Health

For peer review only

Strengths and limitations of this study:

- This study builds on previous research on media coverage around public health concerns by measuring levels of awareness among the general public and testing for characteristics associated with awareness.
- The survey was carried out shortly after media coverage of the announcement began, when awareness and knowledge were likely to be at their highest.
- Associations between awareness of media coverage and e.g. greater worry about breast cancer and lower trust in the NHS were not apparent but Type II error cannot be excluded.
- Tests for associations between awareness of media coverage and screening behaviour were based on intended future uptake; actual uptake may differ.

For peer review only

INTRODUCTION

On 2nd May 2018, the Health Secretary in Great Britain, Jeremy Hunt, made an unanticipated statement to the House of Commons regarding *“a serious failure…in the national Breast Screening Programme”*. Mr Hunt stated that since 2009, *“a computer algorithm failure”* had resulted in approximately 450,000 women not being invited to their final regular breast screening appointment (i.e. when they were aged 68 to 71 years). He indicated to the House that *“[the] current best estimate based on statistical modelling…is that there may be between 135 and 270 women who had their lives shortened as a result”* and that women affected *"will automatically be sent an invitation to a catch-up screening"*.[1]

News of this statement was reported extensively in the national media (e.g. [2-4]). Previous research has found that media coverage of cancer-related stories in the United Kingdom has appreciable public health implications. For example, there is evidence that the cervical cancer diagnosis and death of a young female celebrity, Jade Goody, influenced women’s cervical cancer screening decisions and temporarily increased uptake and diagnoses of high-grade cervical neoplasia.[5-8] Similarly, uptake of the colorectal screening programme increased following coverage of the United Kingdom Flexible Sigmoidoscopy Screening Trial.[9-10] Comparable findings have been reported by studies of pre-planned media messages such as Public Health England’s ‘Be Clear on Cancer’ campaigns, which

aim to increase cancer symptom awareness. These were associated with an increase in symptomatic attendance at General Practices and referrals to secondary care.[11-13]

In these cases, media coverage was associated with an increase in healthcare usage. However, news about an error in the screening programme may have had adverse effects (e.g. diminishing trust in the National Health Service). This presumes a nominal level of public awareness about the news story; it is unclear to what extent such health stories reach the general public and whether the public retains key information.

This study surveyed public awareness of the story and knowledge about the relevant statistics (i.e. the number of women estimated to have missed an invitation and to have had their lives shortened) shortly after the announcement. We also conducted an exploratory analysis of variables associated with awareness of the media coverage, including education, gender, and awareness of other news stories that were reported around the same time. We also tested the hypotheses that awareness of the breast screening media coverage would be associated with lower trust in participants' GPs and the NHS and (in women) more frequent worry about breast cancer and being less likely to intend to participate in breast screening.

METHODS

Design

Institutional ethical approval was obtained (registration number: 2951/006). A market research agency (Kantar TNS UK) collected data in two waves of sampling between 6th and 10th June 2018 (i.e. less than six weeks after the initial news story. The survey questions formed one module within a weekly face-to-face computer-assisted omnibus survey on a wider range of topics. Random location sampling was used to identify target households based on the 2011 Census and Postcode Address File. At each location, quotas were set with the aim of achieving national representativeness based on working status, children in the household, gender, and age.

The full survey is included in Appendix 1. Participants were initially shown a computer screen with text introducing the study and asking for their consent to participate. They were also given an information card containing debrief text and directions to further information about breast screening.

For peer review only

Participants

Eligible participants were all males and females in England aged 16 years or older who consented to take part in this module of the survey. The sample includes women eligible for breast screening (i.e. aged 47 to 73 years) and also members of the general population (males and females aged 16 years or older) since it was hypothesised that awareness of the story had the potential to negatively affect perceptions of other health services, irrespective of whether participants were affected directly.

Measures

Demographics:

General background information included participants' self-reported age (in years), gender, ethnic origin, marital status, education, social class grade,[14] employment status, and urban or rural area type.

Cancer and breast screening experience, and attitudes towards screening:

Participants were asked whether they had been diagnosed with any of several types of cancer themselves. Women aged 47 years or older were also asked if they had ever been i) invited to and ii) participated in the Breast Screening Programme.

Participants were asked about their attitudes towards screening via a previously used question,[15] *"routine screening means testing healthy people to find cancer before they have any symptoms. Do you think routine cancer screening tests for healthy people are almost always a good idea?"*. Response options were *"yes"*, *"no"*, and *"not sure"*.

Awareness of the breast screening news story:

Participants were asked to read a brief summary of the story (see Appendix 1, Q7), the main details of which were derived from the primary story on the topic on the BBC news website.[4] This was followed by the question, *"do you recall seeing or hearing anything about this news story before now?"*. Response options were *"yes"*, *"no"*, and *"not sure"*.

It was anticipated that directions of associations with awareness may depend on the specific parts of the story of which participants were aware. For example, follow-up commentaries on the main news

For peer review only

story argued that breast screening has no effect on all-cause mortality and results in overdiagnosis, which may have mitigated worry about screening errors. Consequently, participants who reported being aware of the main news story were also asked about their awareness of further reporting using two further summaries (see Appendix 1, Q14 and Q15), derived from two sources.[16-17]

Questions for assessing awareness were the same as previous. Participants reporting awareness of the news story were also asked where they saw or heard it and whether they discussed or shared it with anyone else. They were also asked two questions on the key statistics reported based on the following summaries:

"The Health Secretary, Jeremy Hunt, gave an estimate of the number of women who had failed to get invitations since 2009."

"The Health Secretary also gave an estimate, based on computer modelling, of the number of women who may have had their lives shortened."

For both, the question was *"which of the following do you think is the estimate that he gave?"*. For the first question, response options consisted of the true estimate (450,000) and three alternatives that were orders of magnitude higher or lower (4,500, 45,000, and 4,500,000). Similarly, response options for the second question consisted of the correct answer (between 135 and 270) and alternatives that were either an order of magnitude higher (1,350 and 2,700), lower (13 and 27), or both higher and lower (13 and 2,700). Response order was presented in one of two different ways for each participant (determined at random) to reduce potential order effects.

Awareness of news stories unrelated to breast screening:

Awareness of other news stories was measured by asking participants to read two further summaries (one on a volcano eruption in Hawaii; one on local council elections in England; see Appendix 1, Q19 and Q20). This was followed by the same measure of awareness as in previous questions. Main details were derived from the primary stories on the BBC news website.[18-19] These two stories were selected for comparison because they were reported around the same time and also consisted of specific, definable events.

Trust in health services:

For peer review only

Participants were asked two questions based on previously used items,[20] *"in general, how much do you trust…"* i) *"…your general practitioner?"* and ii) *"…the NHS?"*. Response options for both were *"not at all"*, *"a little"*, *"somewhat"*, *"a lot"*, and *"not sure"*.

Frequency of breast cancer worry:

Breast cancer worry (among women) was measured using an item based on one previously used,[21] *"how often do you worry about your chances of getting breast cancer yourself?"*. Response options were, *"never"*, *"occasionally"*, *"sometimes"*, *"often"*, *"very often"*, *"not sure"*, and *"prefer not to say"*.

Breast screening intentions:

Women aged 16 to 69 years were asked, *"do you think you will go for breast screening when you are next offered it?"*. Response options were *"yes, definitely"*, *"yes, probably"*, *"no, probably not"*, and *"no, definitely not"*.

Analysis

Participant characteristics and awareness about the news stories are reported using descriptive statistics. Responses of *"prefer not to say"* were excluded, as were responses of *"not sure"* for ordinal variables. Other responses of *"not sure"*, were grouped with *"no"*. Ethnicity was dichotomised into *"white British"* and *"other groups"*; social class grades were grouped into *"A or B"*, *"C1"*, *"C2"*, and *"D or E"*. For education, *"trade apprenticeships"* were grouped with *"other qualifications"*. Responses to measures of invitations to and participation in breast screening were coded into *"not eligible or not invited"*, *"invited, never taken part"*, and *"taken part"*.

One exploratory regression model tested for variables potentially associated with whether people responded to the survey. Three exploratory regression models tested for variables potentially associated with i) awareness of the breast screening news; and stating correctly the number of women who were ii) not invited for screening and iii) estimated to have had their lives shortened. A further four regression models tested the null hypotheses that awareness of the breast screening news story was not associated with trust in iv) participants' GPs and v) the NHS in the whole sample; and vi) frequency of worry about breast cancer and vii) intentions to participate in breast screening in future among women aged 70 years or less, after adjusting for covariates.

For peer review only

For the model assessing variables associated with responding to the questionnaire, the main variables of interest were recruitment wave, gender, ethnicity, marital status, social class grade, employment status, area type, and age (since these were the variables where data were available for both participants and non-participants). For the four main exploratory models and hypothesis testing models, independent variables were as above with the addition of other available measures (listed in appended tables) where multi-collinearity was not an appreciable issue (i.e. Variance Inflation Factors <10). Age was included in models as either a continuous variable or divided into age groups (where a Box-Tidwell procedure found evidence that the assumption of linearity was not met; $p<.05$). Frequency of worry about breast cancer was also included in the model of future breast screening intentions.

For models testing hypotheses, responses on measures of awareness of the breast screening story were coded into a single nominal variable with five levels: 1) *"unaware of the story"*, 2) *"aware of the main story only"*, 3) *"aware of the main story and all-cause mortality follow-up commentary"*,4) *"aware of the main story and overdiagnosis follow-up commentary"*, 5) *"aware of the main story and both follow-up commentaries"*.

Ordinal logistic regression was attempted in the first instance where dependent variables were ordinal. Tests of parallel lines suggested that the assumption of proportional odds was generally not met ($p<.0005$). Hence, dependent variables were dichotomised and binary logistic regression was used, except for the model testing associations with frequency of breast cancer worry where ordinal logistic regression was appropriate. Participants with missing data on variables of interest were not included in models.

RESULTS

Participant characteristics

2,681 participants began the survey module. 787 (29.4%) opted out, leaving 1,894 participants who provided data. Mean age was 50.8 years (standard deviation: 20.5). Characteristics are described in Appendix 2 (Table A). Response to the survey module questions was associated with all variables in the model, except for area type (Appendix 2, Table B). Participants of the omnibus survey approached were more likely to respond to this survey module if they were invited in wave 1 (vs. wave

For peer review only

2), female (vs. male), white British (vs. other groups), married, living as a couple, or widowed, divorced or separated (vs. single), in higher social class grades (vs. grades D or E), working (vs. not working), and younger.

Awareness of news stories, sources of information, and variables associated with awareness of the breast screening media coverage

1,264/1,894 (66.7%) reported being aware of the main news story (Appendix 2, Table A) and relatively few reported being aware of follow-up commentaries: 438/1,264 (34.7%) and 367/1,264 (29.0%) recognised the commentaries on all-cause mortality and overdiagnosis, respectively. 250/1,264 (19.8%) were aware of both. 971/1,264 (76.8%) and 271/1,264 (21.4%) encountered the story on television and radio, respectively (participants could select more than one). 169/1,264 (13.4%) and 134/1,264 (10.6%) encountered the story in print newspapers and online news websites (Appendix 2, Table C). Other news sources were used relatively rarely e.g. 68/1,264 (5.4%) heard the story from social media websites. 450/1,264 (35.6%) reported discussing or sharing the story with someone else.

Participants were more likely to be aware of the story if they were aware of either of the other two news stories. Awareness of the three stories was highly interrelated: 824/1,894 participants (43.5%) were aware of all three news stories and a further 196/1,894 (10.3%) reported not being aware of any. Only 323/1,894 (17.1%) were aware of just one of the three stories and only 106/1,894 participants (5.6%) were aware of the news about breast screening, specifically. Participants were also more likely to be aware of the breast screening news story if they were white British, older, had higher levels of education or social class grade. Participants were less likely to be aware if they believed that screening was almost always a good idea. All other p-values were ≥.207 (Table 1).

Awareness of statistics from the breast screening media coverage and variables associated with awareness

Only 233 (18.4%) of the 1,264 participants who reported being aware of the story correctly recognised the number of women who had not been invited and only 268 (21.2%) correctly recognised the estimated number of women who had their lives shortened. 809 (64.0%) did not correctly identify either statistic and only 3.6% correctly identified both (Table 2). The model testing for demographic

For peer review only

and psychological variables associated with correctly identifying either set of statistics found only weak evidence against the null hypothesis for all characteristics (p-values were ≥.087 and ≥.062 in the respective models; data not shown).

Awareness of media coverage and participants' trust their GPs and the NHS

In both these models, there was only weak evidence against the null hypothesis. Table 3 shows the main results of binary logistic regression models consisting of 1,746 participants (p=.729 and .290). Full results of the model are presented in Appendix 2 (Table D and Table E).

Awareness of media coverage and frequency of worry about breast cancer

Table 4 shows that there was only weak evidence against the null hypothesis (n=700; p=.084). Full results are included in Appendix 2 (Table F).

Awareness of media coverage and future breast screening intentions

Table 5 shows that there was only weak evidence against the null hypothesis for this analysis (n=700; p=.108). Full results are included in Appendix 2 (Table G).

For peer review only

Table 1 – Full results of the binary logistic regression model testing for variables associated with awareness of the breast screening news story

Characteristic	Total (n=1,792)	Aware vs. Not aware of the breast screening story (or not sure): n (%)<br>Not aware/sure (n=587; 32.8%)	Aware (n=1,205; 67.2%)	Adjusted OR, 95% CI<br>Aware of the screening story (vs. Not aware or not sure)	p-value
**Recruitment wave**					
Wave 2: 20-26th June	570	185 (32.5)	385 (67.5)	1.02, 0.79 to 1.31	.907
vs. Wave 1: 6-10th June	1,222	402 (32.9)	820 (67.1)		
**Age**					Overall: **<.0005**
65+	549	111 (20.2)	438 (79.8)	7.77, 4.52 to 13.38	**<.0005**
55-64	252	53 (21.0)	199 (79.0)	6.75, 3.92 to 11.63	**<.0005**
45-54	241	47 (19.5)	194 (80.5)	7.70, 4.56 to 13.00	**<.0005**
35-44	248	88 (35.5)	160 (64.5)	3.60, 2.22 to 5.84	**<.0005**
25-34	275	142 (51.6)	133 (48.4)	2.00, 1.27 to 3.14	**.003**
vs. 16-24	227	146 (64.3)	81 (35.7)		
**Gender**					
Male	771	234 (30.4)	537 (69.6)	1.00, 0.74 to 1.35	.999
vs. Female	1,021	353 (34.6)	668 (65.4)		
**Ethnicity**					
White British	1,491	415 (27.8)	1,076 (72.2)	3.00, 2.20 to 4.09	**<.0005**
vs. Other groups	301	172 (57.1)	129 (42.9)		
**Marital status**					Overall: .914
Married/Living as a couple	985	279 (28.3)	706 (71.7)	1.07, 0.78 to 1.47	.672
Widowed/Divorced/Separated	354	84 (23.7)	270 (76.3)	1.06, 0.70 to 1.60	.792
vs. Single	453	224 (49.4)	229 (50.6)		
**Highest level of education**					Overall: **.001**
Graduate level/Above	501	131 (26.1)	370 (73.9)	2.08, 1.34 to 3.23	**.001**
A-levels/AS levels/Equivalents	448	162 (36.2)	286 (63.8)	1.80, 1.19 to 2.73	**.006**
GCSEs/Equivalents	440	156 (35.5)	284 (64.5)	1.36, 0.92 to 2.00	.120
Trade apprenticeships/Other	89	39 (43.8)	50 (56.2)	0.75, 0.42 to 1.32	.316
vs. No formal qualifications	314	99 (31.5)	215 (68.5)		
**Social class grade**					Overall: **<.0005**
Grade A or B	326	53 (16.3)	273 (83.7)	2.44, 1.59 to 3.73	**<.0005**
Grade C1	511	165 (32.3)	346 (67.7)	1.41, 1.02 to 1.95	**.037**
Grade C2	394	142 (36.0)	252 (64.0)	1.13, 0.81 to 1.58	.469
vs. Grade D or E	561	227 (40.5)	334 (59.5)		
**Employment status**					
Working	823	287 (34.9)	536 (65.1)	0.91, 0.68 to 1.22	.909
vs. Not working	969	300 (31.0)	669 (69.0)		
**Area type**					
Urban	1,458	476 (32.6)	982 (67.4)	1.21, 0.90 to 1.64	.207
vs. Rural	334	111 (33.2)	223 (66.8)		
**Personal diagnosis of cancer**					
Yes	150	34 (22.7)	116 (77.3)	1.18, 0.74 to 1.86	.490
vs. No	1,642	553 (33.7)	1,089 (66.3)		
**Personal experience of breast screening**					Overall: .552
Taken part	425	90 (21.2)	335 (78.8)	0.92, 0.60 to 1.41	.705
Invited, never taken part	55	13 (23.6)	42 (76.4)	0.66, 0.32 to 1.39	.276
vs. Not eligible or not invited	1,312	484 (36.9)	828 (63.1)		
**Belief that screening is almost always a good idea**					
Yes	1,649	547 (33.2)	1,102 (66.8)	0.59, 0.38 to 0.94	**.025**
vs. No or not sure	143	40 (28.0)	103 (72.0)		
**Awareness of volcano news**					
Yes	1,367	325 (23.8)	1,042 (76.2)	3.14, 2.39 to 4.12	**<.0005**
vs. No or not sure	425	262 (61.6)	163 (38.4)		
**Awareness of election news**					
Yes	1,138	292 (25.7)	846 (74.3)	1.37, 1.06 to 1.75	**.014**
vs. No or not sure	654	295 (45.1)	359 (54.9)		
**General level of trust in NHS**					Overall: .485
A lot	969	308 (31.8)	661 (68.2)	0.59, 0.29 to 1.18	.132
Somewhat	599	193 (32.2)	406 (67.8)	0.63, 0.31 to 1.27	.196
A little	169	69 (40.8)	100 (59.2)	0.58, 0.27 to 1.25	.166

For peer review only

vs. Not at all	55	17 (30.9)	38 (69.1)	

Table 2 – Descriptive statistics of participants' responses about key statistics in the breast screening media coverage; correct responses were "450,000" and "135-270"

	n (% of total; 95% CI) (n=1,264)					
**Number of women who did not receive their final invitation…**	**Number of women who may have had their life shortened. Between…**					
	135 - 270	*13 - 27*	*13 - 2,700*	*1,350 - 2,700*	*Not sure*	*Total*
450,000	46 (3.6)	6 (0.5)	79 (6.3)	71 (5.6)	31 (2.5)	233 (18.4)
4,500	68 (5.4)	20 (1.6)	28 (2.2)	22 (1.7)	30 (2.4)	168 (13.3)
45,000	130 (10.3)	22 (1.7)	76 (6.0)	86 (6.8)	54 (4.3)	368 (29.1)
4,500,000	3 (0.2)	1 (0.1)	10 (0.8)	20 (1.6)	4 (0.3)	38 (3.0)
Not sure	21 (2.1)	5 (0.4)	15 (1.2)	12 (0.9)	404 (32.0)	457 (36.2)
Total	268 (21.2)	54 (4.3)	208 (16.5)	211 (16.7)	523 (41.4)	

Table 3 – Testing for an association between awareness of the breast screening media coverage and trust in i) participants' GPs and ii) the NHS*

**Trust in participants' GPs**		**A lot vs. Not at all; a little; somewhat: n (%)**		**Adjusted OR, 95% CI**	**p-value**
**Characteristic**	**Total (n=1,746)**	**Less than a lot (n=781; 44.7%)**	**A lot (n=965; 55.3%)**	**A lot (vs. Less than a lot)**	
**Screening story awareness**					Overall: .729
Aware of the main story and both follow-up commentaries	238	98 (41.2)	140 (58.8)	1.10, 0.74 to 1.64	.653
Aware of the main story and overdiagnosis follow-up	172	66 (38.4)	106 (61.6)	1.31, 0.85 to 2.03	.218
Aware of the main story and all-cause mortality follow-up	107	49 (45.8)	58 (54.2)	1.21, 0.73 to 2.02	.459
Aware of the main story only	655	280 (42.7)	375 (57.3)	1.17, 0.88 to 1.57	.283
vs. Unaware of the story	574	288 (50.2)	286 (49.8)		
**Trust in the NHS**		**A lot vs. Not at all; a little; somewhat: n (%)**		**Adjusted OR, 95% CI**	**p-value**
**Characteristic**	**Total (n=1,746)**	**Less than a lot (n=803; 46.0%)**	**A lot (n=943; 54.0%)**	**A lot (vs. Less than a lot)**	
**Screening story awareness**					Overall: .290
Aware of the main story and both follow-up commentaries	238	102 (42.9)	136 (57.1)	0.87, 0.59 to 1.30	.503
Aware of the main story and overdiagnosis follow-up	172	76 (44.2)	96 (55.8)	0.78, 0.51 to 1.21	.267
Aware of the main story and all-cause mortality follow-up	107	57 (53.3)	50 (46.7)	0.58, 0.35 to 0.97	.039
Aware of the main story only	655	299 (45.6)	356 (54.4)	0.81, 0.60 to 1.09	.160
vs. Unaware of the story	574	269 (46.9)	305 (53.1)		

*Results are adjusted for covariates; full results of the model are reported in the Appendix

For peer review only

Table 4 – Testing for an association between awareness of the breast screening media coverage and frequency of breast cancer worry*

Characteristic	Total (n=700)	Frequency of worry about breast cancer: n (%) Never (n=210; 30.0%)	Occasionally (n=231; 33.0%)	Sometimes (n=177; 25.3%)	Often (n=43; 6.1%)	Very often (n=39; 5.6%)	Adjusted OR, 95% CI — More frequent worry about breast cancer	p-value
**Screening story awareness**								Overall: .084
Aware of the main story and both follow-up commentaries	88	32 (36.4)	33 (37.5)	15 (17.0)	2 (2.3)	6 (6.8)	0.94, 0.56 to 1.56	.797
Aware of the main story and overdiagnosis follow-up	63	22 (34.9)	20 (31.7)	16 (25.4)	2 (3.2)	3 (4.8)	0.97, 0.56 to 1.68	.917
Aware of the main story and all-cause mortality follow-up	36	10 (27.8)	15 (41.7)	6 (16.7)	2 (5.6)	3 (8.3)	1.40, 0.71 to 2.78	.329
Aware of the main story only	270	64 (23.7)	89 (33.0)	74 (27.4)	23 (8.5)	20 (7.4)	1.51, 1.05 to 2.16	.025
vs. Unaware of the story	243	82 (33.7)	74 (30.5)	66 (27.2)	14 (5.8)	7 (2.9)		

*Results are adjusted for covariates; full results of the model are reported in the Appendix

Table 5 – Testing for an association between awareness of the breast screening media coverage and breast screening intentions*

Characteristic	Total (n=700)	Yes, definitely vs. Yes, probably; no, probably not; no, definitely not: n (%) No definite intention (n=99; 14.1%)	Definite intention (n=601; 85.9%)	Adjusted OR, 95% CI — Definite intention (vs. No definite intention)	p-value
**Screening story awareness**					Overall: .108
Aware of the main story and both follow-up commentaries	88	10 (11.4)	78 (88.6)	2.01, 0.74 to 5.48	.172
Aware of the main story and overdiagnosis follow-up	63	4 (4.3)	59 (93.7)	2.66, 0.79 to 8.89	.113
Aware of the main story and all-cause mortality follow-up	36	6 (16.7)	30 (83.3)	0.66, 0.20 to 2.13	.486
Aware of the main story only	270	22 (8.1)	248 (91.9)	1.88, 0.99 to 3.57	.054
vs. Unaware of the story	243	57 (23.5)	186 (76.5)		

*Results are adjusted for covariates; full results of the model are reported in the Appendix

For peer review only

DISCUSSION

Previous studies have found evidence that media messages can increase usage of a range of healthcare services (e.g. [5-8, 10-13]). Awareness of this story about errors in the breast screening programme was hypothesised to be associated with lower trust in the NHS. However, the results of this study did not provide strong evidence against the null hypothesis for this or any associations tested. This may be a reflection of the news not undermining confidence in the Breast Screening Programme, or causing concern about breast cancer among women. If so, this might be partly attributable to the news story saying little to reduce the perceived benefits of breast screening itself, in contrast to media coverage of e.g. the independent review of breast cancer screening, which reported on the issue of overdiagnosis extensively.[22-23] Relatedly, the present study found that awareness was notably lower for follow-up commentaries on the shortcomings of breast screening, compared with the main story. In addition, the framing of the story may have been expected to reinforce the perceived benefits of screening by indicating that missing screening had negative consequences in terms of additional breast cancer deaths.

Population awareness of the breast screening news story was generally high; television and radio were the main sources of information. This is broadly consistent with patterns of how most news is accessed, although the internet was used less often than observed in previous surveys.[24] Awareness of this story was related to awareness of other news stories, suggesting that an appreciable proportion of the population can be broadly dichotomised into those who are generally “news aware” and “news unaware”. These results do not suggest that a notable proportion of the public are aware of health news, specifically. In contrast to these findings, recall of the main statistics was markedly low and correct responses may be largely attributable

For peer review only

to random guessing.[1] In some respects, this is surprising since the statistics were an integral part of the story and often part of headlines (e.g. [2-4, 25]). This may suggest that people either tend not to attend to or memorise this statistical information (meaning that they would not be able to factor it into their appraisal of the significance of the story) or they retain only the 'gist' of the statistics involved.[26] Awareness of the breast screening story was greater among those with higher levels of education and social class grade, those who were white British, and those who were older. Awareness of the breast screening news story was also lower among participants with positive attitudes towards screening (who may have been less likely to attend to a negative story).

This study has limitations. Despite the large sample size and adjustment for a range of potentially confounding variables, some odds ratios could not be estimated with a high degree of precision. Confidence intervals were wide for key variables, meaning that associations may not have been detected if they were real but smaller than observed. In addition, findings on screening uptake only relate to anticipated future behaviour; future research could build on this study by assessing whether the announcement was followed by a decrease (or increase) in actual screening uptake. Members of the public were also less likely to participate in the survey module based on a range of characteristics for which data were available. Results may be biased, insofar as responses differed based on these variables.

For peer review only

---

[1] Participants were asked additional questions on the extent to which they trusted the statistics and their reasons for not trusting them (if applicable). However, since responses were highly suggestive of random guessing, no further analyses of these measures were attempted.

Conclusions

This study found that news of errors in the Breast Screening Programme in England had reached a large proportion of the general public and that those aware of the media coverage tended to be those aware of news stories in general. The proportion of people aware was also higher among those who had more education, were in a higher social class grade, or were older. In contrast, awareness of key statistics from the story was very low among participants aware of the story, even less than six weeks after the onset of the main media coverage. The results of this study did not provide evidence that media coverage had any effects on trust in aspects of the health service among the general public, or worry about breast cancer or breast screening intentions among women. Future research should investigate possible effects of media coverage using objective measures of screening behaviour.

Contributors: AG, CVW, and JW conceived and designed the study. AG analysed the data. AG, CVW, and JW participated in the interpretation of results. AG, CVW, and JW drafted the manuscript, participated in critical revision, and approved the final version.

Funding: This work was supported by a programme grant from Cancer Research UK awarded to Prof Jane Wardle [C1418/A14134]. Dr Jo Waller is supported by a Career Development Fellowship from Cancer Research UK [C7492/A17219]. Cancer Research UK was not involved in the design of this study; the collection, analysis, or interpretation of the results; in the writing of the manuscript; or in the decision to submit for publication.

Competing interests: None declared.

Patient consent: Obtained.

Data sharing: No additional data are available.

For peer review only

REFERENCES

1. House of Commons Hansard. Breast cancer screening https://hansard.parliament.uk/commons/2018-05-02/debates/BE9DB48A-C9FF-401B-AC54-FF53BC5BD83E/BreastCancerScreening (2018, accessed 18 October 2018).
2. Mail Online. 'They didn't deserve to lose their lives': Fury of husband whose wife died from breast cancer after 'colossal' NHS computer glitch which has seen 270 die and 450,000 miss screenings https://www.dailymail.co.uk/news/article-5682203/Hundreds-women-developed-breast-cancer-450-000-not-invited-screening.html (2018, accessed 18 October 2018).
3. The Guardian. Up to 270 women may have died after breast cancer screening IT error https://www.theguardian.com/society/2018/may/02/jeremy-hunt-to-launch-inquiry-into-450000-missed-breast-cancer-screenings (2018, accessed 18 October 2018).
4. BBC News. Breast screening error 'shortened up to 270 lives' – Hunt https://www.bbc.co.uk/news/health-43973652 (2018, accessed 18 October 2018).
5. Lancucki L, Sasieni P, Patnick J, et al. The impact of Jade Goody's diagnosis and death on the NHS Cervical Screening Programme. J Med Screen 2012; 19:89–93.
6. MacArthur GJ, Wright M, Beer H, et al. Impact of media reporting of cervical cancer in a UK celebrity on a population-based cervical screening programme. J Med Screen 2011; 18:204–209.
7. Marlow LAV, Sangha A, Patnick J, et al. The Jade Goody Effect: Whose cervical screening decisions were influenced by her story? J Med Screen 2012; 19:184–188.
8. Casey GM, Morris B, Burnell M, et al. Celebrities and screening: a measurable impact on high-grade cervical neoplasia diagnosis from the "Jade Goody effect" in the UK. Br J Cancer 2013; 109:1192–1197.

For peer review only

9. Atkin WS, Edwards R, Kralj-Hans I, et al. Once-only flexible sigmoidoscopy screening in prevention of colorectal cancer: a multicentre randomised controlled trial. Lancet 2010; 375:1624–1633.
10. Lo S, Vart G, Snowball J, et al. The impact of media coverage of the Flexible Sigmoidoscopy Trial on English colorectal screening uptake. J Med Screening 2012; 19:83–88.
11. Moffat J, Bentley A, Ironmonger L, et al. The impact of national cancer awareness campaigns for bowel and lung cancer symptoms on sociodemographic inequalities in immediate key symptom awareness and GP attendances. Br J Cancer 2015; 112:S14–S21.
12. Bethune R, Marshall MJ, Mitchell SJ, et al. Did the "Be Clear on Bowel Cancer" public awareness campaign pilot result in a higher rate of cancer detection? Postgrad Med J 2013; 89:390–393.
13. Hughes-Hallett A, Browne D, Mensah E, et al. Assessing the impact of mass media public health campaigns. Be Clear on Cancer "blood in pee": a case in point. BJU Int 2016; 117:570–575.
14. National Readership Survey. Social Grade http://www.nrs.co.uk/nrs-print/lifestyle-and-classification-data/social-grade (n.d., accessed 18 October 2018).
15. Schwartz LM, Woloshin S, Fowler FJ, Welch HG. Enthusiasm for cancer screening in the United States. J Am Med Assoc. 2004;291(1):71–8.
16. Bewley S, Baum M, Hodkinson A, et al. Screening 'flaw'. The Times, 05 May 2018
17. Spiegelhalter D. Have 'up to 270 women died' by missing a breast screening appointment letter? https://medium.com/wintoncentre/have-up-to-270-women-died-by-missing-a-breast-screening-appointment-letter-756f74c4f56b (2018, 18 October 2018).

For peer review only

18. BBC News. Local election results 2018: No clear winner as Labour and Tories neck and neck https://www.bbc.co.uk/news/uk-politics-44014076 (2018, 18 October 2018).

19. BBC News. Kilauea: Hawaii emergency declared over volcano eruption https://www.bbc.co.uk/news/world-us-canada-44001651 (2018, 18 October 2018).

20. Health Information National Trends Survey 2015 and 2017 https://hints.cancer.gov (2018, 18 October 2018).

21. Health Information Trends Survey 2003, 2005, and 2008 https://hints.cancer.gov (2018, 18 October 2018).

22. Independent UK Panel on Breast Cancer Screening. The benefits and harms of breast cancer screening: an independent review. Lancet 2012; 380:1778-1786.

23. BBC News. Breast screening advice updated amid controversy over tests https://www.bbc.co.uk/news/health-20121043 (2012, 18 October 2018).

24. OFCOM. News consumption in the UK https://www.ofcom.org.uk/research-and-data/tv-radio-and-on-demand/news-media/news-consumption (2018, 18 October 2018).

25. Sky News. Breast cancer screening failure ‘shortened’ up to 270 lives https://news.sky.com/story/inquiry-into-breast-cancer-screening-failures-to-be-launched-say-sky-sources-11355751 (2018, 18 October 2018).

26. Reyna VF, Brainerd CJ. Fuzzy-trace theory. An interim synthesis. Learn Individ Differ. 1997; 7:1–75.

For peer review only

APPENDIX 1 - SURVEY

[All Adults 16+ in England. Participants were shown the tablet screen and the following text was read out by interviewers]

Q.A In this part of the survey, I am going to ask you some questions related to health, including cancer, and recent news stories. These questions are asked on behalf of researchers from University College London. If you do not wish to answer a particular question during any part of this survey, you may refuse to answer and we will move to the next question. All your answers will be kept strictly confidential and you will be anonymous to the researchers.

The NHS currently offers breast cancer screening with mammography once every three years, to women aged between about 50 to 70 years in England.

Are you okay to continue with these questions?

1: Yes

2: No

As the questions can be perceived as sensitive, you can answer the questions on this machine yourself. I would now like to show you how to use the machine by going through a practice question with you.

[All Adults 16+ in England willing to continue. “Don’t know”/“Not sure”/“Prefer not to say” appeared at the top of the screen, out of view of participants, except for questions that participants completed themselves. Interviewers showed the screen to participants]

This is an example of a single-coded question

Q.B What is your favourite colour?

1: Red

2: Yellow

For peer review only

3: Blue

4: Green

Other colour (PEN -WRITE IN)

Don't know

Refused

[All females aged 16-69 in England willing to continue. Interviewers handed tablets to participants and stepped away from viewing the screen]

Q.1 Do you think you will go for breast screening when you are next offered it? REMEMBER TO TAP OK TO CONTINUE

1: Yes, definitely

2: Yes, probably

3: No, probably not

4: No, definitely not

Not sure

Prefer not to say

[All females aged 47+ in England willing to continue]

Q.2 Have you ever been invited for breast screening before? If you've only ever been offered a mammogram to investigate symptoms separately to the screening programme, please respond 'no'.

1: Yes

2: No

For peer review only

Not sure

Prefer not to say

[All females aged 47+ in England who have been invited for breast screening before]

Q.3 Have you ever been for breast screening as part of the screening programme?

1: Yes

2: No

Not sure

Prefer not to say

[All Adults 16+ in England willing to continue. This question allowed more than one response option. “None of the above”/”Prefer not to say” were mutually exclusive with other responses]

Q.4 Which of the following, if any, have you been diagnosed with? Please choose all that apply.

1: Bowel cancer

2: Lung cancer

3: Breast cancer

4: Cervical cancer

5: Prostate cancer

Other type of cancer - PEN WRITE IN

None of the above

Prefer not to say

For peer review only

[All Adults 16+ in England willing to continue. This question allowed more than one response option. “None of the above”/”Prefer not to say” were mutually exclusive with other responses]

Q.5 Has anyone you know ever been diagnosed with breast cancer? Please choose all that apply.

1: A close family member

2: Any other family member

3: A friend

4: A colleague

5: Any other person

Not sure

Prefer not to say

[All females aged 16+ in England willing to continue]

Q.6 How often do you worry about your chances of getting breast cancer yourself?

1: Never

2: Occasionally

3: Sometimes

4: Often

5: Very often

Not sure

Prefer not to say

Thank you for answering these questions - this is the end of this section for you.

For peer review only

[All Adults 16+ in England willing to continue. Participants handed the tablet back to the interviewer, who showed the screen and either read out or allowed participants to read subsequent questions]

Q.7 In May, it was reported that a computer algorithm failure had meant that a number of women did not receive invitations to their final routine breast cancer screening. The Health Secretary, Jeremy Hunt, said that women affected will be contacted by letter with an invitation for a catch-up screening test but some of the women who were not invited for their final appointment may have had their lives shortened.

Do you recall seeing or hearing anything about this news story before now?

1: Yes

2: No

Not sure

[All who recall seeing or hearing anything about this news story before now. This was a multiple choice question. The order of response options was randomised with “other websites” always following both “online news websites” and “social media websites”]

Q.8 Do you recall where you saw or heard this news story? Please choose all that apply.

1: Television

2: Print newspaper(s)

3: Radio

4: Online news websites

5: Social media websites

6: Other websites

For peer review only

7: Word of mouth

Other sources – PEN WRITE IN

Not sure

[All who recall seeing or hearing anything about this news story before now]

Q.9 Did you discuss or share the story with anyone else?

1: Yes

2: No

Not sure

[All who recall seeing or hearing anything about this news story before now. Participants were randomised to one of two orders of response options (1:1)]

Q.10 The Health Secretary, Jeremy Hunt, gave an estimate of the number of women who had failed to get invitations since 2009.

Which of the following do you think is the estimate that he gave?

1: 4,500 women

2: 45,000 women

3: 450,000 women

4: 4,500,000 women

Not sure

For peer review only

[All who recall seeing or hearing anything about this news story before now. Participants randomised to one of two orders of response options (1:1)]

Q.11 The Health Secretary also gave an estimate, based on computer modelling, of the number of women who may have had their lives shortened.

Which of the following do you think is the estimate that he gave?

1: Between 13 and 27 women

2: Between 135 and 270 women

3: Between 13 and 2,700 women

4: Between 1,350 and 2,700 women

Not sure

[All who gave an estimate in Q1 or Q11]

Q.12 How much did you trust these statistics when you heard them in the news?

1: Not at all

2: A little

3: Somewhat

4: A lot

Not sure

[All who do not trust the statistic]

Q.13 What were your reasons for not trusting these statistics when you heard them in the news?

PROBE: Any other reasons?

For peer review only

OPEN ENDED

[All who recall seeing or hearing anything about this news story before now]

Q.14 It was also reported that some health experts have said breast cancer screening can do “more harm than good” because they believe “breast screening…has no impact on all-cause death”.

Do you recall seeing or hearing anything about this aspect of the news story before now?

1: Yes

2: No

Not sure

[All who recall seeing or hearing anything about this news story before now]

Q.15 The estimate of the number of women who may have had their lives shortened that the Health Secretary gave was between 135 and 270. It was also reported that one statistics expert has said this claim is “misleading” because they believe “there is only weak evidence that screening helps prolong life, particularly for older women” and that “contrary to popular belief, screening also does harm...for every 200 women attending screening between 50 and 70, we would expect one to have her early death from breast cancer prevented, but three to be unnecessarily treated for a harmless cancer that would not have troubled them".

Do you recall seeing or hearing anything about this aspect of the news story before now?

1: Yes

2: No

Not sure

For peer review only

[All Adults 16+ in England willing to continue]

Q.16 In general, how much do you trust your general practitioner?

1: Not at all

2: A little

3: Somewhat

4: A lot

Not sure

[All Adults 16+ in England willing to continue]

Q.17 In general, how much do you trust the NHS?

1: Not at all

2: A little

3: Somewhat

4: A lot

Not sure

[All Adults 16+ in England willing to continue]

Q.18 Routine screening means testing healthy people to find cancer before they have any symptoms.

Do you think routine cancer screening tests for healthy people are almost always a good idea?

1: Yes

2: No

For peer review only

Not sure

[Participants were handed an information card with the following text and asked to read it]

You may have some questions about breast cancer screening after this part of the survey. You can find out more by calling the NHS on a Freephone number (0800 169 2692) or via the web on https:\\www.nhs.uk\conditions\breast-cancer-screening\missed-invitations\.

[All Adults 16+ in England willing to continue]

Q.19 It was also reported in May that a volcano had erupted in Hawaii, leading to officials declaring a state of emergency and mandatory evacuation of 1,700 residents in the area.

Do you recall seeing or hearing anything about this news story before now?

1: Yes

2: No

Not sure

[All Adults 16+ in England willing to continue]

Q.20 The results of local elections held in England were also reported in May. The Labour Party won 2,350 seats, the Conservative Party won 1,332 seats, and the Liberal Democrats won 536 seats.

Do you recall seeing or hearing anything about this news story before now?

1: Yes

2: No

Not sure

For peer review only

[All Adults 16+ in England willing to continue]

Q.21 Please can I ask you what is the highest level of qualification you have received?

1: A. Graduate level qualifications and above: including higher degrees, professional qualifications at HE standard (e.g. chartered accountant, surveyor, Nursing, Teaching), NVQ and SVQ Level 4 or 5, Higher Education Diplomas, HNC and HND and BTEC Higher, RSA Higher Diploma

2: B. A-levels and AS levels and equivalents: including SCE Higher, Scottish Certificate 6th Year Studies, NVQ and SVQ and GSVQ level 3, GNVQ Advanced, ONC and OND and BTEC National, City and Guilds Advanced Craft, City and Guilds Final level or Part III, RSA Advanced Diploma

3: C. Trade apprenticeships

4: D. GCSEs and equivalents: including O level, SCE Standard, CSEs, NVQ and SVQ and GSVQ level 1 and 2, GNVQ and BTEC and SCOTVEC first, General diploma, City and Guilds Ordinary level, City and Guilds Ordinary level Part II, RSA State I-III or Diploma, SCOTVEC modules

5: E. Other qualifications (including overseas)

6: F. No formal qualifications

Don’t know

Refused

For peer review only

APPENDIX 2 – SUPPLMENTARY TABLES

Table A – Summary statistics describing the sample

Measure	Total* (n=1,894)	%	(95% CI)
**Awareness of the news about breast screening**			
Aware of the main story and both follow-up commentaries	250	13.2	11.7 to 14.8
Aware of the main story and overdiagnosis follow-up	188	9.9	8.6 to 11.3
Aware of the main story and all-cause mortality follow-up	117	6.2	5.2 to 7.3
Aware of the main story only	709	37.4	35.3 to 39.6
Unaware of the story	630	33.3	31.2 to 35.4
**Recruitment wave**			
Wave 2: 20-26th June	606	32.0	29.9 to 34.1
Wave 1: 6-10th June	1,288	68.0	65.9 to 70.1
**Gender**			
Male	801	42.3	40.1 to 44.5
Female	1,093	57.7	55.5 to 59.9
**Ethnicity**			
White British	1,555	82.4	80.7 to 84.1
Other groups	331	17.6	15.9 to 19.3
**Marital status**			
Married or living as a married	1,039	54.9	52.6 to 57.1
Widowed, divorced and separated	382	20.2	18.4 to 22.0
Single	473	25.0	23.1 to 27.0
**Highest level of education**			
Graduate level qualifications and above	530	28.2	26.2 to 30.3
A-levels and AS levels and equivalents	461	24.6	22.7 to 26.5
GCSEs and equivalents	459	24.5	22.5 to 26.4
Trade apprenticeships or other qualifications	93	5.0	4.0 to 6.0
No formal qualifications	334	17.8	16.1 to 19.6
**Social class grade**			
Grade A or B	336	17.7	16.1 to 19.5
Grade C1	539	28.5	26.5 to 30.5
Grade C2	423	22.3	20.5 to 24.3
Grade D or E	596	31.5	29.4 to 33.6
**Employment status**			
Working	862	45.5	43.3 to 47.8
Not working	1,032	54.5	52.2 to 56.7
**Area type**			
Urban	1,542	81.4	79.6 to 83.1
Rural	352	18.6	16.9 to 20.4
**Personal diagnosis of cancer**			
Yes	156	8.4	7.2 to 9.7
No	1,705	91.6	90.3 to 92.8
**Personal experience of breast screening**			
Taken part	441	23.7	21.8 to 25.7
Invited, never taken part	56	3.0	2.3 to 3.9
Not eligible or not invited	1,364	73.3	71.2 to 75.3
**Belief that screening is almost always a good idea**			
Yes	1,737	91.7	90.4 to 92.9
No or not sure	157	8.3	7.1 to 9.6
**Awareness of the news about the volcanic eruption**			
Yes	1,435	75.8	73.8 to 77.7
No or not sure	459	24.2	22.3 to 26.2
**Awareness of the news about the local elections**			
Yes	1,198	63.3	61.1 to 65.4
No or not sure	696	36.7	34.6 to 38.9
**General level of trust in GP**			
A lot	1,009	55.2	52.9 to 57.5
Somewhat	540	29.6	27.5 to 31.7
A little	219	12.0	10.6 to 13.5
Not at all	59	3.2	2.5 to 4.1
**General level of trust in NHS**			
A lot	1,016	54.2	54.9 to 59.5
Somewhat	619	33.0	32.7 to 37.1

For peer review only

For peer review only - http://bmjopen.bmj.com/site/about/guidelines.xhtml

A little	184	9.8	9.0 to 11.8
Not at all	56	3.0	2.4 to 4.0
**Frequency of worry about breast cancer**			
Very often	46	4.6	3.4 to 6.0
Often	55	5.4	4.2 to 7.0
Sometimes	216	21.4	18.9 to 24.0
Occasionally	302	29.9	27.1 to 32.8
Never	391	38.7	35.7 to 41.7
**Breast screening intentions for next invitation**			
Yes, definitely	690	84.7	82.1 to 87.0
Yes, probably	88	10.8	8.8 to 13.1
No, probably not	18	2.2	1.4 to 3.4
No, definitely not	19	2.3	1.5 to 3.5

Table B – Full results of the binary logistic regression model testing for variables associated with whether participants responded to questions on the survey module

		**Responded vs. Did not respond to the survey questions: n (%)**		**Adjusted OR, 95% CI**	**p-value**
**Characteristic**	**Total (n=2,665)**	**Did not respond (n=779; 29.2%)**	**Responded (n=1,886; 70.8%)**	**Responded to questions (vs. Did not respond)**	
**Recruitment wave**					
Wave 2: 20-26th June	908	303 (33.4)	605 (66.6)	0.73, 0.61 to 0.87	**<.0005**
vs. Wave 1: 6-10th June	1,757	476 (27.1)	1,281 (72.9)		
**Gender**					
Male	1,270	474 (37.3)	796 (62.7)	0.46, 0.39 to 0.55	**<.0005**
vs. Female	1,395	305 (21.9)	1,090 (78.1)		
**Ethnicity**					
White British	2,139	584 (27.3)	1,555 (72.7)	1.69, 1.37 to 2.10	**<.0005**
vs. Other groups	526	195 (37.1)	331 (62.9)		
**Marital status**					Overall: **.001**
Married/Living as a couple	1,441	407 (28.2)	1,034 (71.8)	1.48, 1.18 to 1.85	**.001**
Widowed/Divorced/Separated	517	135 (26.1)	382 (73.9)	1.65, 1.21 to 2.24	**.002**
vs. Single	707	237 (33.5)	470 (66.5)		
**Social class grade**					Overall: **.003**
Grade A or B	450	115 (25.6)	335 (74.4)	1.54, 1.18 to 2.02	**.002**
Grade C1	726	190 (26.2)	536 (73.8)	1.44, 1.15 to 1.81	**.002**
Grade C2	596	174 (29.2)	422 (70.8)	1.28, 1.01 to 1.63	**.045**
vs. Grade D or E	893	300 (33.6)	593 (66.4)		
**Employment status**					
Working	1,225	366 (29.9)	859 (70.1)	0.79, 0.65 to 0.97	**.026**
vs. Not working	1,440	413 (28.7)	1,027 71.3)		
**Area type**					
Urban	2,164	629 (29.1)	1,535 (70.9)	1.14, 0.91 to 1.42	.246
vs. Rural	501	150 (29.9)	351 (70.1)		
**Age (in years)**	2,665	52.1 (21.0)	50.8 (20.5)	0.99, 0.98 to 1.00	**<.0005**

Table C – Sources of news about the breast screening story

**Source of information**	**Total (n=1,264)**	**%**	**(95% CI)**
Television	971	76.8	74.4 to 79.1
Radio	271	21.4	19.2 to 23.8
Print newspaper(s)	169	13.4	11.6 to 15.3
Online news websites	134	10.6	9.0 to 12.4
Social media websites	68	5.4	4.2 to 6.7
Other websites	11	0.9	0.5 to 1.5
Word of mouth	43	3.4	2.5 to 4.5
Other sources	8	0.6	0.3 to 1.2
Discussed or shared the story with someone else	450	35.6	33.0 to 38.3

For peer review only

Table D – Full results of the binary logistic regression model testing for an association between awareness of the breast screening media coverage and trust in participants' GPs

		A lot vs. Not at all; a little; somewhat: n (%)		Adjusted OR, 95% CI	p-value
**Characteristic**	**Total (n=1,746)**	**Less than a lot (n=781; 44.7%)**	**A lot (n=965; 55.3%)**	**A lot (vs. Less than a lot)**	
**Screening story awareness**					Overall: .729
Aware of the main story and both follow-up commentaries	238	98 (41.2)	140 (58.8)	1.10, 0.74 to 1.64	.653
Aware of the main story and overdiagnosis follow-up	172	66 (38.4)	106 (61.6)	1.31, 0.85 to 2.03	.218
Aware of the main story and all-cause mortality follow-up	107	49 (45.8)	58 (54.2)	1.21, 0.73 to 2.02	.459
Aware of the main story only	655	280 (42.7)	375 (57.3)	1.17, 0.88 to 1.57	.283
vs. Unaware of the story	574	288 (50.2)	286 (49.8)		
**Recruitment wave**					
Wave 2: 20-26th June	557	255 (45.8)	663 (55.8)	0.81, 0.64 to 1.04	.097
vs. Wave 1: 6-10th June	1,189	526 (44.2)	302 (54.2)		
**Gender**					
Male	754	317 (42.0)	437 (58.0)	1.15, 0.86 to 1.54	.334
vs. Female	992	464 (46.8)	528 (53.2)		
**Ethnicity**					
White British	1,450	614 (42.3)	836 (57.7)	1.17, 0.85 to 1.61	.328
vs. Other groups	296	167 (54.4)	129 (43.6)		
**Marital status**					Overall: .504
Married/Living as a couple	964	422 (42.8)	542 (56.2)	1.04, 0.77 to 1.42	.782
Widowed/Divorced/Separated	341	139 (40.8)	202 (59.2)	0.86, 0.57 to 1.29	.460
vs. Single	441	220 (49.9)	221 (50.1)		
**Highest level of education**					Overall: .056
Graduate level/Above	494	230 (46.6)	264 (53.4)	0.64, 0.42 to 0.98	.042
A-levels/AS levels/Equivalents	438	201 (45.9)	237 (54.1)	0.70, 0.46 to 1.06	.089
GCSEs/Equivalents	429	213 (49.7)	216 (50.3)	0.63, 0.43 to 0.94	.022
Trade apprenticeships/Other	86	41 (47.7)	45 (52.3)	0.44, 0.25 to 0.80	.007
vs. No formal qualifications	299	96 (32.1)	203 (67.9)		
**Social class grade**					Overall: .711
Grade A or B	317	128 (40.4)	189 (59.6)	1.20, 0.82 to 1.76	.342
Grade C1	505	231 (45.7)	274 (54.3)	1.02, 0.74 to 1.39	.923
Grade C2	385	181 (47.0)	204 (53.0)	0.97, 0.70 to 1.36	.874
vs. Grade D or E	539	241 (44.7)	298 (55.3)		
**Employment status**					
Working	806	411 (51.0)	395 (49.0)	0.82, 0.63 to 1.07	.135
vs. Not working	940	370 (39.4)	570 (60.6)		
**Area type**					
Urban	1,420	635 (44.7)	785 (55.3)	1.12, 0.84 to 1.50	.430
vs. Rural	326	146 (44.8)	180 (55.2)		
**Personal diagnosis of cancer**					
Yes	1,599	718 (44.9)	881 (55.1)	0.84, 055 to 1.28	.404
vs. No	147	63 (42.9)	84 (57.1)		
**Personal experience of breast screening**					Overall: .284
Taken part	411	159 (38.7)	252 (61.3)	1.33, 0.91 to 1.95	.145
Invited, never taken part	48	21 (43.8)	27 (56.3)	0.95, 0.46 to 1.98	.894
vs. Not eligible or not invited	1,287	601 (46.7)	686 (53.3)		
**Belief that screening is almost always a good idea**					
Yes	1,609	701 (43.6)	908 (56.4)	1.30, 0.85 to 1.97	.230
vs. No or not sure	137	80 (58.4)	57 (41.6)		
**Awareness of volcano news**					
Yes	1,332	565 (42.4)	767 (57.6)	1.04, 0.77 to 1.40	.789
vs. No or not sure	414	216 (52.2)	198 (47.8)		
**Awareness of election news**					
Yes	1,114	467 (41.9)	647 (58.1)	1.19, 0.93 to 1.54	.172

For peer review only

vs. No or not sure	632	314 (49.7)	318 (50.3)		
**General level of trust in NHS**					Overall: **<.0005**
A lot	943	202 (21.4	741 (78.6)	13.53, 6.65 to 27.54	**<.0005**
Somewhat	589	409 (69.4)	180 (30.6)	1.62, 0.80 to 3.31	.183
A little	163	130 (79.8)	33 (20.2)	1.00, 0.46 to 2.21	.994
vs. Not at all	51	40 (78.4)	11 (21.6)		
**Age (in years)**	1,746	46.9 (19.1)	53.9 (21.0)	1.01, 1.00 to 1.02	.087

Table E – Full results of the binary logistic regression model testing for an association between awareness of the breast screening media coverage and trust in the NHS

		**A lot vs. Not at all; a little; somewhat: n (%)**		**Adjusted OR, 95% CI**	**p-value**
**Characteristic**	**Total (n=1,746)**	**Less than a lot (n=803; 46.0%)**	**A lot (n=943; 54.0%)**	**A lot (vs. Less than a lot)**	
**Screening story awareness**					Overall: .290
Aware of the main story and both follow-up commentaries	238	102 (42.9)	136 (57.1)	0.87, 0.59 to 1.30	.503
Aware of the main story and overdiagnosis follow-up	172	76 (44.2)	96 (55.8)	0.78, 0.51 to 1.21	.267
Aware of the main story and all-cause mortality follow-up	107	57 (53.3)	50 (46.7)	0.58, 0.35 to 0.97	.039
Aware of the main story only	655	299 (45.6)	356 (54.4)	0.81, 0.60 to 1.09	.160
vs. Unaware of the story	574	269 (46.9)	305 (53.1)		
**Recruitment wave**					
Wave 2: 20-26th June	557	248 (44.5)	309 (55.5)	1.21, 0.95 to 1.55	.118
vs. Wave 1: 6-10th June	1,189	555 (46.7)	634 (53.3)		
**Age**					Overall: .052
65+	530	198 (37.4)	332 (62.6)	1.04, 0.61 to 1.79	.880
55-64	245	111 (45.3)	134 (54.7)	0.76, 0.44 to 1.30	.309
45-54	235	115 (48.9)	120 (51.1)	0.83, 0.50 to 1.39	.484
35-44	245	135 (55.1)	110 (44.9)	0.59, 0.36 to 0.96	.035
25-34	265	152 (57.4)	113 (42.6)	0.56, 0.35 to 0.91	.018
vs. 16-24	226	92 (40.7)	134 (59.3)		
**Gender**					
Male	754	319 (42.3)	435 (57.7)	0.99, 0.73 to 1.33	.985
vs. Female	992	484 (48.8)	508 (51.2)		
**Ethnicity**					
White British	1,450	634 (43.7)	816 (56.3)	1.47, 1.07 to 2.02	**.019**
vs. Other groups	296	169 (57.1)	127 (42.9)		
**Marital status**					Overall: .870
Married/Living as a couple	964	440 (45.6)	524 (54.4)	1.07, 0.78 to 1.47	.685
Widowed/Divorced/Separated	341	151 (44.3)	190 (55.7)	1.00, 0.66 to 1.50	.990
vs. Single	441	212 (48.1)	229 (51.9)		
**Highest level of education**					Overall: .076
Graduate level/Above	494	233 (47.2)	261 (52.8)	1.20, 0.79 to 1.83	.386
A-levels/AS levels/Equivalents	438	199 (45.4)	239 (54.6)	1.12, 0.75 to 1.67	.582
GCSEs/Equivalents	429	224 (52.2)	205 (47.8)	0.86, 0.59 to 1.26	.447
Trade apprenticeships/Other	86	32 (37.2)	54 (62.8)	1.85, 1.01 to 3.39	.047
vs. No formal qualifications	299	115 (38.5)	184 (61.5)		
**Social class grade**					Overall: .990
Grade A or B	317	140 (44.2)	177 (55.8)	0.96, 0.65 to 1.41	.828
Grade C1	505	232 (45.9)	273 (54.1)	0.99, 0.72 to 1.37	.968
Grade C2	385	179 (46.5)	206 (53.5)	1.02, 0.73 to 1.43	.892
vs. Grade D or E	539	252 (46.8)	206 (53.5)		
**Employment status**					
Working	806	410 (50.9)	396 (49.1)	0.94, 0.71 to 1.25	.673
vs. Not working	940	393 (41.8)	547 (58.2)		
**Area type**					
Urban	1,420	656 (46.2)	764 (53.8)	0.96, 0.72 to 1.29	.795
vs. Rural	326	147 (45.1)	179 (54.9)		

For peer review only

**Personal diagnosis of cancer**					
Yes	1,599	737 (46.1)	862 (53.9)	1.00, 0.66 to 1.50	.994
vs. No	147	66 (44.9)	81 (55.1)		
**Personal experience of breast screening**					Overall: .062
Taken part	411	193 (47.0)	218 (53.0)	0.65, 0.43 to 0.97	.035
Invited, never taken part	48	20 (41.7)	28 (58.3)	1.10, 0.51 to 2.35	.813
vs. Not eligible or not invited	1,287	590 (45.8)	697 (54.2)		
**Belief that screening is almost always a good idea**					
Yes	1,609	715 (44.4)	894 (55.6)	1.96, 1.28 to 3.00	**.002**
vs. No or not sure	137	88 (64.2)	49 (35.8)		
**Awareness of volcano news**					
Yes	1,332	594 (44.6)	738 (55.4)	1.00, 0.74 to 1.35	.987
vs. No or not sure	414	209 (50.5)	205 (49.5)		
**Awareness of election news**					
Yes	1,114	491 (44.1)	623 (55.9)	1.03, 0.80 1.33	.817
vs. No or not sure	632	312 (49.4)	320 (50.6)		
**General level of trust in GP**					Overall: **<.0005**
A lot	965	224 (23.2)	741 (76.8)	11.98, 6.07 to 23.64	**<.0005**
Somewhat	513	370 (72.1)	143 (27.9)	1.39, 0.70 to 2.76	.350
A little	212	165 (77.8)	47 (22.2)	1.12, 0.54 to 2.33	.770
vs. Not at all	56	44 (78.6)	12 (21.4)		

For peer review only

Table F – Full results of the ordinal logistic regression model testing for an association between awareness of the breast screening media coverage and frequency of breast cancer worry

Characteristic	Total (n=700)	Frequency of worry about breast cancer: n (%)/M (SD) Never (n=210; 30.0%)	Occasionally (n=231; 33.0%)	Sometimes (n=177; 25.3%)	Often (n=43; 6.1%)	Very often (n=39; 5.6%)	Adjusted OR, 95% CI More frequent worry about breast cancer	p-value
**Screening story awareness**								Overall: .084
Aware of the main story and both follow-up commentaries	88	32 (36.4)	33 (37.5)	15 (17.0)	2 (2.3)	6 (6.8)	0.94, 0.56 to 1.56	.797
Aware of the main story and overdiagnosis follow-up	63	22 (34.9)	20 (31.7)	16 (25.4)	2 (3.2)	3 (4.8)	0.97, 0.56 to 1.68	.917
Aware of the main story and all-cause mortality follow-up	36	10 (27.8)	15 (41.7)	6 (16.7)	2 (5.6)	3 (8.3)	1.40, 0.71 to 2.78	.329
Aware of the main story only	270	64 (23.7)	89 (33.0)	74 (27.4)	23 (8.5)	20 (7.4)	1.51, 1.05 to 2.16	.025
vs. Unaware of the story	243	82 (33.7)	74 (30.5)	66 (27.2)	14 (5.8)	7 (2.9)		
**Recruitment wave**								
Wave 2: 20-26th June	229	81 (35.4)	71 (31.0)	52 (22.7)	11 (4.8)	14 (6.1)	0.81, 0.60 to 1.10	.174
vs. Wave 1: 6-10th June	471	129 (27.4)	160 (34.0)	125 (26.5)	32 (6.8)	25 (5.3)		
**Ethnicity**								
White British	563	160 (28.4)	194 (34.5)	140 (24.9)	35 (6.2)	34 (6.0)	1.06, 0.73 to 1.54	.766
vs. Other groups	137	50 (36.5)	37 (27.0)	37 (27.0)	8 (5.8)	5 (3.6)		
**Marital status**								Overall: .138
Married/Living as a couple	403	106 (26.3)	149 (37.0)	99 (24.6)	26 (6.5)	23 (5.7)	1.40, 0.99 to 1.98	.060
Widowed/Divorced/Separated	100	34 (34.0)	24 (24.0)	29 (29.0)	5 (5.0)	8 (8.0)	1.48, 0.90 to 2.44	.123
vs. Single	197	70 (35.5)	58 (29.4)	49 (24.9)	12 (6.1)	8 (4.1)		
**Highest level of education**								Overall: **.017**
Graduate level/Above	230	65 (28.3)	84 (36.5)	62 (27.0)	11 (4.8)	8 (3.5)	0.43, 0.24 to 0.76	**.004**
A-levels/AS levels/Equivalents	206	67 (32.5)	71 (34.5)	43 (20.9)	14 (6.8)	11 (5.3)	0.40, 0.23 to 0.70	**.001**
GCSEs/Equivalents	176	51 (29.0)	54 (30.7)	44 (25.0)	15 (8.5)	12 (6.8)	0.58, 0.34 to 0.99	**.045**
Trade apprenticeships/Other	17	6 (35.3)	6 (35.3)	5 (29.4)	0 (0.0)	0 (0.0)	0.42, 0.15 to 1.14	.087
vs. No formal qualifications	71	21 (29.6)	16 (22.5)	23 (32.4)	3 (4.2)	8 (11.3)		
**Social class grade**								Overall: .206
Grade A or B	125	28 (22.4)	56 (44.8)	30 (24.0)	3 (2.4)	8 (6.4)	1.44, 0.89 to 2.33	.141
Grade C1	209	52 (24.9)	71 (34.0)	59 (28.2)	18 (8.6)	9 (4.3)	1.46, 0.97 to 2.22	.073
Grade C2	165	57 (34.5)	48 (29.1)	38 (23.0)	14 (8.5)	8 (4.8)	1.08, 0.70 to 1.64	.740
vs. Grade D or E	201	73 (36.3)	56 (27.9)	50 (24.9)	8 (4.0)	14 (7.0)		
**Employment status**								
Working	392	106 (27.0)	133 (33.9)	110 (28.1)	27 (6.9)	16 (4.1)	1.03, 0.76 to 1.39	.856
vs. Not working	308	104 (33.8)	98 (31.8)	67 (21.8)	16 (5.2)	23 (7.5)		
**Area type**								
Urban	574	179 (31.2)	187 (32.6)	142 (24.7)	34 (5.9)	32 (5.6)	0.79, 0.55 to 1.13	.200

For peer review only

vs. Rural	126	31 (24.6)	16 (36.4)	8 (18.2)	3 (6.8)	7 (15.9)		
**Personal diagnosis of cancer**								
Yes	44	10 (22.7)	16 (36.4)	8 (18.2)	3 (6.8)	7 (15.9)	2.13, 1.18 to 3.83	**.012**
vs. No	656	200 (30.5)	215 (32.8)	169 (25.8)	40 (6.1)	32 (4.9)		
**Personal experience of breast screening**								Overall: .629
Taken part	221	77 (34.8)	74 (33.5)	45 (20.4)	8 (3.6)	17 (7.7)	0.77, 0.46 to 1.31	.342
Invited, never taken part	34	12 (35.3)	11 (32.4)	7 (20.6)	2 (5.9)	2 (5.9)	0.87, 0.94 to 1.90	.722
vs. Not eligible or not invited	445	121 (27.2)	146 (32.8)	125 (28.1)	33 (7.4)	20 (4.5)		
**Belief that screening is almost always a good idea**								
Yes	660	192 (29.1)	220 (33.3)	167 (25.3)	42 (6.4)	39 (5.9)	1.28, 0.65 to 2.49	.478
vs. No or not sure	40	18 (45.0)	11 (27.5)	10 (25.0)	1 (2.5)	0 (0.0)		
**Awareness of volcano news**								
Yes	505	148 (29.3)	175 (34.7)	122 (24.2)	30 (5.9)	30 (5.9)	0.94, 0.66 to 1.34	.739
vs. No or not sure	195	62 (31.8)	56 (28.7)	55 (28.2)	13 (6.7)	9 (4.6)		
**Awareness of election news**								
Yes	424	130 (30.7)	145 (34.2)	101 (23.8)	21 (5.0)	27 (6.4)	0.86, 0.63 to 1.16	.320
vs. No or not sure	276	80 (29.0)	86 (31.2)	76 (27.5)	22 (8.0)	12 (4.3)		
**General level of trust in GP**								Overall: .618
A lot	339	105 (31.0)	115 (33.9)	81 (23.9)	19 (5.6)	19 (5.6)	1.12, 0.52 to 2.42	.771
Somewhat	221	55 (24.9)	76 (34.4)	61 (27.6)	19 (5.6)	10 (4.5)	1.35, 0.62 to 2.94	.443
A little	109	37 (33.9)	32 (29.4)	31 (28.4)	3 (2.8)	6 (5.5)	1.04, 0.47 to 2.29	.923
vs. Not at all	31	13 (41.9)	8 (25.8)	4 (12.9)	2 (6.5)	4 (12.9)		
**General level of trust in NHS**								Overall: .209
A lot	344	106 (30.8)	117 (34.0)	78 (22.7)	24 (7.0)	19 (5.5)	2.16, 0.88 to 5.27	.092
Somewhat	257	65 (25.3)	86 (33.5)	75 (29.2)	18 (7.0)	13 (5.1)	2.55, 1.04 to 6.25	.040
A little	76	25 (32.9)	26 (34.2)	19 (25.0)	0 (0.0)	6 (7.9)	2.26, 0.88 to 5.79	.090
vs. Not at all	23	14 (60.9)	2 (8.7)	5 (21.7)	1 (4.3)	1 (4.3)		
**Future breast screening intentions**								Overall: **.005**
Yes, definitely	601	165 (27.5)	204 (33.9)	157 (26.1)	39 (6.5)	36 (6.0)	3.32, 1.19 to 9.27	**.022**
Yes, probably	70	25 (35.7)	24 (34.3)	15 (21.4)	3 (4.3)	3 (4.3)	2.18, 0.72 to 6.54	.166
No, probably not	13	10 (76.9)	2 (15.4)	0 (0.0)	1 (7.7)	0 (0.0)	0.56, 0.11 to 2.97	.499
vs. No, definitely not	16	10 (62.5)	1 (6.3)	5 (31.3)	0 (0.0)	0 (0.0)		
**Age (in years)**	700	44.8 (16.0)	42.6 (15.0)	40.9 (14.1)	39.1 (13.0)	48.6 (14.7)	0.99, 0.97 to 1.01	.159

For peer review only

Table G – Full results of the binary logistic regression model testing for an association between awareness of the breast screening media coverage and breast screening intentions

		Yes, definitely vs. Yes, probably; no, probably not; no, definitely not: n (%)		Adjusted OR, 95% CI	p-value
Characteristic	Total (n=700)	No definite intention (n=99; 14.1%)	Definite intention (n=601; 85.9%)	Definite intention (vs. No definite intention)	
**Screening story awareness**					Overall: .108
Aware of the main story and both follow-up commentaries	88	10 (11.4)	78 (88.6)	2.01, 0.74 to 5.48	.172
Aware of the main story and overdiagnosis follow-up	63	4 (4.3)	59 (93.7)	2.66, 0.79 to 8.89	.113
Aware of the main story and all-cause mortality follow-up	36	6 (16.7)	30 (83.3)	0.66, 0.20 to 2.13	.486
Aware of the main story only	270	22 (8.1)	248 (91.9)	1.88, 0.99 to 3.57	.054
vs. Unaware of the story	243	57 (23.5)	186 (76.5)		
**Recruitment wave**					
Wave 2: 20-26th June	229	40 (17.5)	189 (82.5)	0.71, 0.42 to 1.21	.211
vs. Wave 1: 6-10th June	471	59 (12.5)	412 (87.5)		
**Age**					Overall: .050
65+	69	7 (10.1)	62 (89.9)	0.84, 0.13 to 5.37	.855
55-64	123	5 (4.1)	118 (95.9)	2.49, 0.42 to 14.74	.313
45-54	128	10 (7.8)	118 (92.2)	3.59, 1.10 to 11.69	.034
35-44	143	21 (14.7)	122 (85.3)	2.72, 1.16 to 6.41	.022
25-34	144	29 (20.1)	115 (79.9)	2.82, 1.24 to 6.42	.014
vs. 16-24	93	27 (29.0)	66 (71.0)		
**Ethnicity**					
White British	563	72 (12.8)	491 (87.2)	0.96, 0.51 to 1.83	.905
vs. Other groups	137	27 (19.7)	110 (80.3)		
**Marital status**					Overall: .321
Married/Living as a couple	403	49 (12.2)	354 (87.8)	0.99, 0.53 to 1.84	.970
Widowed/Divorced/Separated	100	6 (6.0)	94 (94.0)	2.18, 0.73 to 6.53	.163
vs. Single	197	44 (22.3)	153 (77.7)		
**Highest level of education**					Overall: .169
Graduate level/Above	230	35 (15.2)	195 (84.8)	0.69, 0.22 to 2.20	.533
A-levels/AS levels/Equivalents	206	35 (17.0)	171 (83.0)	0.87, 0.29 to 2.62	.808
GCSEs/Equivalents	176	19 (10.8)	157 (89.2)	1.92, 0.62 to 5.92	.259
Trade apprenticeships/Other	17	3 (17.6)	14 (82.4)	1.38, 0.24 to 0.81	.720
vs. No formal qualifications	71	7 (9.9)	64 (90.1)		
**Social class grade**					Overall: .186
Grade A or B	125	14 (11.2)	111 (88.8)	1.87, 0.76 to 4.61	.177
Grade C1	209	25 (12.0)	184 (88.0)	2.36, 1.08 to 5.16	.032
Grade C2	165	24 (14.5)	141 (85.5)	1.85, 0.86 to 3.98	.118
vs. Grade D or E	201	36 (17.9)	165 (82.1)		
**Employment status**					
Working	392	56 (14.3)	336 (85.7)	0.70, 0.39 to 1.26	.238
vs. Not working	308	43 (14.0)	265 (86.0)		
**Area type**					
Urban	574	83 (14.5)	491 (85.5)	0.79, 0.39 to 1.59	.512
vs. Rural	126	16 (12.7)	110 (87.3)		
**Personal diagnosis of cancer**					
Yes	44	7 (15.9)	37 (84.1)	0.94, 0.30 to 2.98	.918
vs. No	656	92 (14.0)	564 (86.0)		
**Personal experience of breast screening**					Overall: **.013**
Taken part	221	9 (4.1)	212 (95.9)	6.12, 1.37 to 27.33	**.018**
Invited, never taken part	34	6 (17.6)	28 (82.4)	0.99, 0.21 to 4.61	.986
vs. Not eligible or not invited	445	84 (18.9)	361 (81.1)		
**Belief that screening is almost always a good idea**					
Yes	660	81 (12.3)	579 (87.7)	9.08, 3.77 to 21.88	**<.0005**
vs. No or not sure	40	18 (45.0)	22 (55.0)		

For peer review only

**Awareness of volcano news**					
Yes	505	54 (10.7)	451 (89.3)	1.34, 0.74 to 2.41	.335
vs. No or not sure	195	45 (23.1)	150 (76.9)		
**Awareness of election news**					
Yes	424	47 (11.1)	377 (88.9)	1.42, 0.82 to 2.46	.208
vs. No or not sure	276	52 (18.8)	224 (81.2)		
**General level of trust in GP**					Overall: .025
A lot	339	31 (9.1)	308 (90.9)	1.55, 0.45 to 5.29	.487
Somewhat	221	44 (19.9)	177 (80.1)	0.76, 0.23 to 5.67	.663
A little	109	16 (14.7)	93 (85.3)	2.48, 0.69 to 8.90	.163
vs. Not at all	31	8 (25.8)	23 (74.2)		
**General level of trust in NHS**					Overall: .007
A lot	344	34 (9.9)	310 (90.1)	1.16, 0.29 to 4.64	.832
Somewhat	257	42 (16.3)	215 (83.7)	0.70, 0.18 to 2.79	.614
A little	76	18 (23.7)	58 (76.3)	0.27, 0.06 to 1.11	.068
vs. Not at all	23	5 (21.7)	18 (78.3)		
**Frequency of breast cancer worry**					Overall: **.028**
Very often	39	3 (7.7)	36 (92.3)	3.00, 0.72 to 12.51	.132
Often	43	5 (9.3)	39 (90.7)	2.95, 0.85 to 10.26	.089
Sometimes	177	20 (11.3)	157 (88.7)	2.59, 1.31 to 5.15	**.006**
Occasionally	231	27 (11.7)	204 (88.3)	2.15, 1.15 to 4.02	**.016**
vs. Never	210	45 (21.4)	165 (78.6)		

For peer review only

BMJ Open

Public awareness of and responses to media coverage of invitation errors in the Breast Screening Programme in England: A cross-sectional population survey

Journal:	*BMJ Open*
Manuscript ID	bmjopen-2018-028040.R1
Article Type:	Research
Date Submitted by the Author:	21-May-2019

Complete List of Authors:	Ghanouni, Alex; University College London, Research Department of Behavioural Science and Health<br>von Wagner, Christian; UCL, Research Department of Behavioural Science and Health<br>Waller, Jo; UCL, Research Department of Behavioural Science and Health

**Primary Subject Heading**:	Public health
Secondary Subject Heading:	Communication
Keywords:	Organisation of health services < HEALTH SERVICES ADMINISTRATION & MANAGEMENT, JOURNALISM (see Medical Journalism), PUBLIC HEALTH, Breast imaging < RADIOLOGY & IMAGING

SCHOLARONE™
Manuscripts

FULL TITLE PAGE

Title: Public awareness of and responses to media coverage of invitation errors in the Breast Screening Programme in England: A cross-sectional population survey

Manuscript type: Research article

Authors:

Alex Ghanouni[a] a.ghanouni@ucl.ac.uk

Christian von Wagner[a] c.wagner@ucl.ac.uk

Jo Waller[ab] j.waller@ucl.ac.uk | Tel: +44 (0)20 7679 5958 | Fax: +44 (0)20 7679 8354

Affiliations:

[a]Research Department of Behavioural Science and Health, University College London, Gower Street, London, WC1E 6BT, United Kingdom

[b]Corresponding author

For peer review only - http://bmjopen.bmj.com/site/about/guidelines.xhtml

For peer review only

ABSTRACT

Objectives: In May 2018, the British Health Secretary announced the 'serious failure' that 450,000 women had missed out on invitations to breast screening in England, leading to extensive media coverage. This study measured public awareness of the story and tested for associated factors (e.g. educational level and trust in the NHS).

Design: A computer-assisted face-to-face survey in June 2018.

Setting: Participants completed the survey in their homes.

Participants: Males and females aged 16 years or older in England.

Primary and secondary outcome measures: Awareness of aspects of the media coverage and reported statistics. Other data included demographics (e.g. ethnicity), awareness of unrelated contemporaneous news stories, trust in participants' GPs and the NHS, and (among women) worry about breast cancer and future breast screening intentions.

Results: Descriptive statistics showed that 66.7% of 1,894 participants reported being aware of the media coverage. Regression analyses showed that those who were aware of other news stories, were white British, and had a higher level of education or social class grade were more likely to be aware. In contrast, only 36.0% correctly identified at least one of two headline statistics. This study did not find evidence that awareness was negatively associated with trust in participants' GPs or the NHS, breast cancer worry or future breast cancer screening intentions.

Conclusions: Awareness of the breast screening news story was high but recall of reported statistics was much lower: the public may have retained only the gist of quantitative information. Associations between story awareness and attitudes or behaviour were not apparent.

Keywords: Breast imaging; Journalism; Organisation of health services; Public Health

For peer review only

Strengths and limitations of this study:

- This study builds on previous research on media coverage around public health concerns by measuring levels of awareness among the general public and testing for characteristics associated with awareness.
- The survey was carried out shortly after media coverage of the announcement began, when awareness and knowledge were likely to be at their highest.
- Associations between awareness of media coverage and e.g. greater worry about breast cancer and lower trust in the NHS were not apparent but Type II error cannot be excluded.
- Tests for associations between awareness of media coverage and screening behaviour were based on intended future uptake; actual uptake may differ.

For peer review only

INTRODUCTION

On 2nd May 2018, the Health Secretary in Great Britain, Jeremy Hunt, made an unanticipated statement to the House of Commons regarding *"a serious failure…in the national Breast Screening Programme"*. Mr Hunt stated that since 2009, *"a computer algorithm failure"* had resulted in approximately 450,000 women not being invited to their final regular breast screening appointment (i.e. when they were aged 68 to 71 years). He indicated to the House that *"[the] current best estimate based on statistical modelling…is that there may be between 135 and 270 women who had their lives shortened as a result"* and that women affected *"will automatically be sent an invitation to a catch-up screening"*.[1] News of this statement was reported extensively in the national media (e.g. [2-4]) and prompted a volume of follow-up commentary from academics aiming to add context to this story. For example, some raised arguments that breast screening has no effect on all-cause mortality and risks resulting in overdiagnosis.[5-6]

Awareness of health-related media coverage is likely to be very high among academics and clinicians who are professionally invested in the topic. However, research is lacking on the prevalence of awareness of this type of news among the general public. In the absence of empirical data, it might be hypothesised to be either high (e.g. because mainstream media coverage has an extremely wide reach) or generally low (because members of the public are more focused on their personal priorities or do not have a specific interest in health news).

For peer review only - http://bmjopen.bmj.com/site/about/guidelines.xhtml

Levels of public awareness of health media coverage is significant because it represents the proportion of people who may be influenced by it: previous research has found that media coverage of cancer-related stories in the United Kingdom has appreciable public health implications. For example, there is evidence that the cervical cancer diagnosis and death of a young female celebrity, Jade Goody, influenced women's cervical cancer screening decisions and temporarily increased uptake and diagnoses of high-grade cervical neoplasia.[7-10] Similarly, uptake of the colorectal screening programme increased following coverage of the United Kingdom Flexible Sigmoidoscopy Screening Trial.[11-12] Comparable findings have been reported by studies of pre-planned media messages such as Public Health England's 'Be Clear on Cancer' campaigns, which aim to increase cancer symptom awareness. These were associated with an increase in symptomatic attendance at General Practices and referrals to secondary care.[13-15]

In these cases, media coverage was associated with an increase in healthcare usage. However, news about an error in the screening programme may have had adverse effects, such as diminishing trust in the National Health Service (with corresponding negative implications for help-seeking), more frequent worry about breast cancer, and being less inclined to have breast screening in future. To our knowledge, this possibility has not been investigated by research to date.

This study surveyed awareness of the coverage shortly after the announcement (when conscious recall was likely to be highest) in a large, sociodemographically diverse sample of the general public. In order to make a more complete assessment of this awareness, we also measured knowledge of the relevant statistics most commonly reported as part of the story (i.e. the number of women estimated to have missed an invitation and to have had their lives shortened) since these were a key factor in making a personal assessment of the scale and severity of the invitation errors. We also recognised that people's concerns about the initial coverage may have been moderated by follow-up commentary noting issues around overdiagnosis and all-cause mortality in breast screening. We used these measures to conduct an exploratory analysis of variables associated with awareness of the media coverage, including education, gender, and awareness of other news stories that were reported around the same time. We also tested the hypotheses that awareness of the breast screening media coverage would be associated with lower trust in participants' GPs and the NHS and

For peer review only - http://bmjopen.bmj.com/site/about/guidelines.xhtml

(in women) more frequent worry about breast cancer and being less likely to intend to participate in breast screening in future.

METHODS

Design

Institutional ethical approval was obtained (registration number: 2951/006). A market research agency (Kantar TNS UK) collected data in two waves of sampling between 6th and 10th June 2018 (i.e. less than six weeks after the initial news story. The survey questions formed one module within a weekly face-to-face computer-assisted omnibus survey on a wider range of topics. Random location sampling was used to identify target households based on the 2011 Census and Postcode Address File. At each location, quotas were set with the aim of achieving national representativeness based on working status, children in the household, gender, and age.

The full survey is included in Appendix 1. Participants were initially shown a computer screen with text introducing the study and asking for their consent to participate. They were also given an information card containing debrief text and directions to further information about breast screening.

Participants

Eligible participants were all males and females in England aged 16 years or older who consented to take part in this module of the survey. The sample includes women eligible for breast screening (i.e. aged 47 to 73 years) and also members of the general population (males and females aged 16 years or older) since it was hypothesised that awareness of the story had the potential to negatively affect perceptions of other health services, irrespective of whether participants were affected directly. Sample size was based on budgetary constraints and the number of participants who could be approached no more than six weeks after the initial news story.

Patient and public involvement

Since the results of the study were expected to be highly time-sensitive, rapid data collection was prioritised over involving patients and the public in the design and conduct of the study. In order to minimise data protection issues, survey responses were received by the research team in anonymised format, meaning that it is not possible to disseminate study results to participants.

For peer review only - http://bmjopen.bmj.com/site/about/guidelines.xhtml

Measures

Demographics:

General background information included participants' self-reported age (in years), gender, ethnic origin, marital status, education, social class grade,[16] employment status, and urban or rural area type.

Cancer and breast screening experience, and attitudes towards screening:

Participants were asked whether they had been diagnosed with any of several types of cancer themselves. Women aged 47 years or older were also asked if they had ever been i) invited to and ii) participated in the Breast Screening Programme.

Participants were asked about their attitudes towards screening via a previously used question,[17] *"routine screening means testing healthy people to find cancer before they have any symptoms. Do you think routine cancer screening tests for healthy people are almost always a good idea?"*. Response options were *"yes"*, *"no"*, and *"not sure"*.

Awareness of the breast screening news story:

Participants were asked to read a brief summary of the story (see Appendix 1, Q7), the main details of which were derived from the primary story on the topic on the BBC news website.[4] This was followed by the question, *"do you recall seeing or hearing anything about this news story before now?"*. Response options were *"yes"*, *"no"*, and *"not sure"*.

It was anticipated that directions of associations with awareness may depend on the specific parts of the story of which participants were aware. Consequently, participants who reported being aware of the main news story were also asked about their awareness of issues relating to all-cause mortality and overdiagnosis using two further summaries (see Appendix 1, Q14 and Q15), derived from two sources.[5-6]

Questions for assessing awareness were the same as previous. Participants reporting awareness of the news story were also asked where they saw or heard it and whether they discussed or shared it

For peer review only - http://bmjopen.bmj.com/site/about/guidelines.xhtml

with anyone else. They were also asked two questions on the key statistics reported based on the following summaries:

"The Health Secretary, Jeremy Hunt, gave an estimate of the number of women who had failed to get invitations since 2009."

"The Health Secretary also gave an estimate, based on computer modelling, of the number of women who may have had their lives shortened."

For both, the question was *"which of the following do you think is the estimate that he gave?"*. For the first question, response options consisted of the true estimate (450,000) and three alternatives that were orders of magnitude higher or lower (4,500, 45,000, and 4,500,000). Similarly, response options for the second question consisted of the correct answer (between 135 and 270) and alternatives that were either an order of magnitude higher (1,350 and 2,700), lower (13 and 27), or both higher and lower (13 and 2,700). Response order was presented in one of two different ways for each participant (determined at random) to reduce potential order effects.

Awareness of news stories unrelated to breast screening:

Awareness of other news stories was measured by asking participants to read two further summaries (one on a volcano eruption in Hawaii; one on local council elections in England; see Appendix 1, Q19 and Q20). This was followed by the same measure of awareness as in previous questions. Main details were derived from the primary stories on the BBC news website.[18-19] These two stories were selected for comparison because they were reported around the same time and also consisted of specific, definable events.

Trust in health services:

Participants were asked two questions based on previously used items,[20-21] *"in general, how much do you trust…"* i) *"…your general practitioner?"* and ii) *"…the NHS?"*. Response options for both were *"not at all"*, *"a little"*, *"somewhat"*, *"a lot"*, and *"not sure"*.

Frequency of breast cancer worry:

For peer review only - http://bmjopen.bmj.com/site/about/guidelines.xhtml

Breast cancer worry (among women) was measured using an item based on one previously used,[22] *"how often do you worry about your chances of getting breast cancer yourself?"*. Response options were, *"never"*, *"occasionally"*, *"sometimes"*, *"often"*, *"very often"*, *"not sure"*, and *"prefer not to say"*.

Breast screening intentions:

Women aged 16 to 69 years were asked, *"do you think you will go for breast screening when you are next offered it?"*. Response options were *"yes, definitely"*, *"yes, probably"*, *"no, probably not"*, and *"no, definitely not"*.

Analysis

Participant characteristics and awareness about the news stories are reported using descriptive statistics. Responses of *"prefer not to say"* were excluded, as were responses of *"not sure"* for ordinal variables. Other responses of *"not sure"*, were grouped with *"no"*. Ethnicity was dichotomised into *"white British"* and *"other groups"*; social class grades were grouped into *"A or B"*, *"C1"*, *"C2"*, and *"D or E"*. For education, *"trade apprenticeships"* were grouped with *"other qualifications"*. Responses to measures of invitations to and participation in breast screening were coded into *"not eligible or not invited"*, *"invited, never taken part"*, and *"taken part"*.

One exploratory regression model tested for variables potentially associated with whether people responded to the survey. Three exploratory regression models tested for variables potentially associated with i) awareness of the breast screening news; and stating correctly the number of women who were ii) not invited for screening and iii) estimated to have had their lives shortened. A further four regression models tested the null hypotheses that awareness of the breast screening news story was not associated with trust in iv) participants' GPs and v) the NHS in the whole sample; and vi) frequency of worry about breast cancer and vii) intentions to participate in breast screening in future among women aged 70 years or less, after adjusting for covariates.

For the model assessing variables associated with responding to the questionnaire, the main variables of interest were recruitment wave, gender, ethnicity, marital status, social class grade, employment status, area type, and age (since these were the variables where data were available for both participants and non-participants). For the four main exploratory models and hypothesis testing models, independent variables were as above with the addition of other available measures (listed in

For peer review only - http://bmjopen.bmj.com/site/about/guidelines.xhtml

tables) where multi-collinearity was not an appreciable issue (i.e. Variance Inflation Factors <10). Age was included in models as either a continuous variable or divided into age groups (where a Box-Tidwell procedure found evidence that the assumption of linearity was not met; p<.05). Frequency of worry about breast cancer was also included in the model of future breast screening intentions.

For models testing hypotheses, responses on measures of awareness of the breast screening story were coded into a single nominal variable with five levels: 1) *"unaware of the story"*, 2) *"aware of the main story only"*, 3) *"aware of the main story and all-cause mortality follow-up commentary"*,4) *"aware of the main story and overdiagnosis follow-up commentary"*, 5) *"aware of the main story and both follow-up commentaries"*.

Ordinal logistic regression was attempted in the first instance where dependent variables were ordinal. Tests of parallel lines suggested that the assumption of proportional odds was generally not met (p<.0005) and there were few cases in some cells. Hence, dependent variables were dichotomised and binary logistic regression was used. Participants with missing data on variables of interest were not included in models.

RESULTS

Participant characteristics

2,681 participants began the survey module. 787 (29.4%) opted out, leaving 1,894 participants who provided data. Mean age was 50.8 years (standard deviation: 20.5). Characteristics are described in Appendix 2 (Table A). Response to the survey module questions was associated with all variables in the model, except for area type (Appendix 2, Table B). Participants of the omnibus survey approached were more likely to respond to this survey module if they were invited in wave 1 (vs. wave 2), female (vs. male), white British (vs. other groups), married, living as a couple, or widowed, divorced or separated (vs. single), in higher social class grades (vs. grades D or E), working (vs. not working), and younger.

Awareness of news stories, sources of information, and variables associated with awareness of the breast screening media coverage

For peer review only - http://bmjopen.bmj.com/site/about/guidelines.xhtml

1,264/1,894 (66.7%) reported being aware of the main news story (Appendix 2, Table A) and relatively few reported being aware of follow-up commentaries: 438/1,264 (34.7%) and 367/1,264 (29.0%) recognised the commentaries on all-cause mortality and overdiagnosis, respectively. 250/1,264 (19.8%) were aware of both. 971/1,264 (76.8%) and 271/1,264 (21.4%) encountered the story on television and radio, respectively (participants could select more than one). 169/1,264 (13.4%) and 134/1,264 (10.6%) encountered the story in print newspapers and online news websites (Appendix 2, Table C). Other news sources were used relatively rarely e.g. 68/1,264 (5.4%) heard the story from social media websites. 450/1,264 (35.6%) reported discussing or sharing the story with someone else.

Participants were more likely to be aware of the story if they were aware of either of the other two news stories. Awareness of the three stories was highly interrelated: 824/1,894 participants (43.5%) were aware of all three news stories and a further 196/1,894 (10.3%) reported not being aware of any. Only 323/1,894 (17.1%) were aware of just one of the three stories and only 106/1,894 participants (5.6%) were aware of the news about breast screening, specifically. Participants were also more likely to be aware of the breast screening news story if they were white British, older, had higher levels of education or social class grade. Participants were less likely to be aware if they believed that screening was almost always a good idea. All other p-values were ≥.207 (Table 1).

Awareness of statistics from the breast screening media coverage and variables associated with awareness among participants who reported being aware of the story

Only 233 (18.4%) of the 1,264 participants who reported being aware of the story correctly recognised the number of women who had not been invited and only 268 (21.2%) correctly recognised the estimated number of women who had their lives shortened. 809 (64.0%) did not correctly identify either statistic and only 3.6% correctly identified both (Table 2). The model testing for demographic and psychological variables associated with correctly identifying either set of statistics found only weak evidence against the null hypothesis for all characteristics (p-values were ≥.087 and ≥.062 in the respective models; data not shown).

Awareness of media coverage and participants' trust their GPs and the NHS

For peer review only - http://bmjopen.bmj.com/site/about/guidelines.xhtml

For peer review only

In both these models, there was only weak evidence against the null hypothesis. Table 3 shows the main results of binary logistic regression models consisting of 1,746 participants (p=.729 and .290). Full results of the model are presented in Appendix 2 (Table D and Table E).

Awareness of media coverage and frequency of worry about breast cancer

Table 4 shows that there was only weak evidence against the null hypothesis (n=700; p=.198). Full results are included in Appendix 2 (Table F).

Awareness of media coverage and future breast screening intentions

Table 5 shows that there was only weak evidence against the null hypothesis for this analysis (n=700; p=.108). Full results are included in Appendix 2 (Table G).

Numbers of participants with missing data for each variable are shown in Appendix 2 (Table H).

For peer review only

Table 1 – Full results of the binary logistic regression model testing for variables associated with awareness of the breast screening news story

Characteristic	Total (n=1,792)	Aware vs. Not aware of the breast screening story (or not sure): n (%) Not aware/sure (n=587; 32.8%)	Aware (n=1,205; 67.2%)	Adjusted OR, 95% CI Aware of the screening story (vs. Not aware or not sure)	p-value
**Recruitment wave**					
Wave 2: 20-26th June	570	185 (32.5)	385 (67.5)	1.02, 0.79 to 1.31	.907
vs. Wave 1: 6-10th June	1,222	402 (32.9)	820 (67.1)		
**Age**					Overall: **<.0005**
65+	549	111 (20.2)	438 (79.8)	7.77, 4.52 to 13.38	**<.0005**
55-64	252	53 (21.0)	199 (79.0)	6.75, 3.92 to 11.63	**<.0005**
45-54	241	47 (19.5)	194 (80.5)	7.70, 4.56 to 13.00	**<.0005**
35-44	248	88 (35.5)	160 (64.5)	3.60, 2.22 to 5.84	**<.0005**
25-34	275	142 (51.6)	133 (48.4)	2.00, 1.27 to 3.14	**.003**
vs. 16-24	227	146 (64.3)	81 (35.7)		
**Gender**					
Male	771	234 (30.4)	537 (69.6)	1.00, 0.74 to 1.35	.999
vs. Female	1,021	353 (34.6)	668 (65.4)		
**Ethnicity**					
White British	1,491	415 (27.8)	1,076 (72.2)	3.00, 2.20 to 4.09	**<.0005**
vs. Other groups	301	172 (57.1)	129 (42.9)		
**Marital status**					Overall: .914
Married/Living as a couple	985	279 (28.3)	706 (71.7)	1.07, 0.78 to 1.47	.672
Widowed/Divorced/Separated	354	84 (23.7)	270 (76.3)	1.06, 0.70 to 1.60	.792
vs. Single	453	224 (49.4)	229 (50.6)		
**Highest level of education**					Overall: **.001**
Graduate level/Above	501	131 (26.1)	370 (73.9)	2.08, 1.34 to 3.23	**.001**
A-levels/AS levels/Equivalents	448	162 (36.2)	286 (63.8)	1.80, 1.19 to 2.73	**.006**
GCSEs/Equivalents	440	156 (35.5)	284 (64.5)	1.36, 0.92 to 2.00	.120
Trade apprenticeships/Other	89	39 (43.8)	50 (56.2)	0.75, 0.42 to 1.32	.316
vs. No formal qualifications	314	99 (31.5)	215 (68.5)		
**Social class grade**					Overall: **<.0005**
Grade A or B	326	53 (16.3)	273 (83.7)	2.44, 1.59 to 3.73	**<.0005**
Grade C1	511	165 (32.3)	346 (67.7)	1.41, 1.02 to 1.95	**.037**
Grade C2	394	142 (36.0)	252 (64.0)	1.13, 0.81 to 1.58	.469
vs. Grade D or E	561	227 (40.5)	334 (59.5)		
**Employment status**					
Working	823	287 (34.9)	536 (65.1)	0.91, 0.68 to 1.22	.909
vs. Not working	969	300 (31.0)	669 (69.0)		
**Area type**					
Urban	1,458	476 (32.6)	982 (67.4)	1.21, 0.90 to 1.64	.207
vs. Rural	334	111 (33.2)	223 (66.8)		
**Personal diagnosis of cancer**					
Yes	150	34 (22.7)	116 (77.3)	1.18, 0.74 to 1.86	.490
vs. No	1,642	553 (33.7)	1,089 (66.3)		
**Personal experience of breast screening**					Overall: .552
Taken part	425	90 (21.2)	335 (78.8)	0.92, 0.60 to 1.41	.705
Invited, never taken part	55	13 (23.6)	42 (76.4)	0.66, 0.32 to 1.39	.276
vs. Not eligible or not invited	1,312	484 (36.9)	828 (63.1)		
**Belief that screening is almost always a good idea**					
Yes	1,649	547 (33.2)	1,102 (66.8)	0.59, 0.38 to 0.94	**.025**
vs. No or not sure	143	40 (28.0)	103 (72.0)		
**Awareness of volcano news**					
Yes	1,367	325 (23.8)	1,042 (76.2)	3.14, 2.39 to 4.12	**<.0005**
vs. No or not sure	425	262 (61.6)	163 (38.4)		
**Awareness of election news**					
Yes	1,138	292 (25.7)	846 (74.3)	1.37, 1.06 to 1.75	**.014**
vs. No or not sure	654	295 (45.1)	359 (54.9)		
**General level of trust in the NHS**					Overall: .485
A lot	969	308 (31.8)	661 (68.2)	0.59, 0.29 to 1.18	.132

For peer review only - http://bmjopen.bmj.com/site/about/guidelines.xhtml

Somewhat	599	193 (32.2)	406 (67.8)	0.63, 0.31 to 1.27	.196
A little	169	69 (40.8)	100 (59.2)	0.58, 0.27 to 1.25	.166
vs. Not at all	55	17 (30.9)	38 (69.1)		

Table 2 – Descriptive statistics of participants' responses about key statistics in the breast screening media coverage; correct responses were "450,000" and "135-270"

	n (% of total; 95% CI) (n=1,264)					
**Number of women who did not**	**Number of women who may have had their life shortened. Between…**					
**receive their final invitation…**	*135 - 270*	*13 - 27*	*13 - 2,700*	*1,350 - 2,700*	*Not sure*	*Total*
450,000	46 (3.6)	6 (0.5)	79 (6.3)	71 (5.6)	31 (2.5)	233 (18.4)
4,500	68 (5.4)	20 (1.6)	28 (2.2)	22 (1.7)	30 (2.4)	168 (13.3)
45,000	130 (10.3)	22 (1.7)	76 (6.0)	86 (6.8)	54 (4.3)	368 (29.1)
4,500,000	3 (0.2)	1 (0.1)	10 (0.8)	20 (1.6)	4 (0.3)	38 (3.0)
Not sure	21 (2.1)	5 (0.4)	15 (1.2)	12 (0.9)	404 (32.0)	457 (36.2)
Total	268 (21.2)	54 (4.3)	208 (16.5)	211 (16.7)	523 (41.4)	

Table 3 – Testing for an association between awareness of the breast screening media coverage and trust in i) participants' GPs and ii) the NHS*

**General level of trust in participants' GPs**		**A lot vs. Not at all; a little; somewhat: n (%)**		**Adjusted OR, 95% CI**	**p-value**
**Characteristic**	**Total (n=1,746)**	**Less than a lot (n=781; 44.7%)**	**A lot (n=965; 55.3%)**	**A lot (vs. Less than a lot)**	
**Screening story awareness**					Overall: .729
Aware of the main story and both follow-up commentaries	238	98 (41.2)	140 (58.8)	1.10, 0.74 to 1.64	.653
Aware of the main story and overdiagnosis follow-up	172	66 (38.4)	106 (61.6)	1.31, 0.85 to 2.03	.218
Aware of the main story and all-cause mortality follow-up	107	49 (45.8)	58 (54.2)	1.21, 0.73 to 2.02	.459
Aware of the main story only	655	280 (42.7)	375 (57.3)	1.17, 0.88 to 1.57	.283
vs. Unaware of the story	574	288 (50.2)	286 (49.8)		
**General level of trust in the NHS**		**A lot vs. Not at all; a little; somewhat: n (%)**		**Adjusted OR, 95% CI**	**p-value**
**Characteristic**	**Total (n=1,746)**	**Less than a lot (n=803; 46.0%)**	**A lot (n=943; 54.0%)**	**A lot (vs. Less than a lot)**	
**Screening story awareness**					Overall: .290
Aware of the main story and both follow-up commentaries	238	102 (42.9)	136 (57.1)	0.87, 0.59 to 1.30	.503
Aware of the main story and overdiagnosis follow-up	172	76 (44.2)	96 (55.8)	0.78, 0.51 to 1.21	.267
Aware of the main story and all-cause mortality follow-up	107	57 (53.3)	50 (46.7)	0.58, 0.35 to 0.97	.039
Aware of the main story only	655	299 (45.6)	356 (54.4)	0.81, 0.60 to 1.09	.160
vs. Unaware of the story	574	269 (46.9)	305 (53.1)		

*Results are adjusted based on the following covariates: Recruitment wave, Age (Age group in the model of trust in the NHS), Gender, Ethnicity, Marital status, Highest level of education, Social class grade, Employment status, Area type, Personal diagnosis of cancer, Personal experience of breast screening, Belief that screening is almost always a good idea, Awareness of volcano news, Awareness of election news, General level of trust in the NHS (General level of trust in participants' GPs in the model of trust in the NHS). Full results of the model are reported in the Appendix

For peer review only

For peer review only - http://bmjopen.bmj.com/site/about/guidelines.xhtml

Table 4 – Testing for an association between awareness of the breast screening media coverage and frequency of worry about breast cancer*

		Never; occasionally vs. Sometimes; often; very often: n (%)		Adjusted OR, 95% CI	p-value
Characteristic	Total (n=700)	Never; occasionally (n=441; 63.0%)	Sometimes; often; very often (n=259; 37.0%)	Sometimes; often; very often (vs. Never; occasionally)	
**Screening story awareness**					Overall: .198
Aware of the main story and both follow-up commentaries	88	65 (73.9)	23 (26.1)	0.85, 0.46 to 1.58	.614
Aware of the main story and overdiagnosis follow-up	63	42 (66.7)	21 (33.3)	1.05, 0.55 to 2.01	.878
Aware of the main story and all-cause mortality follow-up	36	25 (69.4)	11 (30.6)	1.10, 0.49 to 2.49	.819
Aware of the main story only	270	153 (56.7)	117 (43.3)	1.49, 0.98 to 2.25	.062
vs. Unaware of the story	243	156 (64.2)	87 (35.8)		

*Results are adjusted for covariates: Recruitment wave, Age, Ethnicity, Marital status, Highest level of education, Social class grade, Employment status, Area type, Personal diagnosis of cancer, Personal experience of breast screening, Belief that screening is almost always a good idea, Awareness of volcano news, Awareness of election news, General level of trust in participants' GPs, General level of trust in the NHS, Breast screening intentions for next invitation. Full results of the model are reported in the Appendix

Table 5 – Testing for an association between awareness of the breast screening media coverage and breast screening intentions*

		Yes, definitely vs. Yes, probably; no, probably not; no, definitely not: n (%)		Adjusted OR, 95% CI	p-value
Characteristic	Total (n=700)	No definite intention (n=99; 14.1%)	Definite intention (n=601; 85.9%)	Definite intention (vs. No definite intention)	
**Screening story awareness**					Overall: .108
Aware of the main story and both follow-up commentaries	88	10 (11.4)	78 (88.6)	2.01, 0.74 to 5.48	.172
Aware of the main story and overdiagnosis follow-up	63	4 (4.3)	59 (93.7)	2.66, 0.79 to 8.89	.113
Aware of the main story and all-cause mortality follow-up	36	6 (16.7)	30 (83.3)	0.66, 0.20 to 2.13	.486
Aware of the main story only	270	22 (8.1)	248 (91.9)	1.88, 0.99 to 3.57	.054
vs. Unaware of the story	243	57 (23.5)	186 (76.5)		

*Results are adjusted for covariates: Recruitment wave, Age group, Ethnicity, Marital status, Highest level of education, Social class grade, Employment status, Area type, Personal diagnosis of cancer, Personal experience of breast screening, Belief that screening is always a good idea, Awareness of volcano news, Awareness of election news, General level of trust in participants' GPs, General level of trust in the NHS, Frequency of worry about breast cancer. Full results of the model are reported in the Appendix

For peer review only - http://bmjopen.bmj.com/site/about/guidelines.xhtml

DISCUSSION

Previous studies have found evidence that media messages can increase usage of a range of healthcare services (e.g. [7-10, 12-15]). Awareness of this story about errors in the breast screening programme was hypothesised to have the potential for a range of negative effects. However, the results of this study did not provide strong evidence against the null hypothesis for any associations tested. To the extent that these results reflect an absence of harms, this is reassuring: we did not find evidence that awareness of the story reduced trust in the NHS or participants' GPs, increased frequency of worry about breast cancer, or negatively affected future breast screening intentions. If this is the case, it may be partly attributable to the news story saying little to reduce the perceived benefits of breast screening itself, in contrast to media coverage of e.g. the independent review of breast cancer screening, which reported on the issue of overdiagnosis extensively.[23-24] Relatedly, the present study found that awareness was notably lower for follow-up commentaries on the shortcomings of breast screening, compared with the main story. In addition, the framing of the story may have been expected to reinforce the perceived benefits of screening by indicating that missing screening had negative consequences in terms of additional breast cancer deaths.

Population awareness of the breast screening news story was generally high. Television and radio were the main sources of information, broadly consistent with patterns of how most news is accessed, although the internet was used less often than observed in previous surveys.[25] Although no associations were found here, this finding is useful since it provides an estimate of the proportion of people who may be influenced by media coverage that does have positive or negative effects on health behaviour.[7-10, 12-15] In the absence of this study, a plausible rationale

For peer review only - http://bmjopen.bmj.com/site/about/guidelines.xhtml

could have been found for why this estimate would be higher or lower than was shown to be the case.

Awareness of this story was related to awareness of other news stories, suggesting that an appreciable proportion of the population can be broadly dichotomised into those who are generally “news aware” and “news unaware”. These results do not suggest that a notable proportion of the public are aware of health news, specifically. In contrast to these findings, recall of the main statistics was markedly low and correct responses may be largely attributable to random guessing.[1] In some respects, this is surprising since the statistics were an integral part of the story and often part of headlines (e.g. [2-4, 26]) and may be a cause for concern: the number of women affected and estimated to have died as a result are important pieces of information in order for an individual to make a personal assessment of the scale and severity of the news. This finding may suggest that people either tend not to attend to or memorise this statistical information (meaning that they would not be able to factor it into their appraisal of the significance of the story) or they retain only the ‘gist’ of the statistics involved.[27] Awareness of the breast screening story was greater among those with higher levels of education and social class grade, those who were white British, and those who were older. Awareness of the breast screening news story was also lower among participants with positive attitudes towards screening (who may have been less likely to attend to a negative story).

---

[1] Participants were asked additional questions on the extent to which they trusted the statistics and their reasons for not trusting them (if applicable). However, since responses were highly suggestive of random guessing, no further analyses of these measures were attempted.

For peer review only - http://bmjopen.bmj.com/site/about/guidelines.xhtml

This study has limitations. Despite the large sample size and adjustment for a range of potentially confounding variables, some odds ratios could not be estimated with a high degree of precision. Confidence intervals were wide for key variables, meaning that associations may not have been detected if they were real but smaller than observed. In addition, our measures did not include a question on trust in the Breast Screening Programme, specifically, meaning that we could not test for associations with this outcome. Findings on screening uptake also relate only to anticipated future behaviour; future research could build on this study by assessing whether the announcement was followed by a decrease (or increase) in actual screening uptake. Although the response rate to this survey was higher than others of its type (e.g. 71% in the present study vs. 42% reported by Low et al.),[28] members of the public were also less likely to participate in the survey module based on a range of characteristics for which data were available. Results may be biased, insofar as responses differed based on these variables or unmeasured participant characteristics that may have reduced population-representativeness of the sample.

Conclusions

This study found that news of errors in the Breast Screening Programme in England had reached a large proportion of the general public and that those aware of the media coverage tended to be those aware of news stories in general. The proportion of people aware was also higher among those who had more education, were in a higher social class grade, or were older. In contrast, awareness of key statistics from the story was very low among participants aware of the story, even less than six weeks after the onset of the main media coverage. The results of this study did not provide evidence that media coverage had any effects on trust in aspects of the health service among the general public, or worry about breast cancer or breast

For peer review only - http://bmjopen.bmj.com/site/about/guidelines.xhtml

screening intentions among women. Future research should investigate possible effects of media coverage using objective measures of screening behaviour.

Contributors: AG, CVW, and JW conceived and designed the study. AG analysed the data. AG, CVW, and JW participated in the interpretation of results. AG, CVW, and JW drafted the manuscript, participated in critical revision, and approved the final version. Patients and the public were not involved in this study.

Funding: This work was supported by a programme grant from Cancer Research UK awarded to Prof Jane Wardle [C1418/A14134]. Dr Jo Waller is supported by a Career Development Fellowship from Cancer Research UK [C7492/A17219]. Cancer Research UK was not involved in the design of this study; the collection, analysis, or interpretation of the results; in the writing of the manuscript; or in the decision to submit for publication.

Exclusive Licence: I, the Submitting Author has the right to grant and does grant on behalf of all authors of the Work (as defined in the below author licence), an exclusive licence and/or a non-exclusive licence for contributions from authors who are: i) UK Crown employees; ii) where BMJ has agreed a CC-BY licence shall apply, and/or iii) in accordance with the terms applicable for US Federal Government officers or employees acting as part of their official duties; on a worldwide, perpetual, irrevocable, royalty-free basis to BMJ Publishing Group Ltd ("BMJ") its licensees and where the relevant Journal is co-owned by BMJ to the co-owners of the Journal, to publish the Work in BMJ Open and any other BMJ products and to exploit all rights, as set out in our licence.

Competing interests: None declared.

Patient consent: Obtained.

For peer review only - http://bmjopen.bmj.com/site/about/guidelines.xhtml

Data sharing: No additional data are available.

For peer review only

For peer review only - http://bmjopen.bmj.com/site/about/guidelines.xhtml

REFERENCES

1. House of Commons Hansard. Breast cancer screening https://hansard.parliament.uk/commons/2018-05-02/debates/BE9DB48A-C9FF-401B-AC54-FF53BC5BD83E/BreastCancerScreening (2018, accessed 18 October 2018).
2. Mail Online. ‘They didn’t deserve to lose their lives’: Fury of husband whose wife died from breast cancer after ‘colossal’ NHS computer glitch which has seen 270 die and 450,000 miss screenings https://www.dailymail.co.uk/news/article-5682203/Hundreds-women-developed-breast-cancer-450-000-not-invited-screening.html (2018, accessed 18 October 2018).
3. The Guardian. Up to 270 women may have died after breast cancer screening IT error https://www.theguardian.com/society/2018/may/02/jeremy-hunt-to-launch-inquiry-into-450000-missed-breast-cancer-screenings (2018, accessed 18 October 2018).
4. BBC News. Breast screening error ‘shortened up to 270 lives’ – Hunt https://www.bbc.co.uk/news/health-43973652 (2018, accessed 18 October 2018).
5. Bewley S, Baum M, Hodkinson A, et al. Screening ‘flaw’. The Times, 05 May 2018
6. Spiegelhalter D. Have ‘up to 270 women died’ by missing a breast screening appointment letter? https://medium.com/wintoncentre/have-up-to-270-women-died-by-missing-a-breast-screening-appointment-letter-756f74c4f56b (2018, 18 October 2018).
7. Lancucki L, Sasieni P, Patnick J, et al. The impact of Jade Goody’s diagnosis and death on the NHS Cervical Screening Programme. J Med Screen 2012; 19:89–93.
8. MacArthur GJ, Wright M, Beer H, et al. Impact of media reporting of cervical cancer in a UK celebrity on a population-based cervical screening programme. J Med Screen 2011; 18:204–209.

For peer review only - http://bmjopen.bmj.com/site/about/guidelines.xhtml

9. Marlow LAV, Sangha A, Patnick J, et al. The Jade Goody Effect: Whose cervical screening decisions were influenced by her story? J Med Screen 2012; 19:184–188.

10. Casey GM, Morris B, Burnell M, et al. Celebrities and screening: a measurable impact on high-grade cervical neoplasia diagnosis from the “Jade Goody effect” in the UK. Br J Cancer 2013; 109:1192–1197.

11. Atkin WS, Edwards R, Kralj-Hans I, et al. Once-only flexible sigmoidoscopy screening in prevention of colorectal cancer: a multicentre randomised controlled trial. Lancet 2010; 375:1624–1633.

12. Lo S, Vart G, Snowball J, et al. The impact of media coverage of the Flexible Sigmoidoscopy Trial on English colorectal screening uptake. J Med Screening 2012; 19:83–88.

13. Moffat J, Bentley A, Ironmonger L, et al. The impact of national cancer awareness campaigns for bowel and lung cancer symptoms on sociodemographic inequalities in immediate key symptom awareness and GP attendances. Br J Cancer 2015; 112:S14–S21.

14. Bethune R, Marshall MJ, Mitchell SJ, et al. Did the “Be Clear on Bowel Cancer” public awareness campaign pilot result in a higher rate of cancer detection? Postgrad Med J 2013; 89:390–393.

15. Hughes-Hallett A, Browne D, Mensah E, et al. Assessing the impact of mass media public health campaigns. Be Clear on Cancer “blood in pee”: a case in point. BJU Int 2016; 117:570–575.

16. National Readership Survey. Social Grade http://www.nrs.co.uk/nrs-print/lifestyle-and-classification-data/social-grade (n.d., accessed 18 October 2018).

For peer review only

For peer review only - http://bmjopen.bmj.com/site/about/guidelines.xhtml

17. Schwartz LM, Woloshin S, Fowler FJ, Welch HG. Enthusiasm for cancer screening in the United States. J Am Med Assoc. 2004;291(1):71–8.
18. BBC News. Local election results 2018: No clear winner as Labour and Tories neck and neck https://www.bbc.co.uk/news/uk-politics-44014076 (2018, 18 October 2018).
19. BBC News. Kilauea: Hawaii emergency declared over volcano eruption https://www.bbc.co.uk/news/world-us-canada-44001651 (2018, 18 October 2018).
20. Health Information National Trends Survey 2015 and 2017 https://hints.cancer.gov (2018, 18 October 2018).
21. Rutten LJF, Blake KD, Skolnick VG, et al. Data resource profile: The national Cancer Institute's Health Information National Trends Survey (HINTS). Int J Epidemiol 2019; in press.
22. Health Information Trends Survey 2003, 2005, and 2008 https://hints.cancer.gov (2018, 18 October 2018).
23. Independent UK Panel on Breast Cancer Screening. The benefits and harms of breast cancer screening: an independent review. Lancet 2012; 380:1778-1786.
24. BBC News. Breast screening advice updated amid controversy over tests https://www.bbc.co.uk/news/health-20121043 (2012, 18 October 2018).
25. OFCOM. News consumption in the UK https://www.ofcom.org.uk/research-and-data/tv-radio-and-on-demand/news-media/news-consumption (2018, 18 October 2018).
26. Sky News. Breast cancer screening failure 'shortened' up to 270 lives https://news.sky.com/story/inquiry-into-breast-cancer-screening-failures-to-be-launched-say-sky-sources-11355751 (2018, 18 October 2018).
27. Reyna VF, Brainerd CJ. Fuzzy-trace theory. An interim synthesis. Learn Individ Differ. 1997; 7:1–75.

For peer review only - http://bmjopen.bmj.com/site/about/guidelines.xhtml

28. Low EL, Waller J, Wardle J, Menon U. Experience of symptoms indicative of hynaecological cancers in UK women. Br J Cancer. 2013; 109:882-887.

For peer review only

For peer review only - http://bmjopen.bmj.com/site/about/guidelines.xhtml

APPENDIX 1 - SURVEY

[All Adults 16+ in England. Participants were shown the tablet screen and the following text was read out by interviewers]

Q.A In this part of the survey, I am going to ask you some questions related to health, including cancer, and recent news stories. These questions are asked on behalf of researchers from University College London. If you do not wish to answer a particular question during any part of this survey, you may refuse to answer and we will move to the next question. All your answers will be kept strictly confidential and you will be anonymous to the researchers.

The NHS currently offers breast cancer screening with mammography once every three years, to women aged between about 50 to 70 years in England.

Are you okay to continue with these questions?

1: Yes

2: No

As the questions can be perceived as sensitive, you can answer the questions on this machine yourself. I would now like to show you how to use the machine by going through a practice question with you.

[All Adults 16+ in England willing to continue. “Don’t know”/“Not sure”/“Prefer not to say” appeared at the top of the screen, out of view of participants, except for questions that participants completed themselves. Interviewers showed the screen to participants]

This is an example of a single-coded question

Q.B What is your favourite colour?

1: Red

2: Yellow

For peer review only

3: Blue

4: Green

Other colour (PEN -WRITE IN)

Don't know

Refused

[All females aged 16-69 in England willing to continue. Interviewers handed tablets to participants and stepped away from viewing the screen]

Q.1 Do you think you will go for breast screening when you are next offered it? REMEMBER TO TAP OK TO CONTINUE

1: Yes, definitely

2: Yes, probably

3: No, probably not

4: No, definitely not

Not sure

Prefer not to say

[All females aged 47+ in England willing to continue]

Q.2 Have you ever been invited for breast screening before? If you've only ever been offered a mammogram to investigate symptoms separately to the screening programme, please respond 'no'.

1: Yes

2: No

For peer review only

Not sure

Prefer not to say

[All females aged 47+ in England who have been invited for breast screening before]

Q.3 Have you ever been for breast screening as part of the screening programme?

1: Yes

2: No

Not sure

Prefer not to say

[All Adults 16+ in England willing to continue. This question allowed more than one response option. “None of the above”/”Prefer not to say” were mutually exclusive with other responses]

Q.4 Which of the following, if any, have you been diagnosed with? Please choose all that apply.

1: Bowel cancer

2: Lung cancer

3: Breast cancer

4: Cervical cancer

5: Prostate cancer

Other type of cancer - PEN WRITE IN

None of the above

Prefer not to say

For peer review only

[All Adults 16+ in England willing to continue. This question allowed more than one response option. "None of the above"/"Prefer not to say" were mutually exclusive with other responses]

Q.5 Has anyone you know ever been diagnosed with breast cancer? Please choose all that apply.

1: A close family member

2: Any other family member

3: A friend

4: A colleague

5: Any other person

Not sure

Prefer not to say

[All females aged 16+ in England willing to continue]

Q.6 How often do you worry about your chances of getting breast cancer yourself?

1: Never

2: Occasionally

3: Sometimes

4: Often

5: Very often

Not sure

Prefer not to say

Thank you for answering these questions - this is the end of this section for you.

For peer review only

[All Adults 16+ in England willing to continue. Participants handed the tablet back to the interviewer, who showed the screen and either read out or allowed participants to read subsequent questions]

Q.7 In May, it was reported that a computer algorithm failure had meant that a number of women did not receive invitations to their final routine breast cancer screening. The Health Secretary, Jeremy Hunt, said that women affected will be contacted by letter with an invitation for a catch-up screening test but some of the women who were not invited for their final appointment may have had their lives shortened.

Do you recall seeing or hearing anything about this news story before now?

1: Yes

2: No

Not sure

[All who recall seeing or hearing anything about this news story before now. This was a multiple choice question. The order of response options was randomised with “other websites” always following both “online news websites” and “social media websites”]

Q.8 Do you recall where you saw or heard this news story? Please choose all that apply.

1: Television

2: Print newspaper(s)

3: Radio

4: Online news websites

5: Social media websites

6: Other websites

For peer review only

7: Word of mouth

Other sources – PEN WRITE IN

Not sure

[All who recall seeing or hearing anything about this news story before now]

Q.9 Did you discuss or share the story with anyone else?

1: Yes

2: No

Not sure

[All who recall seeing or hearing anything about this news story before now. Participants were randomised to one of two orders of response options (1:1)]

Q.10 The Health Secretary, Jeremy Hunt, gave an estimate of the number of women who had failed to get invitations since 2009.

Which of the following do you think is the estimate that he gave?

1: 4,500 women

2: 45,000 women

3: 450,000 women

4: 4,500,000 women

Not sure

For peer review only

[All who recall seeing or hearing anything about this news story before now. Participants randomised to one of two orders of response options (1:1)]

Q.11 The Health Secretary also gave an estimate, based on computer modelling, of the number of women who may have had their lives shortened.

Which of the following do you think is the estimate that he gave?

1: Between 13 and 27 women

2: Between 135 and 270 women

3: Between 13 and 2,700 women

4: Between 1,350 and 2,700 women

Not sure

[All who gave an estimate in Q1 or Q11]

Q.12 How much did you trust these statistics when you heard them in the news?

1: Not at all

2: A little

3: Somewhat

4: A lot

Not sure

[All who do not trust the statistic]

Q.13 What were your reasons for not trusting these statistics when you heard them in the news?

PROBE: Any other reasons?

For peer review only

OPEN ENDED

[All who recall seeing or hearing anything about this news story before now]

Q.14 It was also reported that some health experts have said breast cancer screening can do “more harm than good” because they believe “breast screening…has no impact on all-cause death”.

Do you recall seeing or hearing anything about this aspect of the news story before now?

1: Yes

2: No

Not sure

[All who recall seeing or hearing anything about this news story before now]

Q.15 The estimate of the number of women who may have had their lives shortened that the Health Secretary gave was between 135 and 270. It was also reported that one statistics expert has said this claim is “misleading” because they believe “there is only weak evidence that screening helps prolong life, particularly for older women” and that “contrary to popular belief, screening also does harm...for every 200 women attending screening between 50 and 70, we would expect one to have her early death from breast cancer prevented, but three to be unnecessarily treated for a harmless cancer that would not have troubled them".

Do you recall seeing or hearing anything about this aspect of the news story before now?

1: Yes

2: No

Not sure

For peer review only

[All Adults 16+ in England willing to continue]

Q.16 In general, how much do you trust your general practitioner?

1: Not at all

2: A little

3: Somewhat

4: A lot

Not sure

[All Adults 16+ in England willing to continue]

Q.17 In general, how much do you trust the NHS?

1: Not at all

2: A little

3: Somewhat

4: A lot

Not sure

[All Adults 16+ in England willing to continue]

Q.18 Routine screening means testing healthy people to find cancer before they have any symptoms.

Do you think routine cancer screening tests for healthy people are almost always a good idea?

1: Yes

2: No

For peer review only

Not sure

[Participants were handed an information card with the following text and asked to read it]

You may have some questions about breast cancer screening after this part of the survey. You can find out more by calling the NHS on a Freephone number (0800 169 2692) or via the web on https:\\www.nhs.uk\conditions\breast-cancer-screening\missed-invitations\.

[All Adults 16+ in England willing to continue]

Q.19 It was also reported in May that a volcano had erupted in Hawaii, leading to officials declaring a state of emergency and mandatory evacuation of 1,700 residents in the area.

Do you recall seeing or hearing anything about this news story before now?

1: Yes

2: No

Not sure

[All Adults 16+ in England willing to continue]

Q.20 The results of local elections held in England were also reported in May. The Labour Party won 2,350 seats, the Conservative Party won 1,332 seats, and the Liberal Democrats won 536 seats.

Do you recall seeing or hearing anything about this news story before now?

1: Yes

2: No

Not sure

For peer review only

[All Adults 16+ in England willing to continue]

Q.21 Please can I ask you what is the highest level of qualification you have received?

1: A. Graduate level qualifications and above: including higher degrees, professional qualifications at HE standard (e.g. chartered accountant, surveyor, Nursing, Teaching), NVQ and SVQ Level 4 or 5, Higher Education Diplomas, HNC and HND and BTEC Higher, RSA Higher Diploma

2: B. A-levels and AS levels and equivalents: including SCE Higher, Scottish Certificate 6th Year Studies, NVQ and SVQ and GSVQ level 3, GNVQ Advanced, ONC and OND and BTEC National, City and Guilds Advanced Craft, City and Guilds Final level or Part III, RSA Advanced Diploma

3: C. Trade apprenticeships

4: D. GCSEs and equivalents: including O level, SCE Standard, CSEs, NVQ and SVQ and GSVQ level 1 and 2, GNVQ and BTEC and SCOTVEC first, General diploma, City and Guilds Ordinary level, City and Guilds Ordinary level Part II, RSA State I-III or Diploma, SCOTVEC modules

5: E. Other qualifications (including overseas)

6: F. No formal qualifications

Don’t know

Refused

For peer review only

APPENDIX 2 – SUPPLMENTARY TABLES

Table A – Summary statistics describing the sample

Measure	Total* (n=1,894)	%	(95% CI)
**Awareness of the news about breast screening**			
Aware of the main story and both follow-up commentaries	250	13.2	11.7 to 14.8
Aware of the main story and overdiagnosis follow-up	188	9.9	8.6 to 11.3
Aware of the main story and all-cause mortality follow-up	117	6.2	5.2 to 7.3
Aware of the main story only	709	37.4	35.3 to 39.6
Unaware of the story	630	33.3	31.2 to 35.4
**Recruitment wave**			
Wave 2: 20-26th June	606	32.0	29.9 to 34.1
Wave 1: 6-10th June	1,288	68.0	65.9 to 70.1
**Gender**			
Male	801	42.3	40.1 to 44.5
Female	1,093	57.7	55.5 to 59.9
**Ethnicity**			
White British	1,555	82.4	80.7 to 84.1
Other groups	331	17.6	15.9 to 19.3
**Marital status**			
Married or living as a married	1,039	54.9	52.6 to 57.1
Widowed, divorced and separated	382	20.2	18.4 to 22.0
Single	473	25.0	23.1 to 27.0
**Highest level of education**			
Graduate level qualifications and above	530	28.2	26.2 to 30.3
A-levels and AS levels and equivalents	461	24.6	22.7 to 26.5
GCSEs and equivalents	459	24.5	22.5 to 26.4
Trade apprenticeships or other qualifications	93	5.0	4.0 to 6.0
No formal qualifications	334	17.8	16.1 to 19.6
**Social class grade**			
Grade A or B	336	17.7	16.1 to 19.5
Grade C1	539	28.5	26.5 to 30.5
Grade C2	423	22.3	20.5 to 24.3
Grade D or E	596	31.5	29.4 to 33.6
**Employment status**			
Working	862	45.5	43.3 to 47.8
Not working	1,032	54.5	52.2 to 56.7
**Area type**			
Urban	1,542	81.4	79.6 to 83.1
Rural	352	18.6	16.9 to 20.4
**Personal diagnosis of cancer**			
Yes	156	8.4	7.2 to 9.7
No	1,705	91.6	90.3 to 92.8
**Personal experience of breast screening**			
Taken part	441	23.7	21.8 to 25.7
Invited, never taken part	56	3.0	2.3 to 3.9
Not eligible or not invited	1,364	73.3	71.2 to 75.3
**Belief that screening is almost always a good idea**			
Yes	1,737	91.7	90.4 to 92.9
No or not sure	157	8.3	7.1 to 9.6
**Awareness of the news about the volcanic eruption**			
Yes	1,435	75.8	73.8 to 77.7
No or not sure	459	24.2	22.3 to 26.2
**Awareness of the news about the local elections**			
Yes	1,198	63.3	61.1 to 65.4
No or not sure	696	36.7	34.6 to 38.9
**General level of trust in participants' GPs**			
A lot	1,009	55.2	52.9 to 57.5
Somewhat	540	29.6	27.5 to 31.7
A little	219	12.0	10.6 to 13.5
Not at all	59	3.2	2.5 to 4.1
**General level of trust in the NHS**			

For peer review only - http://bmjopen.bmj.com/site/about/guidelines.xhtml

A lot	1,016	54.2	54.9 to 59.5
Somewhat	619	33.0	32.7 to 37.1
A little	184	9.8	9.0 to 11.8
Not at all	56	3.0	2.4 to 4.0
**Frequency of worry about breast cancer**			
Very often	46	4.6	3.4 to 6.0
Often	55	5.4	4.2 to 7.0
Sometimes	216	21.4	18.9 to 24.0
Occasionally	302	29.9	27.1 to 32.8
Never	391	38.7	35.7 to 41.7
**Breast screening intentions for next invitation**			
Yes, definitely	690	84.7	82.1 to 87.0
Yes, probably	88	10.8	8.8 to 13.1
No, probably not	18	2.2	1.4 to 3.4
No, definitely not	19	2.3	1.5 to 3.5

Table B – Full results of the binary logistic regression model testing for variables associated with whether participants responded to questions on the survey module

		**Responded vs. Did not respond to the survey questions: n (%)**		**Adjusted OR, 95% CI**	**p-value**
**Characteristic**	**Total (n=2,665)**	**Did not respond (n=779; 29.2%)**	**Responded (n=1,886; 70.8%)**	**Responded to questions (vs. Did not respond)**	
**Recruitment wave**					
Wave 2: 20-26th June	908	303 (33.4)	605 (66.6)	0.73, 0.61 to 0.87	**<.0005**
vs. Wave 1: 6-10th June	1,757	476 (27.1)	1,281 (72.9)		
**Gender**					
Male	1,270	474 (37.3)	796 (62.7)	0.46, 0.39 to 0.55	**<.0005**
vs. Female	1,395	305 (21.9)	1,090 (78.1)		
**Ethnicity**					
White British	2,139	584 (27.3)	1,555 (72.7)	1.69, 1.37 to 2.10	**<.0005**
vs. Other groups	526	195 (37.1)	331 (62.9)		
**Marital status**					Overall: **.001**
Married/Living as a couple	1,441	407 (28.2)	1,034 (71.8)	1.48, 1.18 to 1.85	**.001**
Widowed/Divorced/Separated	517	135 (26.1)	382 (73.9)	1.65, 1.21 to 2.24	**.002**
vs. Single	707	237 (33.5)	470 (66.5)		
**Social class grade**					Overall: **.003**
Grade A or B	450	115 (25.6)	335 (74.4)	1.54, 1.18 to 2.02	**.002**
Grade C1	726	190 (26.2)	536 (73.8)	1.44, 1.15 to 1.81	**.002**
Grade C2	596	174 (29.2)	422 (70.8)	1.28, 1.01 to 1.63	**.045**
vs. Grade D or E	893	300 (33.6)	593 (66.4)		
**Employment status**					
Working	1,225	366 (29.9)	859 (70.1)	0.79, 0.65 to 0.97	**.026**
vs. Not working	1,440	413 (28.7)	1,027 71.3)		
**Area type**					
Urban	2,164	629 (29.1)	1,535 (70.9)	1.14, 0.91 to 1.42	.246
vs. Rural	501	150 (29.9)	351 (70.1)		
**Age (in years)**	2,665	52.1 (21.0)	50.8 (20.5)	0.99, 0.98 to 1.00	**<.0005**

Table C – Sources of news about the breast screening story

**Source of information**	**Total (n=1,264)**	**%**	**(95% CI)**
Television	971	76.8	74.4 to 79.1
Radio	271	21.4	19.2 to 23.8
Print newspaper(s)	169	13.4	11.6 to 15.3
Online news websites	134	10.6	9.0 to 12.4
Social media websites	68	5.4	4.2 to 6.7
Other websites	11	0.9	0.5 to 1.5

For peer review only - http://bmjopen.bmj.com/site/about/guidelines.xhtml

Word of mouth	43	3.4	2.5 to 4.5
Other sources	8	0.6	0.3 to 1.2
Discussed or shared the story with someone else	450	35.6	33.0 to 38.3

Table D – Full results of the binary logistic regression model testing for an association between awareness of the breast screening media coverage and trust in participants' GPs

		A lot vs. Not at all; a little; somewhat: n (%)		Adjusted OR, 95% CI	p-value
**Characteristic**	**Total (n=1,746)**	**Less than a lot (n=781; 44.7%)**	**A lot (n=965; 55.3%)**	**A lot (vs. Less than a lot)**	
**Screening story awareness**					Overall: .729
Aware of the main story and both follow-up commentaries	238	98 (41.2)	140 (58.8)	1.10, 0.74 to 1.64	.653
Aware of the main story and overdiagnosis follow-up	172	66 (38.4)	106 (61.6)	1.31, 0.85 to 2.03	.218
Aware of the main story and all-cause mortality follow-up	107	49 (45.8)	58 (54.2)	1.21, 0.73 to 2.02	.459
Aware of the main story only	655	280 (42.7)	375 (57.3)	1.17, 0.88 to 1.57	.283
vs. Unaware of the story	574	288 (50.2)	286 (49.8)		
**Recruitment wave**					
Wave 2: 20-26th June	557	255 (45.8)	663 (55.8)	0.81, 0.64 to 1.04	.097
vs. Wave 1: 6-10th June	1,189	526 (44.2)	302 (54.2)		
**Gender**					
Male	754	317 (42.0)	437 (58.0)	1.15, 0.86 to 1.54	.334
vs. Female	992	464 (46.8)	528 (53.2)		
**Ethnicity**					
White British	1,450	614 (42.3)	836 (57.7)	1.17, 0.85 to 1.61	.328
vs. Other groups	296	167 (54.4)	129 (43.6)		
**Marital status**					Overall: .504
Married/Living as a couple	964	422 (42.8)	542 (56.2)	1.04, 0.77 to 1.42	.782
Widowed/Divorced/Separated	341	139 (40.8)	202 (59.2)	0.86, 0.57 to 1.29	.460
vs. Single	441	220 (49.9)	221 (50.1)		
**Highest level of education**					Overall: .056
Graduate level/Above	494	230 (46.6)	264 (53.4)	0.64, 0.42 to 0.98	.042
A-levels/AS levels/Equivalents	438	201 (45.9)	237 (54.1)	0.70, 0.46 to 1.06	.089
GCSEs/Equivalents	429	213 (49.7)	216 (50.3)	0.63, 0.43 to 0.94	.022
Trade apprenticeships/Other	86	41 (47.7)	45 (52.3)	0.44, 0.25 to 0.80	.007
vs. No formal qualifications	299	96 (32.1)	203 (67.9)		
**Social class grade**					Overall: .711
Grade A or B	317	128 (40.4)	189 (59.6)	1.20, 0.82 to 1.76	.342
Grade C1	505	231 (45.7)	274 (54.3)	1.02, 0.74 to 1.39	.923
Grade C2	385	181 (47.0)	204 (53.0)	0.97, 0.70 to 1.36	.874
vs. Grade D or E	539	241 (44.7)	298 (55.3)		
**Employment status**					
Working	806	411 (51.0)	395 (49.0)	0.82, 0.63 to 1.07	.135
vs. Not working	940	370 (39.4)	570 (60.6)		
**Area type**					
Urban	1,420	635 (44.7)	785 (55.3)	1.12, 0.84 to 1.50	.430
vs. Rural	326	146 (44.8)	180 (55.2)		
**Personal diagnosis of cancer**					
Yes	1,599	718 (44.9)	881 (55.1)	0.84, 055 to 1.28	.404
vs. No	147	63 (42.9)	84 (57.1)		
**Personal experience of breast screening**					Overall: .284
Taken part	411	159 (38.7)	252 (61.3)	1.33, 0.91 to 1.95	.145
Invited, never taken part	48	21 (43.8)	27 (56.3)	0.95, 0.46 to 1.98	.894
vs. Not eligible or not invited	1,287	601 (46.7)	686 (53.3)		

For peer review only

For peer review only - http://bmjopen.bmj.com/site/about/guidelines.xhtml

**Belief that screening is almost always a good idea**					
Yes	1,609	701 (43.6)	908 (56.4)	1.30, 0.85 to 1.97	.230
vs. No or not sure	137	80 (58.4)	57 (41.6)		
**Awareness of volcano news**					
Yes	1,332	565 (42.4)	767 (57.6)	1.04, 0.77 to 1.40	.789
vs. No or not sure	414	216 (52.2)	198 (47.8)		
**Awareness of election news**					
Yes	1,114	467 (41.9)	647 (58.1)	1.19, 0.93 to 1.54	.172
vs. No or not sure	632	314 (49.7)	318 (50.3)		
**General level of trust in the NHS**					Overall: **<.0005**
A lot	943	202 (21.4	741 (78.6)	13.53, 6.65 to 27.54	**<.0005**
Somewhat	589	409 (69.4)	180 (30.6)	1.62, 0.80 to 3.31	.183
A little	163	130 (79.8)	33 (20.2)	1.00, 0.46 to 2.21	.994
vs. Not at all	51	40 (78.4)	11 (21.6)		
**Age (in years)**	1,746	46.9 (19.1)	53.9 (21.0)	1.01, 1.00 to 1.02	.087

Table E – Full results of the binary logistic regression model testing for an association between awareness of the breast screening media coverage and trust in the NHS

		**A lot vs. Not at all; a little; somewhat: n (%)**		**Adjusted OR, 95% CI**	**p-value**
**Characteristic**	**Total (n=1,746)**	**Less than a lot (n=803; 46.0%)**	**A lot (n=943; 54.0%)**	**A lot (vs. Less than a lot)**	
**Screening story awareness**					Overall: .290
Aware of the main story and both follow-up commentaries	238	102 (42.9)	136 (57.1)	0.87, 0.59 to 1.30	.503
Aware of the main story and overdiagnosis follow-up	172	76 (44.2)	96 (55.8)	0.78, 0.51 to 1.21	.267
Aware of the main story and all-cause mortality follow-up	107	57 (53.3)	50 (46.7)	0.58, 0.35 to 0.97	.039
Aware of the main story only	655	299 (45.6)	356 (54.4)	0.81, 0.60 to 1.09	.160
vs. Unaware of the story	574	269 (46.9)	305 (53.1)		
**Recruitment wave**					
Wave 2: 20-26th June	557	248 (44.5)	309 (55.5)	1.21, 0.95 to 1.55	.118
vs. Wave 1: 6-10th June	1,189	555 (46.7)	634 (53.3)		
**Age**					Overall: .052
65+	530	198 (37.4)	332 (62.6)	1.04, 0.61 to 1.79	.880
55-64	245	111 (45.3)	134 (54.7)	0.76, 0.44 to 1.30	.309
45-54	235	115 (48.9)	120 (51.1)	0.83, 0.50 to 1.39	.484
35-44	245	135 (55.1)	110 (44.9)	0.59, 0.36 to 0.96	.035
25-34	265	152 (57.4)	113 (42.6)	0.56, 0.35 to 0.91	.018
vs. 16-24	226	92 (40.7)	134 (59.3)		
**Gender**					
Male	754	319 (42.3)	435 (57.7)	0.99, 0.73 to 1.33	.985
vs. Female	992	484 (48.8)	508 (51.2)		
**Ethnicity**					
White British	1,450	634 (43.7)	816 (56.3)	1.47, 1.07 to 2.02	**.019**
vs. Other groups	296	169 (57.1)	127 (42.9)		
**Marital status**					Overall: .870
Married/Living as a couple	964	440 (45.6)	524 (54.4)	1.07, 0.78 to 1.47	.685
Widowed/Divorced/Separated	341	151 (44.3)	190 (55.7)	1.00, 0.66 to 1.50	.990
vs. Single	441	212 (48.1)	229 (51.9)		
**Highest level of education**					Overall: .076
Graduate level/Above	494	233 (47.2)	261 (52.8)	1.20, 0.79 to 1.83	.386
A-levels/AS levels/Equivalents	438	199 (45.4)	239 (54.6)	1.12, 0.75 to 1.67	.582
GCSEs/Equivalents	429	224 (52.2)	205 (47.8)	0.86, 0.59 to 1.26	.447
Trade apprenticeships/Other	86	32 (37.2)	54 (62.8)	1.85, 1.01 to 3.39	.047
vs. No formal qualifications	299	115 (38.5)	184 (61.5)		

For peer review only

For peer review only - http://bmjopen.bmj.com/site/about/guidelines.xhtml

**Social class grade**					Overall: .990
Grade A or B	317	140 (44.2)	177 (55.8)	0.96, 0.65 to 1.41	.828
Grade C1	505	232 (45.9)	273 (54.1)	0.99, 0.72 to 1.37	.968
Grade C2	385	179 (46.5)	206 (53.5)	1.02, 0.73 to 1.43	.892
vs. Grade D or E	539	252 (46.8)	206 (53.5)		
**Employment status**					
Working	806	410 (50.9)	396 (49.1)	0.94, 0.71 to 1.25	.673
vs. Not working	940	393 (41.8)	547 (58.2)		
**Area type**					
Urban	1,420	656 (46.2)	764 (53.8)	0.96, 0.72 to 1.29	.795
vs. Rural	326	147 (45.1)	179 (54.9)		
**Personal diagnosis of cancer**					
Yes	1,599	737 (46.1)	862 (53.9)	1.00, 0.66 to 1.50	.994
vs. No	147	66 (44.9)	81 (55.1)		
**Personal experience of breast screening**					Overall: .062
Taken part	411	193 (47.0)	218 (53.0)	0.65, 0.43 to 0.97	.035
Invited, never taken part	48	20 (41.7)	28 (58.3)	1.10, 0.51 to 2.35	.813
vs. Not eligible or not invited	1,287	590 (45.8)	697 (54.2)		
**Belief that screening is almost always a good idea**					
Yes	1,609	715 (44.4)	894 (55.6)	1.96, 1.28 to 3.00	**.002**
vs. No or not sure	137	88 (64.2)	49 (35.8)		
**Awareness of volcano news**					
Yes	1,332	594 (44.6)	738 (55.4)	1.00, 0.74 to 1.35	.987
vs. No or not sure	414	209 (50.5)	205 (49.5)		
**Awareness of election news**					
Yes	1,114	491 (44.1)	623 (55.9)	1.03, 0.80 1.33	.817
vs. No or not sure	632	312 (49.4)	320 (50.6)		
**General level of trust in participants' GPs**					Overall: **<.0005**
A lot	965	224 (23.2)	741 (76.8)	11.98, 6.07 to 23.64	**<.0005**
Somewhat	513	370 (72.1)	143 (27.9)	1.39, 0.70 to 2.76	.350
A little	212	165 (77.8)	47 (22.2)	1.12, 0.54 to 2.33	.770
vs. Not at all	56	44 (78.6)	12 (21.4)		

Table F – Full results of the binary logistic regression model testing for an association between awareness of the breast screening media coverage and frequency of worry about breast cancer

		**Never; occasionally vs. Sometimes; often; very often: n (%)/M (SD)**		**Adjusted OR, 95% CI**	**p-value**
**Characteristic**	**Total (n=700)**	**Never; occasionally (n=441; 63.0%)**	**Sometimes; often; very often (n=259; 37.0%)**	**Sometimes; often; very often (vs. Never; occasionally)**	
**Screening story awareness**					Overall: .198
Aware of the main story and both follow-up commentaries	88	65 (73.9)	23 (26.1)	0.85, 0.46 to 1.58	.614
Aware of the main story and overdiagnosis follow-up	63	42 (66.7)	21 (33.3)	1.05, 0.55 to 2.01	.878
Aware of the main story and all-cause mortality follow-up	36	25 (69.4)	11 (30.6)	1.10, 0.49 to 2.49	.819
Aware of the main story only	270	153 (56.7)	117 (43.3)	1.49, 0.98 to 2.25	.062
vs. Unaware of the story	243	156 (64.2)	87 (35.8)		
**Recruitment wave**					
Wave 2: 20-26th June	229	152 (66.4)	77 (33.6)	0.83, 0.58 to 1.19	.304
vs. Wave 1: 6-10th June	471	289 (61.4)	182 (38.6)		
**Ethnicity**					
White British	563	354 (62.9)	209 (37.1)	0.90, 0.58 to 1.39	.635
vs. Other groups	137	87 (63.5)	50 (36.5)		
**Marital status**					Overall: .272

For peer review only - http://bmjopen.bmj.com/site/about/guidelines.xhtml

Married/Living as a couple	403	255 (63.3)	148 (36.7)	1.14, 0.76 to 1.72	.519
Widowed/Divorced/Separated	100	58 (58.0)	42 (42.0)	1.61, 0.90 to 2.87	.110
vs. Single	197	128 (65.0)	69 (35.0)		
**Highest level of education**					Overall: **.026**
Graduate level/Above	230	149 (64.8)	81 (35.2)	0.40, 0.21 to 0.79	**.008**
A-levels/AS levels/Equivalents	206	138 (67.0)	68 (33.0)	0.67, 0.19 to 0.70	**.002**
GCSEs/Equivalents	176	105 (59.7)	71 (40.3)	0.57, 0.31 to 1.04	.068
Trade apprenticeships/Other	17	12 (70.6)	5 (29.4)	0.33, 0.10 to 1.10	.072
vs. No formal qualifications	71	37 (52.1)	34 (47.9)		
**Social class grade**					Overall: .704
Grade A or B	125	84 (67.2)	41 (32.8)	1.07, 0.60 to 1.90	.819
Grade C1	209	123 (58.9)	86 (41.1)	1.31, 0.80 to 2.13	.282
Grade C2	165	105 (63.6)	60 (36.4)	1.11, 0.67 to 1.83	.691
vs. Grade D or E	201	129 (64.2)	72 (35.8)		
**Employment status**					
Working	392	239 (61.0)	153 (39.0)	1.15, 0.81 to 1.64	.435
vs. Not working	308	202 (65.6)	106 (34.4)		
**Area type**					
Urban	574	366 (63.8)	208 (36.2)	0.83, 054 to 1.26	.378
vs. Rural	126	75 (59.5)	51 (40.5)		
**Personal diagnosis of cancer**					
Yes	44	26 (59.1)	18 (40.9)	1.62, 0.82 to 3.22	.169
vs. No	656	415 (63.3)	241 (36.7)		
**Personal experience of breast screening**					Overall: .428
Taken part	221	151 (68.3)	70 (31.7)	0.66, 0.36 to 1.23	.193
Invited, never taken part	34	23 (67.6)	11 (32.4)	0.75, 0.30 to 1.89	.537
vs. Not eligible or not invited	445	267 (60.0)	178 (40.0)		
**Belief that screening is almost always a good idea**					
Yes	660	412 (62.4)	248 (37.6)	1.12, 0.50 to 2.51	.779
vs. No or not sure	40	29 (72.5)	11 (27.5)		
**Awareness of volcano news**					
Yes	505	323 (64.0)	182 (36.0)	0.91, 0.61 to 1.36	.645
vs. No or not sure	195	118 (60.5)	77 (39.5)		
**Awareness of election news**					
Yes	424	275 (64.9)	149 (35.1)	0.84, 0.59 to 1.20	.340
vs. No or not sure	276	166 (60.1)	110 (39.9)		
**General level of trust in participants' GPs**					Overall: .791
A lot	339	220 (64.9)	119 (35.1)	1.26, 0.50 to 3.15	.626
Somewhat	221	131 (59.3)	90 (40.7)	1.47, 0.58 to 3.72	.412
A little	109	69 (63.3)	40 (36.7)	1.25, 0.49 to 3.22	.641
vs. Not at all	31	21 (67.7)	10 (32.3)		
**General level of trust in the NHS**					Overall: .744
A lot	344	223 (64.8)	121 (35.2)	1.14, 0.41 to 3.21	.802
Somewhat	257	151 (58.8)	106 (41.2)	1.39, 0.49 to 3.91	.535
A little	76	51 (67.1)	25 (32.9)	1.13, 0.38 to 3.36	.833
vs. Not at all	23	16 (69.6)	7 (30.4)		
**Breast screening intentions for next invitation**					Overall: .163
Yes, definitely	601	369 (61.4)	232 (38.6)	1.48, 0.47 to 4.68	.503
Yes, probably	70	49 (70.0)	21 (30.0)	0.93, 0.27 to 3.25	.913
No, probably not	13	12 (92.3)	1 (7.7)	0.25, 0.02 to 2.70	.254
vs. No, definitely not	16	11 (68.8)	5 (31.3)		
**Age (in years)**	700	43.7 (15.5)	41.8 (14.3)	0.99, 0.97 to 1.01	.463

Table G – Full results of the binary logistic regression model testing for an association between awareness of the breast screening media coverage and breast screening intentions

For peer review only - http://bmjopen.bmj.com/site/about/guidelines.xhtml

Characteristic	Total (n=700)	Yes, definitely vs. Yes, probably; no, probably not; no, definitely not: n (%) — No definite intention (n=99; 14.1%)	Definite intention (n=601; 85.9%)	Adjusted OR, 95% CI — Definite intention (vs. No definite intention)	p-value
**Screening story awareness**					Overall: .108
Aware of the main story and both follow-up commentaries	88	10 (11.4)	78 (88.6)	2.01, 0.74 to 5.48	.172
Aware of the main story and overdiagnosis follow-up	63	4 (4.3)	59 (93.7)	2.66, 0.79 to 8.89	.113
Aware of the main story and all-cause mortality follow-up	36	6 (16.7)	30 (83.3)	0.66, 0.20 to 2.13	.486
Aware of the main story only	270	22 (8.1)	248 (91.9)	1.88, 0.99 to 3.57	.054
vs. Unaware of the story	243	57 (23.5)	186 (76.5)		
**Recruitment wave**					
Wave 2: 20-26th June	229	40 (17.5)	189 (82.5)	0.71, 0.42 to 1.21	.211
vs. Wave 1: 6-10th June	471	59 (12.5)	412 (87.5)		
**Age**					Overall: .050
65+	69	7 (10.1)	62 (89.9)	0.84, 0.13 to 5.37	.855
55-64	123	5 (4.1)	118 (95.9)	2.49, 0.42 to 14.74	.313
45-54	128	10 (7.8)	118 (92.2)	3.59, 1.10 to 11.69	.034
35-44	143	21 (14.7)	122 (85.3)	2.72, 1.16 to 6.41	.022
25-34	144	29 (20.1)	115 (79.9)	2.82, 1.24 to 6.42	.014
vs. 16-24	93	27 (29.0)	66 (71.0)		
**Ethnicity**					
White British	563	72 (12.8)	491 (87.2)	0.96, 0.51 to 1.83	.905
vs. Other groups	137	27 (19.7)	110 (80.3)		
**Marital status**					Overall: .321
Married/Living as a couple	403	49 (12.2)	354 (87.8)	0.99, 0.53 to 1.84	.970
Widowed/Divorced/Separated	100	6 (6.0)	94 (94.0)	2.18, 0.73 to 6.53	.163
vs. Single	197	44 (22.3)	153 (77.7)		
**Highest level of education**					Overall: .169
Graduate level/Above	230	35 (15.2)	195 (84.8)	0.69, 0.22 to 2.20	.533
A-levels/AS levels/Equivalents	206	35 (17.0)	171 (83.0)	0.87, 0.29 to 2.62	.808
GCSEs/Equivalents	176	19 (10.8)	157 (89.2)	1.92, 0.62 to 5.92	.259
Trade apprenticeships/Other	17	3 (17.6)	14 (82.4)	1.38, 0.24 to 0.81	.720
vs. No formal qualifications	71	7 (9.9)	64 (90.1)		
**Social class grade**					Overall: .186
Grade A or B	125	14 (11.2)	111 (88.8)	1.87, 0.76 to 4.61	.177
Grade C1	209	25 (12.0)	184 (88.0)	2.36, 1.08 to 5.16	.032
Grade C2	165	24 (14.5)	141 (85.5)	1.85, 0.86 to 3.98	.118
vs. Grade D or E	201	36 (17.9)	165 (82.1)		
**Employment status**					
Working	392	56 (14.3)	336 (85.7)	0.70, 0.39 to 1.26	.238
vs. Not working	308	43 (14.0)	265 (86.0)		
**Area type**					
Urban	574	83 (14.5)	491 (85.5)	0.79, 0.39 to 1.59	.512
vs. Rural	126	16 (12.7)	110 (87.3)		
**Personal diagnosis of cancer**					
Yes	44	7 (15.9)	37 (84.1)	0.94, 0.30 to 2.98	.918
vs. No	656	92 (14.0)	564 (86.0)		
**Personal experience of breast screening**					Overall: **.013**
Taken part	221	9 (4.1)	212 (95.9)	6.12, 1.37 to 27.33	**.018**
Invited, never taken part	34	6 (17.6)	28 (82.4)	0.99, 0.21 to 4.61	.986
vs. Not eligible or not invited	445	84 (18.9)	361 (81.1)		
**Belief that screening is almost always a good idea**					
Yes	660	81 (12.3)	579 (87.7)	9.08, 3.77 to 21.88	**<.0005**
vs. No or not sure	40	18 (45.0)	22 (55.0)		
**Awareness of volcano news**					
Yes	505	54 (10.7)	451 (89.3)	1.34, 0.74 to 2.41	.335
vs. No or not sure	195	45 (23.1)	150 (76.9)		

For peer review only

For peer review only - http://bmjopen.bmj.com/site/about/guidelines.xhtml

**Awareness of election news**					
Yes	424	47 (11.1)	377 (88.9)	1.42, 0.82 to 2.46	.208
vs. No or not sure	276	52 (18.8)	224 (81.2)		
**General level of trust in participants' GPs**					Overall: .025
A lot	339	31 (9.1)	308 (90.9)	1.55, 0.45 to 5.29	.487
Somewhat	221	44 (19.9)	177 (80.1)	0.76, 0.23 to 5.67	.663
A little	109	16 (14.7)	93 (85.3)	2.48, 0.69 to 8.90	.163
vs. Not at all	31	8 (25.8)	23 (74.2)		
**General level of trust in the NHS**					Overall: .007
A lot	344	34 (9.9)	310 (90.1)	1.16, 0.29 to 4.64	.832
Somewhat	257	42 (16.3)	215 (83.7)	0.70, 0.18 to 2.79	.614
A little	76	18 (23.7)	58 (76.3)	0.27, 0.06 to 1.11	.068
vs. Not at all	23	5 (21.7)	18 (78.3)		
**Frequency of worry about breast cancer**					Overall: **.028**
Very often	39	3 (7.7)	36 (92.3)	3.00, 0.72 to 12.51	.132
Often	43	5 (9.3)	39 (90.7)	2.95, 0.85 to 10.26	.089
Sometimes	177	20 (11.3)	157 (88.7)	2.59, 1.31 to 5.15	**.006**
Occasionally	231	27 (11.7)	204 (88.3)	2.15, 1.15 to 4.02	**.016**
vs. Never	210	45 (21.4)	165 (78.6)		

Table H – Number of participants with missing data for each variable of interest

**Measure**	**Total (n=1,894)**
Awareness of the news about breast screening	0
Recruitment wave	0
Gender	0
Ethnicity	8
Marital status	0
Highest level of education	17
Social class grade	0
Employment status	0
Area type	0
Personal diagnosis of cancer	33
Personal experience of breast screening	33
Belief that screening is almost always a good idea	**0**
Awareness of the news about the volcanic eruption	0
Awareness of the news about the local elections	0
General level of trust in participants' GPs	67
General level of trust in the NHS	19
Frequency of worry about breast cancer	22
Breast screening intentions for next invitation	9

For peer review only - http://bmjopen.bmj.com/site/about/guidelines.xhtml

For peer review only

STROBE Statement—Checklist of items that should be included in reports of ***cross-sectional studies***

	Item No	Recommendation	Page number
**Title and abstract**	1	(*a*) Indicate the study's design with a commonly used term in the title or the abstract	1
		(*b*) Provide in the abstract an informative and balanced summary of what was done and what was found	2
**Introduction**			
Background/rationale	2	Explain the scientific background and rationale for the investigation being reported	3-4
Objectives	3	State specific objectives, including any prespecified hypotheses	4
**Methods**			
Study design	4	Present key elements of study design early in the paper	4
Setting	5	Describe the setting, locations, and relevant dates, including periods of recruitment, exposure, follow-up, and data collection	4
Participants	6	(*a*) Give the eligibility criteria, and the sources and methods of selection of participants	4-5
Variables	7	Clearly define all outcomes, exposures, predictors, potential confounders, and effect modifiers. Give diagnostic criteria, if applicable	5-7
Data sources/ measurement	8*	For each variable of interest, give sources of data and details of methods of assessment (measurement). Describe comparability of assessment methods if there is more than one group	5-7
Bias	9	Describe any efforts to address potential sources of bias	4-8
Study size	10	Explain how the study size was arrived at	5
Quantitative variables	11	Explain how quantitative variables were handled in the analyses. If applicable, describe which groupings were chosen and why	7-8
Statistical methods	12	(*a*) Describe all statistical methods, including those used to control for confounding	7-8
		(*b*) Describe any methods used to examine subgroups and interactions	7-8
		(*c*) Explain how missing data were addressed	8
		(*d*) If applicable, describe analytical methods taking account of sampling strategy	N/A
		(*e*) Describe any sensitivity analyses	N/A
**Results**			
Participants	13*	(a) Report numbers of individuals at each stage of study—eg numbers potentially eligible, examined for eligibility, confirmed eligible, included in the study, completing follow-up, and analysed	8-13
		(b) Give reasons for non-participation at each stage	8
		(c) Consider use of a flow diagram	N/A
Descriptive data	14*	(a) Give characteristics of study participants (eg demographic, clinical, social) and information on exposures and potential confounders	8-9
		(b) Indicate number of participants with missing data for each variable of interest	P8 (appendix 2)
Outcome data	15*	Report numbers of outcome events or summary measures	8-13
Main results	16	(*a*) Give unadjusted estimates and, if applicable, confounder-adjusted estimates and their precision (eg, 95% confidence interval). Make clear which confounders were adjusted for and why they were included	11-13

For peer review only - http://bmjopen.bmj.com/site/about/guidelines.xhtml

		(*b*) Report category boundaries when continuous variables were categorized	11-13
		(*c*) If relevant, consider translating estimates of relative risk into absolute risk for a meaningful time period	N/A
Other analyses	17	Report other analyses done—eg analyses of subgroups and interactions, and sensitivity analyses	N/A
**Discussion**			
Key results	18	Summarise key results with reference to study objectives	8-10
Limitations	19	Discuss limitations of the study, taking into account sources of potential bias or imprecision. Discuss both direction and magnitude of any potential bias	15-16
Interpretation	20	Give a cautious overall interpretation of results considering objectives, limitations, multiplicity of analyses, results from similar studies, and other relevant evidence	16
Generalisability	21	Discuss the generalisability (external validity) of the study results	14-16
**Other information**			
Funding	22	Give the source of funding and the role of the funders for the present study and, if applicable, for the original study on which the present article is based	16

*Give information separately for exposed and unexposed groups.

Note: An Explanation and Elaboration article discusses each checklist item and gives methodological background and published examples of transparent reporting. The STROBE checklist is best used in conjunction with this article (freely available on the Web sites of PLoS Medicine at http://www.plosmedicine.org/, Annals of Internal Medicine at http://www.annals.org/, and Epidemiology at http://www.epidem.com/). Information on the STROBE Initiative is available at www.strobe-statement.org.

For peer review only

BMJ Open

Public awareness of and responses to media coverage of invitation errors in the Breast Screening Programme in England: A cross-sectional population survey

Journal:	*BMJ Open*
Manuscript ID	bmjopen-2018-028040.R2
Article Type:	Research
Date Submitted by the Author:	19-Jun-2019
Complete List of Authors:	Ghanouni, Alex; University College London, Research Department of Behavioural Science and Health<br>von Wagner, Christian; UCL, Research Department of Behavioural Science and Health<br>Waller, Jo; UCL, Research Department of Behavioural Science and Health
**Primary Subject Heading**:	Public health
Secondary Subject Heading:	Communication
Keywords:	Organisation of health services < HEALTH SERVICES ADMINISTRATION & MANAGEMENT, JOURNALISM (see Medical Journalism), PUBLIC HEALTH, Breast imaging < RADIOLOGY & IMAGING

SCHOLARONE™
Manuscripts

FULL TITLE PAGE

Title: Public awareness of and responses to media coverage of invitation errors in the Breast Screening Programme in England: A cross-sectional population survey

Manuscript type: Research article

Authors:

Alex Ghanouni[a] a.ghanouni@ucl.ac.uk

Christian von Wagner[a] c.wagner@ucl.ac.uk

Jo Waller[ab] j.waller@ucl.ac.uk | Tel: +44 (0)20 7679 5958 | Fax: +44 (0)20 7679 8354

Affiliations:

[a]Research Department of Behavioural Science and Health, University College London, Gower Street, London, WC1E 6BT, United Kingdom

[b]Corresponding author

For peer review only - http://bmjopen.bmj.com/site/about/guidelines.xhtml

For peer review only

ABSTRACT

Objectives: In May 2018, the British Health Secretary announced the ‘serious failure’ that 450,000 women had missed out on invitations to breast screening in England, leading to extensive media coverage. This study measured public awareness of the story and tested for associated factors (e.g. educational level and trust in the NHS).

Design: A computer-assisted face-to-face survey in June 2018.

Setting: Participants completed the survey in their homes.

Participants: Males and females aged 16 years or older in England.

Primary and secondary outcome measures: Awareness of aspects of the media coverage and reported statistics. Other data included demographics (e.g. ethnicity), awareness of unrelated contemporaneous news stories, trust in participants’ GPs and the NHS, and (among women) worry about breast cancer and future breast screening intentions.

Results: Descriptive statistics showed that 67% of 1,894 participants reported being aware of the media coverage. Regression analyses showed that those who were aware of other news stories, were white British, and had a higher level of education or social class grade were more likely to be aware. In contrast, only 36% correctly identified at least one of two headline statistics. This study did not find evidence that awareness was negatively associated with trust in participants’ GPs or the NHS, breast cancer worry or future breast cancer screening intentions.

Conclusions: Awareness of the breast screening news story was high but recall of reported statistics was much lower: the public may have retained only the gist of quantitative information. Associations between story awareness and attitudes or behaviour were not apparent.

Keywords: Breast imaging; Journalism; Organisation of health services; Public Health

For peer review only

For peer review only - http://bmjopen.bmj.com/site/about/guidelines.xhtml

Strengths and limitations of this study:

- This study builds on previous research on media coverage around public health concerns by measuring levels of awareness among the general public and testing for characteristics associated with awareness.
- The survey was carried out shortly after media coverage of the announcement began, when awareness and knowledge were likely to be at their highest.
- Associations between awareness of media coverage and e.g. greater worry about breast cancer and lower trust in the NHS were not apparent but Type II error cannot be excluded.
- Tests for associations between awareness of media coverage and screening behaviour were based on intended future uptake; actual uptake may differ.

For peer review only

INTRODUCTION

On 2nd May 2018, the Health Secretary in Great Britain, Jeremy Hunt, made an unanticipated statement to the House of Commons regarding *“a serious failure…in the national Breast Screening Programme”*. Mr Hunt stated that since 2009, *“a computer algorithm failure”* had resulted in approximately 450,000 women not being invited to their final regular breast screening appointment (i.e. when they were aged 68 to 71 years). He indicated to the House that *“[the] current best estimate based on statistical modelling…is that there may be between 135 and 270 women who had their lives shortened as a result”* and that women affected *"will automatically be sent an invitation to a catch-up screening"*.[1] News of this statement was reported extensively in the national media (e.g. [2-4]) and prompted a volume of follow-up commentary from academics aiming to add context to this story. For example, some raised arguments that breast screening has no effect on all-cause mortality and risks resulting in overdiagnosis.[5-6]

Awareness of health-related media coverage is likely to be very high among academics and clinicians who are professionally invested in the topic. However, research is lacking on the prevalence of awareness of this type of news among the general public. In the absence of empirical data, it might be hypothesised to be either high (e.g. because mainstream media coverage has an extremely wide reach) or generally low (because members of the public are more focused on their personal priorities or do not have a specific interest in health news).

For peer review only - http://bmjopen.bmj.com/site/about/guidelines.xhtml

Levels of public awareness of health media coverage is significant because it represents the proportion of people who may be influenced by it: previous research has found that media coverage of cancer-related stories in the United Kingdom has appreciable public health implications. For example, there is evidence that the cervical cancer diagnosis and death of a young female celebrity, Jade Goody, influenced women's cervical cancer screening decisions and temporarily increased uptake and diagnoses of high-grade cervical neoplasia.[7-10] Similarly, uptake of the colorectal screening programme increased following coverage of the United Kingdom Flexible Sigmoidoscopy Screening Trial.[11-12] This coverage often contained elements likely to be perceived highly favourably by the general public such as the fact that it was a five-minute, one-off test that could save thousands of lives. In addition, the word "breakthrough" was often featured.[13-16] Comparable findings have been reported by studies of pre-planned media messages such as Public Health England's 'Be Clear on Cancer' campaigns, which aim to increase cancer symptom awareness. These were associated with an increase in symptomatic attendance at General Practices and referrals to secondary care.[17-19]

In these cases, media coverage was associated with an increase in healthcare usage. However, news about an error in the screening programme may have had adverse effects, such as diminishing trust in the National Health Service (with corresponding negative implications for help-seeking), more frequent worry about breast cancer, and being less inclined to have breast screening in future. To our knowledge, this possibility has not been investigated by research to date.

This study surveyed awareness of the coverage shortly after the announcement (when conscious recall was likely to be highest) in a large, sociodemographically diverse sample of the general public. In order to make a more complete assessment of this awareness, we also measured knowledge of the relevant statistics most commonly reported as part of the story (i.e. the number of women estimated to have missed an invitation and to have had their lives shortened) since these were a key factor in making a personal assessment of the scale and severity of the invitation errors. We also recognised that people's concerns about the initial coverage may have been moderated by follow-up commentary noting issues around overdiagnosis and all-cause mortality in breast screening. We used these measures to conduct an exploratory analysis of variables associated with awareness of the media coverage, including education, gender, and awareness of other news stories that were reported around the same time. We also tested the hypotheses that awareness of the breast

For peer review only - http://bmjopen.bmj.com/site/about/guidelines.xhtml

screening media coverage would be associated with lower trust in participants' GPs and the NHS and (in women) more frequent worry about breast cancer and being less likely to intend to participate in breast screening in future.

METHODS

Design

Institutional ethical approval was obtained (registration number: 2951/006). A market research agency (Kantar TNS UK) collected data in two waves of sampling between 6th and 10th June 2018 (i.e. less than six weeks after the initial news story. The survey questions formed one module within a weekly face-to-face computer-assisted omnibus survey on a wider range of topics. Random location sampling was used to identify target households based on the 2011 Census and Postcode Address File. At each location, quotas were set with the aim of achieving national representativeness based on working status, children in the household, gender, and age.

The full survey is included in Appendix 1. Participants were initially shown a computer screen with text introducing the study and asking for their consent to participate. They were also given an information card containing debrief text and directions to further information about breast screening.

Participants

Eligible participants were all males and females in England aged 16 years or older who consented to take part in this module of the survey. The sample includes women eligible for breast screening (i.e. aged 47 to 73 years) and also members of the general population (males and females aged 16 years or older) since it was hypothesised that awareness of the story had the potential to negatively affect perceptions of other health services, irrespective of whether participants were affected directly. Sample size was based on budgetary constraints and the number of participants who could be approached no more than six weeks after the initial news story.

Patient and public involvement

Since the results of the study were expected to be highly time-sensitive, rapid data collection was prioritised over involving patients and the public in the design and conduct of the study. In order to

For peer review only - http://bmjopen.bmj.com/site/about/guidelines.xhtml

minimise data protection issues, survey responses were received by the research team in anonymised format, meaning that it is not possible to disseminate study results to participants.

Measures

Demographics:

General background information included participants' self-reported age (in years), gender, ethnic origin, marital status, education, social class grade,[20] employment status, and urban or rural area type.

Cancer and breast screening experience, and attitudes towards screening:

Participants were asked whether they had been diagnosed with any of several types of cancer themselves. Women aged 47 years or older were also asked if they had ever been i) invited to and ii) participated in the Breast Screening Programme.

Participants were asked about their attitudes towards screening via a previously used question,[21] *"routine screening means testing healthy people to find cancer before they have any symptoms. Do you think routine cancer screening tests for healthy people are almost always a good idea?"*. Response options were *"yes"*, *"no"*, and *"not sure"*.

Awareness of the breast screening news story:

Participants were asked to read a brief summary of the story (see Appendix 1, Q7), the main details of which were derived from the primary story on the topic on the BBC news website.[4] This was followed by the question, *"do you recall seeing or hearing anything about this news story before now?"*. Response options were *"yes"*, *"no"*, and *"not sure"*.

It was anticipated that directions of associations with awareness may depend on the specific parts of the story of which participants were aware. Consequently, participants who reported being aware of the main news story were also asked about their awareness of issues relating to all-cause mortality and overdiagnosis using two further summaries (see Appendix 1, Q14 and Q15), derived from two sources.[5-6]

For peer review only - http://bmjopen.bmj.com/site/about/guidelines.xhtml

Questions for assessing awareness were the same as previous. Participants reporting awareness of the news story were also asked where they saw or heard it and whether they discussed or shared it with anyone else. They were also asked two questions on the key statistics reported based on the following summaries:

"The Health Secretary, Jeremy Hunt, gave an estimate of the number of women who had failed to get invitations since 2009."

"The Health Secretary also gave an estimate, based on computer modelling, of the number of women who may have had their lives shortened."

For both, the question was *"which of the following do you think is the estimate that he gave?"*. For the first question, response options consisted of the true estimate (450,000) and three alternatives that were orders of magnitude higher or lower (4,500, 45,000, and 4,500,000). Similarly, response options for the second question consisted of the correct answer (between 135 and 270) and alternatives that were either an order of magnitude higher (1,350 and 2,700), lower (13 and 27), or both higher and lower (13 and 2,700). Response order was presented in one of two different ways for each participant (determined at random) to reduce potential order effects.

Awareness of news stories unrelated to breast screening:

Awareness of other news stories was measured by asking participants to read two further summaries (one on a volcano eruption in Hawaii; one on local council elections in England; see Appendix 1, Q19 and Q20). This was followed by the same measure of awareness as in previous questions. Main details were derived from the primary stories on the BBC news website.[22-23] These two stories were selected for comparison because they were reported around the same time and also consisted of specific, definable events.

Trust in health services:

Participants were asked two questions based on previously used items,[24-25] *"in general, how much do you trust…"* i) *"…your general practitioner?"* and ii) *"…the NHS?"*. Response options for both were *"not at all"*, *"a little"*, *"somewhat"*, *"a lot"*, and *"not sure"*.

Frequency of breast cancer worry:

For peer review only - http://bmjopen.bmj.com/site/about/guidelines.xhtml

Breast cancer worry (among women) was measured using an item based on one previously used,[26] *"how often do you worry about your chances of getting breast cancer yourself?"*. Response options were, *"never"*, *"occasionally"*, *"sometimes"*, *"often"*, *"very often"*, *"not sure"*, and *"prefer not to say"*.

Breast screening intentions:

Women aged 16 to 69 years were asked, *"do you think you will go for breast screening when you are next offered it?"*. Response options were *"yes, definitely"*, *"yes, probably"*, *"no, probably not"*, and *"no, definitely not"*.

Analysis

Participant characteristics and awareness about the news stories are reported using descriptive statistics. Responses of *"prefer not to say"* were excluded, as were responses of *"not sure"* for ordinal variables. Other responses of *"not sure"*, were grouped with *"no"*. Ethnicity was dichotomised into *"white British"* and *"other groups"*; social class grades were grouped into *"A or B"*, *"C1"*, *"C2"*, and *"D or E"*. For education, *"trade apprenticeships"* were grouped with *"other qualifications"*. Responses to measures of invitations to and participation in breast screening were coded into *"not eligible or not invited"*, *"invited, never taken part"*, and *"taken part"*.

One exploratory regression model tested for variables potentially associated with whether people responded to the survey. Three exploratory regression models tested for variables potentially associated with i) awareness of the breast screening news; and stating correctly the number of women who were ii) not invited for screening and iii) estimated to have had their lives shortened. A further four regression models tested the null hypotheses that awareness of the breast screening news story was not associated with trust in iv) participants' GPs and v) the NHS in the whole sample; and vi) frequency of worry about breast cancer and vii) intentions to participate in breast screening in future among women aged 70 years or less, after adjusting for covariates.

For the model assessing variables associated with responding to the questionnaire, the main variables of interest were recruitment wave, gender, ethnicity, marital status, social class grade, employment status, area type, and age (since these were the variables where data were available for both participants and non-participants). For the four main exploratory models and hypothesis testing models, independent variables were as above with the addition of other available measures (listed in

For peer review only - http://bmjopen.bmj.com/site/about/guidelines.xhtml

tables) where multi-collinearity was not an appreciable issue (i.e. Variance Inflation Factors <10). Age was included in models as either a continuous variable or divided into age groups (where a Box-Tidwell procedure found evidence that the assumption of linearity was not met; p<.05). Frequency of worry about breast cancer was also included in the model of future breast screening intentions.

For models testing hypotheses, responses on measures of awareness of the breast screening story were coded into a single nominal variable with five levels: 1) *"unaware of the story"*, 2) *"aware of the main story only"*, 3) *"aware of the main story and all-cause mortality follow-up commentary"*,4) *"aware of the main story and overdiagnosis follow-up commentary"*, 5) *"aware of the main story and both follow-up commentaries"*.

Ordinal logistic regression was attempted in the first instance where dependent variables were ordinal. Tests of parallel lines suggested that the assumption of proportional odds was generally not met (p<.0005) and there were few cases in some cells. Hence, dependent variables were dichotomised and binary logistic regression was used. Participants with missing data on variables of interest were not included in models.

RESULTS

Participant characteristics

2,681 participants began the survey module. 787 (29.4%) opted out, leaving 1,894 participants who provided data. Mean age was 50.8 years (standard deviation: 20.5). Characteristics are described in Appendix 2 (Table A). Response to the survey module questions was associated with all variables in the model, except for area type (Appendix 2, Table B). Participants of the omnibus survey approached were more likely to respond to this survey module if they were invited in wave 1 (vs. wave 2), female (vs. male), white British (vs. other groups), married, living as a couple, or widowed, divorced or separated (vs. single), in higher social class grades (vs. grades D or E), working (vs. not working), and younger.

Awareness of news stories, sources of information, and variables associated with awareness of the breast screening media coverage

For peer review only - http://bmjopen.bmj.com/site/about/guidelines.xhtml

1,264/1,894 (66.7%) reported being aware of the main news story (Appendix 2, Table A) and relatively few reported being aware of follow-up commentaries: 438/1,264 (34.7%) and 367/1,264 (29.0%) recognised the commentaries on all-cause mortality and overdiagnosis, respectively. 250/1,264 (19.8%) were aware of both. 971/1,264 (76.8%) and 271/1,264 (21.4%) encountered the story on television and radio, respectively (participants could select more than one). 169/1,264 (13.4%) and 134/1,264 (10.6%) encountered the story in print newspapers and online news websites (Appendix 2, Table C). Other news sources were used relatively rarely e.g. 68/1,264 (5.4%) heard the story from social media websites. 450/1,264 (35.6%) reported discussing or sharing the story with someone else.

Participants were more likely to be aware of the story if they were aware of either of the other two news stories. Awareness of the three stories was highly interrelated: 824/1,894 participants (43.5%) were aware of all three news stories and a further 196/1,894 (10.3%) reported not being aware of any. Only 323/1,894 (17.1%) were aware of just one of the three stories and only 106/1,894 participants (5.6%) were aware of the news about breast screening, specifically. Participants were also more likely to be aware of the breast screening news story if they were white British, older, had higher levels of education or social class grade. Participants were less likely to be aware if they believed that screening was almost always a good idea. All other p-values were ≥.207 (Table 1).

Awareness of statistics from the breast screening media coverage and variables associated with awareness among participants who reported being aware of the story

Only 233 (18.4%) of the 1,264 participants who reported being aware of the story correctly recognised the number of women who had not been invited and only 268 (21.2%) correctly recognised the estimated number of women who had their lives shortened. 809 (64.0%) did not correctly identify either statistic and only 3.6% correctly identified both (Table 2). The model testing for demographic and psychological variables associated with correctly identifying either set of statistics found only weak evidence against the null hypothesis for all characteristics (p-values were ≥.087 and ≥.062 in the respective models; data not shown).

Awareness of media coverage and participants' trust their GPs and the NHS

For peer review only - http://bmjopen.bmj.com/site/about/guidelines.xhtml

In both these models, there was only weak evidence against the null hypothesis. Table 3 shows the main results of binary logistic regression models consisting of 1,746 participants (p=.729 and .290). Full results of the model are presented in Appendix 2 (Table D and Table E).

Awareness of media coverage and frequency of worry about breast cancer

Table 4 shows that there was only weak evidence against the null hypothesis (n=700; p=.198). Full results are included in Appendix 2 (Table F).

Awareness of media coverage and future breast screening intentions

Table 5 shows that there was only weak evidence against the null hypothesis for this analysis (n=700; p=.108). Full results are included in Appendix 2 (Table G).

Numbers of participants with missing data for each variable are shown in Appendix 2 (Table H).

For peer review only

Table 1 – Full results of the binary logistic regression model testing for variables associated with awareness of the breast screening news story

		Aware vs. Not aware of the breast screening story (or not sure): n (%)		Adjusted OR, 95% CI	p-value
**Characteristic**	**Total (n=1,792)**	**Not aware/sure (n=587; 32.8%)**	**Aware (n=1,205; 67.2%)**	**Aware of the screening story (vs. Not aware or not sure)**	
**Recruitment wave**					
Wave 2: 20-26th June	570	185 (32.5)	385 (67.5)	1.02, 0.79 to 1.31	.907
vs. Wave 1: 6-10th June	1,222	402 (32.9)	820 (67.1)		
**Age**					Overall: **<.0005**
65+	549	111 (20.2)	438 (79.8)	7.77, 4.52 to 13.38	**<.0005**
55-64	252	53 (21.0)	199 (79.0)	6.75, 3.92 to 11.63	**<.0005**
45-54	241	47 (19.5)	194 (80.5)	7.70, 4.56 to 13.00	**<.0005**
35-44	248	88 (35.5)	160 (64.5)	3.60, 2.22 to 5.84	**<.0005**
25-34	275	142 (51.6)	133 (48.4)	2.00, 1.27 to 3.14	**.003**
vs. 16-24	227	146 (64.3)	81 (35.7)		
**Gender**					
Male	771	234 (30.4)	537 (69.6)	1.00, 0.74 to 1.35	.999
vs. Female	1,021	353 (34.6)	668 (65.4)		
**Ethnicity**					
White British	1,491	415 (27.8)	1,076 (72.2)	3.00, 2.20 to 4.09	**<.0005**
vs. Other groups	301	172 (57.1)	129 (42.9)		
**Marital status**					Overall: .914
Married/Living as a couple	985	279 (28.3)	706 (71.7)	1.07, 0.78 to 1.47	.672
Widowed/Divorced/Separated	354	84 (23.7)	270 (76.3)	1.06, 0.70 to 1.60	.792
vs. Single	453	224 (49.4)	229 (50.6)		
**Highest level of education**					Overall: **.001**
Graduate level/Above	501	131 (26.1)	370 (73.9)	2.08, 1.34 to 3.23	**.001**
A-levels/AS levels/Equivalents	448	162 (36.2)	286 (63.8)	1.80, 1.19 to 2.73	**.006**
GCSEs/Equivalents	440	156 (35.5)	284 (64.5)	1.36, 0.92 to 2.00	.120
Trade apprenticeships/Other	89	39 (43.8)	50 (56.2)	0.75, 0.42 to 1.32	.316
vs. No formal qualifications	314	99 (31.5)	215 (68.5)		
**Social class grade**					Overall: **<.0005**
Grade A or B	326	53 (16.3)	273 (83.7)	2.44, 1.59 to 3.73	**<.0005**
Grade C1	511	165 (32.3)	346 (67.7)	1.41, 1.02 to 1.95	**.037**
Grade C2	394	142 (36.0)	252 (64.0)	1.13, 0.81 to 1.58	.469
vs. Grade D or E	561	227 (40.5)	334 (59.5)		
**Employment status**					
Working	823	287 (34.9)	536 (65.1)	0.91, 0.68 to 1.22	.909
vs. Not working	969	300 (31.0)	669 (69.0)		
**Area type**					
Urban	1,458	476 (32.6)	982 (67.4)	1.21, 0.90 to 1.64	.207
vs. Rural	334	111 (33.2)	223 (66.8)		
**Personal diagnosis of cancer**					
Yes	150	34 (22.7)	116 (77.3)	1.18, 0.74 to 1.86	.490
vs. No	1,642	553 (33.7)	1,089 (66.3)		
**Personal experience of breast screening**					Overall: .552
Taken part	425	90 (21.2)	335 (78.8)	0.92, 0.60 to 1.41	.705
Invited, never taken part	55	13 (23.6)	42 (76.4)	0.66, 0.32 to 1.39	.276
vs. Not eligible or not invited	1,312	484 (36.9)	828 (63.1)		
**Belief that screening is almost always a good idea**					
Yes	1,649	547 (33.2)	1,102 (66.8)	0.59, 0.38 to 0.94	**.025**
vs. No or not sure	143	40 (28.0)	103 (72.0)		
**Awareness of volcano news**					
Yes	1,367	325 (23.8)	1,042 (76.2)	3.14, 2.39 to 4.12	**<.0005**
vs. No or not sure	425	262 (61.6)	163 (38.4)		
**Awareness of election news**					
Yes	1,138	292 (25.7)	846 (74.3)	1.37, 1.06 to 1.75	**.014**
vs. No or not sure	654	295 (45.1)	359 (54.9)		
**General level of trust in the NHS**					Overall: .485
A lot	969	308 (31.8)	661 (68.2)	0.59, 0.29 to 1.18	.132

For peer review only

For peer review only - http://bmjopen.bmj.com/site/about/guidelines.xhtml

Somewhat	599	193 (32.2)	406 (67.8)	0.63, 0.31 to 1.27	.196
A little	169	69 (40.8)	100 (59.2)	0.58, 0.27 to 1.25	.166
vs. Not at all	55	17 (30.9)	38 (69.1)		

Table 2 – Descriptive statistics of participants' responses about key statistics in the breast screening media coverage; correct responses were "450,000" and "135-270"

	n (% of total; 95% CI) (n=1,264)					
**Number of women who did not**	**Number of women who may have had their life shortened. Between…**					
**receive their final invitation…**	*135 - 270*	*13 - 27*	*13 - 2,700*	*1,350 - 2,700*	*Not sure*	*Total*
450,000	46 (3.6)	6 (0.5)	79 (6.3)	71 (5.6)	31 (2.5)	233 (18.4)
4,500	68 (5.4)	20 (1.6)	28 (2.2)	22 (1.7)	30 (2.4)	168 (13.3)
45,000	130 (10.3)	22 (1.7)	76 (6.0)	86 (6.8)	54 (4.3)	368 (29.1)
4,500,000	3 (0.2)	1 (0.1)	10 (0.8)	20 (1.6)	4 (0.3)	38 (3.0)
Not sure	21 (2.1)	5 (0.4)	15 (1.2)	12 (0.9)	404 (32.0)	457 (36.2)
Total	268 (21.2)	54 (4.3)	208 (16.5)	211 (16.7)	523 (41.4)	

Table 3 – Testing for an association between awareness of the breast screening media coverage and trust in i) participants' GPs and ii) the NHS*

**General level of trust in participants' GPs**		**A lot vs. Not at all; a little; somewhat: n (%)**		**Adjusted OR, 95% CI**	**p-value**
**Characteristic**	**Total (n=1,746)**	**Less than a lot (n=781; 44.7%)**	**A lot (n=965; 55.3%)**	**A lot (vs. Less than a lot)**	
**Screening story awareness**					Overall: .729
Aware of the main story and both follow-up commentaries	238	98 (41.2)	140 (58.8)	1.10, 0.74 to 1.64	.653
Aware of the main story and overdiagnosis follow-up	172	66 (38.4)	106 (61.6)	1.31, 0.85 to 2.03	.218
Aware of the main story and all-cause mortality follow-up	107	49 (45.8)	58 (54.2)	1.21, 0.73 to 2.02	.459
Aware of the main story only	655	280 (42.7)	375 (57.3)	1.17, 0.88 to 1.57	.283
vs. Unaware of the story	574	288 (50.2)	286 (49.8)		
**General level of trust in the NHS**		**A lot vs. Not at all; a little; somewhat: n (%)**		**Adjusted OR, 95% CI**	**p-value**
**Characteristic**	**Total (n=1,746)**	**Less than a lot (n=803; 46.0%)**	**A lot (n=943; 54.0%)**	**A lot (vs. Less than a lot)**	
**Screening story awareness**					Overall: .290
Aware of the main story and both follow-up commentaries	238	102 (42.9)	136 (57.1)	0.87, 0.59 to 1.30	.503
Aware of the main story and overdiagnosis follow-up	172	76 (44.2)	96 (55.8)	0.78, 0.51 to 1.21	.267
Aware of the main story and all-cause mortality follow-up	107	57 (53.3)	50 (46.7)	0.58, 0.35 to 0.97	.039
Aware of the main story only	655	299 (45.6)	356 (54.4)	0.81, 0.60 to 1.09	.160
vs. Unaware of the story	574	269 (46.9)	305 (53.1)		

*Results are adjusted based on the following covariates: Recruitment wave, Age (Age group in the model of trust in the NHS), Gender, Ethnicity, Marital status, Highest level of education, Social class grade, Employment status, Area type, Personal diagnosis of cancer, Personal experience of breast screening, Belief that screening is almost always a good idea, Awareness of volcano news, Awareness of election news, General level of trust in the NHS (General level of trust in participants' GPs in the model of trust in the NHS). Full results of the model are reported in the Appendix

For peer review only

For peer review only - http://bmjopen.bmj.com/site/about/guidelines.xhtml

Table 4 – Testing for an association between awareness of the breast screening media coverage and frequency of worry about breast cancer*

		Never; occasionally vs. Sometimes; often; very often: n (%)		Adjusted OR, 95% CI	p-value
Characteristic	Total (n=700)	Never; occasionally (n=441; 63.0%)	Sometimes; often; very often (n=259; 37.0%)	Sometimes; often; very often (vs. Never; occasionally)	
**Screening story awareness**					Overall: .198
Aware of the main story and both follow-up commentaries	88	65 (73.9)	23 (26.1)	0.85, 0.46 to 1.58	.614
Aware of the main story and overdiagnosis follow-up	63	42 (66.7)	21 (33.3)	1.05, 0.55 to 2.01	.878
Aware of the main story and all-cause mortality follow-up	36	25 (69.4)	11 (30.6)	1.10, 0.49 to 2.49	.819
Aware of the main story only	270	153 (56.7)	117 (43.3)	1.49, 0.98 to 2.25	.062
vs. Unaware of the story	243	156 (64.2)	87 (35.8)		

*Results are adjusted for covariates: Recruitment wave, Age, Ethnicity, Marital status, Highest level of education, Social class grade, Employment status, Area type, Personal diagnosis of cancer, Personal experience of breast screening, Belief that screening is almost always a good idea, Awareness of volcano news, Awareness of election news, General level of trust in participants' GPs, General level of trust in the NHS, Breast screening intentions for next invitation. Full results of the model are reported in the Appendix

Table 5 – Testing for an association between awareness of the breast screening media coverage and breast screening intentions*

		Yes, definitely vs. Yes, probably; no, probably not; no, definitely not: n (%)		Adjusted OR, 95% CI	p-value
Characteristic	Total (n=700)	No definite intention (n=99; 14.1%)	Definite intention (n=601; 85.9%)	Definite intention (vs. No definite intention)	
**Screening story awareness**					Overall: .108
Aware of the main story and both follow-up commentaries	88	10 (11.4)	78 (88.6)	2.01, 0.74 to 5.48	.172
Aware of the main story and overdiagnosis follow-up	63	4 (4.3)	59 (93.7)	2.66, 0.79 to 8.89	.113
Aware of the main story and all-cause mortality follow-up	36	6 (16.7)	30 (83.3)	0.66, 0.20 to 2.13	.486
Aware of the main story only	270	22 (8.1)	248 (91.9)	1.88, 0.99 to 3.57	.054
vs. Unaware of the story	243	57 (23.5)	186 (76.5)		

*Results are adjusted for covariates: Recruitment wave, Age group, Ethnicity, Marital status, Highest level of education, Social class grade, Employment status, Area type, Personal diagnosis of cancer, Personal experience of breast screening, Belief that screening is always a good idea, Awareness of volcano news, Awareness of election news, General level of trust in participants' GPs, General level of trust in the NHS, Frequency of worry about breast cancer. Full results of the model are reported in the Appendix

For peer review only - http://bmjopen.bmj.com/site/about/guidelines.xhtml

DISCUSSION

Previous studies have found evidence that media messages can increase usage of a range of healthcare services (e.g. [7-10, 12, 17-19]). Awareness of this story about errors in the breast screening programme was hypothesised to have the potential for a range of negative effects. However, the results of this study did not provide strong evidence against the null hypothesis for any associations tested. To the extent that these results reflect an absence of harms, this is reassuring: we did not find evidence that awareness of the story reduced trust in the NHS or participants' GPs, increased frequency of worry about breast cancer, or negatively affected future breast screening intentions. If this is the case, it may be partly attributable to the news story saying little to reduce the perceived benefits of breast screening itself, in contrast to media coverage of e.g. the independent review of breast cancer screening, which reported on the issue of overdiagnosis extensively.[27-28] Relatedly, the present study found that awareness was notably lower for follow-up commentaries on the shortcomings of breast screening, compared with the main story. In addition, the framing of the story may have been expected to reinforce the perceived benefits of screening by indicating that missing screening had negative consequences in terms of additional breast cancer deaths.

Population awareness of the breast screening news story was generally high. Television and radio were the main sources of information, broadly consistent with patterns of how most news is accessed, although the internet was used less often than observed in previous surveys.[29] Although no associations were found here, this finding is useful since it provides an estimate of the proportion of people who may be influenced by media coverage that does have positive or negative effects on health behaviour.[7-10, 12, 17-19] In the absence of this study, a plausible

For peer review only - http://bmjopen.bmj.com/site/about/guidelines.xhtml

rationale could have been found for why this estimate would be higher or lower than was shown to be the case.

Awareness of this story was related to awareness of other news stories, suggesting that an appreciable proportion of the population can be broadly dichotomised into those who are generally “news aware” and “news unaware”. These results do not suggest that a notable proportion of the public are aware of health news, specifically. In contrast to these findings, recall of the main statistics was markedly low and correct responses may be largely attributable to random guessing.[1] In some respects, this is surprising since the statistics were an integral part of the story and often part of headlines (e.g. [2-4, 30]) and may be a cause for concern: the number of women affected and estimated to have died as a result are important pieces of information in order for an individual to make a personal assessment of the scale and severity of the news. This finding may suggest that people either tend not to attend to or memorise this statistical information (meaning that they would not be able to factor it into their appraisal of the significance of the story) or they retain only the ‘gist’ of the statistics involved.[31] Awareness of the breast screening story was greater among those with higher levels of education and social class grade, those who were white British, and those who were older. Awareness of the breast screening news story was also lower among participants with positive attitudes towards screening (who may have been less likely to attend to a negative story).

---

[1] Participants were asked additional questions on the extent to which they trusted the statistics and their reasons for not trusting them (if applicable). However, since responses were highly suggestive of random guessing, no further analyses of these measures were attempted.

For peer review only - http://bmjopen.bmj.com/site/about/guidelines.xhtml

This study has limitations. Despite the large sample size and adjustment for a range of potentially confounding variables, the number of cases was relatively small in some cells (e.g. for having been invited to, but never participated in, screening and not believing, or being unsure whether, screening was almost always a good idea; Table 1) and some odds ratios were estimated with wide confidence intervals. Real associations may not have been detected (Type II error). In addition, our measures did not include a question on trust in the Breast Screening Programme, specifically, meaning that we could not test for associations with this outcome. Findings on screening uptake also relate only to anticipated future behaviour; future research could build on this study by assessing whether the announcement was followed by a decrease (or increase) in actual screening uptake. Although the response rate to this survey was higher than others of its type (e.g. 71% in the present study vs. 42% reported by Low et al.),[32] members of the public were also less likely to participate in the survey module based on a range of characteristics for which data were available. Results may be biased, insofar as responses differed based on these variables or unmeasured participant characteristics that may have reduced population-representativeness of the sample.

Conclusions

This study found that news of errors in the Breast Screening Programme in England had reached a large proportion of the general public and that those aware of the media coverage tended to be those aware of news stories in general. The proportion of people aware was also higher among those who had more education, were in a higher social class grade, or were older. In contrast, awareness of key statistics from the story was very low among participants aware of the story, even less than six weeks after the onset of the main media coverage. The results of this study did not provide evidence that media coverage had any effects on trust in

For peer review only - http://bmjopen.bmj.com/site/about/guidelines.xhtml

aspects of the health service among the general public, or worry about breast cancer or breast screening intentions among women. Future research should investigate possible effects of media coverage using objective measures of screening behaviour.

Contributors: AG, CVW, and JW conceived and designed the study. AG analysed the data. AG, CVW, and JW participated in the interpretation of results. AG, CVW, and JW drafted the manuscript, participated in critical revision, and approved the final version. Patients and the public were not involved in this study.

Funding: This work was supported by a programme grant from Cancer Research UK awarded to Prof Jane Wardle [C1418/A14134]. Dr Jo Waller is supported by a Career Development Fellowship from Cancer Research UK [C7492/A17219]. Cancer Research UK was not involved in the design of this study; the collection, analysis, or interpretation of the results; in the writing of the manuscript; or in the decision to submit for publication.

Exclusive Licence: I, the Submitting Author has the right to grant and does grant on behalf of all authors of the Work (as defined in the below author licence), an exclusive licence and/or a non-exclusive licence for contributions from authors who are: i) UK Crown employees; ii) where BMJ has agreed a CC-BY licence shall apply, and/or iii) in accordance with the terms applicable for US Federal Government officers or employees acting as part of their official duties; on a worldwide, perpetual, irrevocable, royalty-free basis to BMJ Publishing Group Ltd ("BMJ") its licensees and where the relevant Journal is co-owned by BMJ to the co-owners of the Journal, to publish the Work in BMJ Open and any other BMJ products and to exploit all rights, as set out in our licence.

Competing interests: None declared.

For peer review only - http://bmjopen.bmj.com/site/about/guidelines.xhtml

Patient consent: Obtained.

Data sharing: No additional data are available.

REFERENCES

1. House of Commons Hansard. Breast cancer screening https://hansard.parliament.uk/commons/2018-05-02/debates/BE9DB48A-C9FF-401B-AC54-FF53BC5BD83E/BreastCancerScreening (2018, accessed 18 October 2018).
2. Mail Online. 'They didn't deserve to lose their lives': Fury of husband whose wife died from breast cancer after 'colossal' NHS computer glitch which has seen 270 die and 450,000 miss screenings https://www.dailymail.co.uk/news/article-5682203/Hundreds-women-developed-breast-cancer-450-000-not-invited-screening.html (2018, accessed 18 October 2018).
3. The Guardian. Up to 270 women may have died after breast cancer screening IT error https://www.theguardian.com/society/2018/may/02/jeremy-hunt-to-launch-inquiry-into-450000-missed-breast-cancer-screenings (2018, accessed 18 October 2018).
4. BBC News. Breast screening error 'shortened up to 270 lives' – Hunt https://www.bbc.co.uk/news/health-43973652 (2018, accessed 18 October 2018).
5. Bewley S, Baum M, Hodkinson A, et al. Screening 'flaw'. The Times, 05 May 2018
6. Spiegelhalter D. Have 'up to 270 women died' by missing a breast screening appointment letter? https://medium.com/wintoncentre/have-up-to-270-women-died-by-missing-a-breast-screening-appointment-letter-756f74c4f56b (2018, 18 October 2018).
7. Lancucki L, Sasieni P, Patnick J, et al. The impact of Jade Goody's diagnosis and death on the NHS Cervical Screening Programme. J Med Screen 2012; 19:89–93.

For peer review only - http://bmjopen.bmj.com/site/about/guidelines.xhtml

8. MacArthur GJ, Wright M, Beer H, et al. Impact of media reporting of cervical cancer in a UK celebrity on a population-based cervical screening programme. J Med Screen 2011; 18:204–209.
9. Marlow LAV, Sangha A, Patnick J, et al. The Jade Goody Effect: Whose cervical screening decisions were influenced by her story? J Med Screen 2012; 19:184–188.
10. Casey GM, Morris B, Burnell M, et al. Celebrities and screening: a measurable impact on high-grade cervical neoplasia diagnosis from the “Jade Goody effect” in the UK. Br J Cancer 2013; 109:1192–1197.
11. Atkin WS, Edwards R, Kralj-Hans I, et al. Once-only flexible sigmoidoscopy screening in prevention of colorectal cancer: a multicentre randomised controlled trial. Lancet 2010; 375:1624–1633.
12. Lo S, Vart G, Snowball J, et al. The impact of media coverage of the Flexible Sigmoidoscopy Trial on English colorectal screening uptake. J Med Screening 2012; 19:83–88.
13. BBC News. Bowel cancer test could save many lives, study suggests http://news.bbc.co.uk/1/hi/health/8647103.stm (2010 11 June 2019).
14. Daily Express. Bowel cancer: New five-minute test could save thousands https://www.express.co.uk/news/uk/171872/Bowel-cancer-New-five-minute-test-could-save-thousands (2010, 11 June 2019)
15. The Telegraph. New cancer test cuts deaths by 40 per cent https://www.telegraph.co.uk/news/health/news/7638601/New-cancer-test-cuts-deaths-by-40-per-cent.html (2010, 11 June 2019)

For peer review only - http://bmjopen.bmj.com/site/about/guidelines.xhtml

16. The Guardian. Five-minute bowel cancer test could save thousands of lives, say scientists https://www.theguardian.com/society/2010/apr/28/bowel-cancer-breakthrough-claim (2010, 11 June 2019)
17. Moffat J, Bentley A, Ironmonger L, et al. The impact of national cancer awareness campaigns for bowel and lung cancer symptoms on sociodemographic inequalities in immediate key symptom awareness and GP attendances. Br J Cancer 2015; 112:S14–S21.
18. Bethune R, Marshall MJ, Mitchell SJ, et al. Did the "Be Clear on Bowel Cancer" public awareness campaign pilot result in a higher rate of cancer detection? Postgrad Med J 2013; 89:390–393.
19. Hughes-Hallett A, Browne D, Mensah E, et al. Assessing the impact of mass media public health campaigns. Be Clear on Cancer "blood in pee": a case in point. BJU Int 2016; 117:570–575.
20. National Readership Survey. Social Grade http://www.nrs.co.uk/nrs-print/lifestyle-and-classification-data/social-grade (n.d., accessed 18 October 2018).
21. Schwartz LM, Woloshin S, Fowler FJ, Welch HG. Enthusiasm for cancer screening in the United States. J Am Med Assoc. 2004;291(1):71–8.
22. BBC News. Local election results 2018: No clear winner as Labour and Tories neck and neck https://www.bbc.co.uk/news/uk-politics-44014076 (2018, 18 October 2018).
23. BBC News. Kilauea: Hawaii emergency declared over volcano eruption https://www.bbc.co.uk/news/world-us-canada-44001651 (2018, 18 October 2018).
24. Health Information National Trends Survey 2015 and 2017 https://hints.cancer.gov (2018, 18 October 2018).

For peer review only - http://bmjopen.bmj.com/site/about/guidelines.xhtml

25. Rutten LJF, Blake KD, Skolnick VG, et al. Data resource profile: The national Cancer Institute's Health Information National Trends Survey (HINTS). Int J Epidemiol 2019; in press.

26. Health Information Trends Survey 2003, 2005, and 2008 https://hints.cancer.gov (2018, 18 October 2018).

27. Independent UK Panel on Breast Cancer Screening. The benefits and harms of breast cancer screening: an independent review. Lancet 2012; 380:1778-1786.

28. BBC News. Breast screening advice updated amid controversy over tests https://www.bbc.co.uk/news/health-20121043 (2012, 18 October 2018).

29. OFCOM. News consumption in the UK https://www.ofcom.org.uk/research-and-data/tv-radio-and-on-demand/news-media/news-consumption (2018, 18 October 2018).

30. Sky News. Breast cancer screening failure 'shortened' up to 270 lives https://news.sky.com/story/inquiry-into-breast-cancer-screening-failures-to-be-launched-say-sky-sources-11355751 (2018, 18 October 2018).

31. Reyna VF, Brainerd CJ. Fuzzy-trace theory. An interim synthesis. Learn Individ Differ. 1997; 7:1–75.

32. Low EL, Waller J, Wardle J, Menon U. Experience of symptoms indicative of gynaecological cancers in UK women. Br J Cancer. 2013; 109:882-887.

For peer review only

For peer review only - http://bmjopen.bmj.com/site/about/guidelines.xhtml

APPENDIX 1 - SURVEY

[All Adults 16+ in England. Participants were shown the tablet screen and the following text was read out by interviewers]

Q.A In this part of the survey, I am going to ask you some questions related to health, including cancer, and recent news stories. These questions are asked on behalf of researchers from University College London. If you do not wish to answer a particular question during any part of this survey, you may refuse to answer and we will move to the next question. All your answers will be kept strictly confidential and you will be anonymous to the researchers.

The NHS currently offers breast cancer screening with mammography once every three years, to women aged between about 50 to 70 years in England.

Are you okay to continue with these questions?

1: Yes

2: No

As the questions can be perceived as sensitive, you can answer the questions on this machine yourself. I would now like to show you how to use the machine by going through a practice question with you.

[All Adults 16+ in England willing to continue. “Don’t know”/“Not sure”/“Prefer not to say” appeared at the top of the screen, out of view of participants, except for questions that participants completed themselves. Interviewers showed the screen to participants]

This is an example of a single-coded question

Q.B What is your favourite colour?

1: Red

2: Yellow

For peer review only

3: Blue

4: Green

Other colour (PEN -WRITE IN)

Don’t know

Refused

[All females aged 16-69 in England willing to continue. Interviewers handed tablets to participants and stepped away from viewing the screen]

Q.1 Do you think you will go for breast screening when you are next offered it? REMEMBER TO TAP OK TO CONTINUE

1: Yes, definitely

2: Yes, probably

3: No, probably not

4: No, definitely not

Not sure

Prefer not to say

[All females aged 47+ in England willing to continue]

Q.2 Have you ever been invited for breast screening before? If you’ve only ever been offered a mammogram to investigate symptoms separately to the screening programme, please respond ‘no’.

1: Yes

2: No

For peer review only

Not sure

Prefer not to say

[All females aged 47+ in England who have been invited for breast screening before]

Q.3 Have you ever been for breast screening as part of the screening programme?

1: Yes

2: No

Not sure

Prefer not to say

[All Adults 16+ in England willing to continue. This question allowed more than one response option. “None of the above”/”Prefer not to say” were mutually exclusive with other responses]

Q.4 Which of the following, if any, have you been diagnosed with? Please choose all that apply.

1: Bowel cancer

2: Lung cancer

3: Breast cancer

4: Cervical cancer

5: Prostate cancer

Other type of cancer - PEN WRITE IN

None of the above

Prefer not to say

For peer review only

[All Adults 16+ in England willing to continue. This question allowed more than one response option. “None of the above”/”Prefer not to say” were mutually exclusive with other responses]

Q.5 Has anyone you know ever been diagnosed with breast cancer? Please choose all that apply.

1: A close family member

2: Any other family member

3: A friend

4: A colleague

5: Any other person

Not sure

Prefer not to say

[All females aged 16+ in England willing to continue]

Q.6 How often do you worry about your chances of getting breast cancer yourself?

1: Never

2: Occasionally

3: Sometimes

4: Often

5: Very often

Not sure

Prefer not to say

Thank you for answering these questions - this is the end of this section for you.

For peer review only

[All Adults 16+ in England willing to continue. Participants handed the tablet back to the interviewer, who showed the screen and either read out or allowed participants to read subsequent questions]

Q.7 In May, it was reported that a computer algorithm failure had meant that a number of women did not receive invitations to their final routine breast cancer screening. The Health Secretary, Jeremy Hunt, said that women affected will be contacted by letter with an invitation for a catch-up screening test but some of the women who were not invited for their final appointment may have had their lives shortened.

Do you recall seeing or hearing anything about this news story before now?

1: Yes

2: No

Not sure

[All who recall seeing or hearing anything about this news story before now. This was a multiple choice question. The order of response options was randomised with “other websites” always following both “online news websites” and “social media websites”]

Q.8 Do you recall where you saw or heard this news story? Please choose all that apply.

1: Television

2: Print newspaper(s)

3: Radio

4: Online news websites

5: Social media websites

6: Other websites

For peer review only

7: Word of mouth

Other sources – PEN WRITE IN

Not sure

[All who recall seeing or hearing anything about this news story before now]

Q.9 Did you discuss or share the story with anyone else?

1: Yes

2: No

Not sure

[All who recall seeing or hearing anything about this news story before now. Participants were randomised to one of two orders of response options (1:1)]

Q.10 The Health Secretary, Jeremy Hunt, gave an estimate of the number of women who had failed to get invitations since 2009.

Which of the following do you think is the estimate that he gave?

1: 4,500 women

2: 45,000 women

3: 450,000 women

4: 4,500,000 women

Not sure

For peer review only

[All who recall seeing or hearing anything about this news story before now. Participants randomised to one of two orders of response options (1:1)]

Q.11 The Health Secretary also gave an estimate, based on computer modelling, of the number of women who may have had their lives shortened.

Which of the following do you think is the estimate that he gave?

1: Between 13 and 27 women

2: Between 135 and 270 women

3: Between 13 and 2,700 women

4: Between 1,350 and 2,700 women

Not sure

[All who gave an estimate in Q1 or Q11]

Q.12 How much did you trust these statistics when you heard them in the news?

1: Not at all

2: A little

3: Somewhat

4: A lot

Not sure

[All who do not trust the statistic]

Q.13 What were your reasons for not trusting these statistics when you heard them in the news?

PROBE: Any other reasons?

For peer review only

OPEN ENDED

[All who recall seeing or hearing anything about this news story before now]

Q.14 It was also reported that some health experts have said breast cancer screening can do “more harm than good” because they believe “breast screening…has no impact on all-cause death”.

Do you recall seeing or hearing anything about this aspect of the news story before now?

1: Yes

2: No

Not sure

[All who recall seeing or hearing anything about this news story before now]

Q.15 The estimate of the number of women who may have had their lives shortened that the Health Secretary gave was between 135 and 270. It was also reported that one statistics expert has said this claim is “misleading” because they believe “there is only weak evidence that screening helps prolong life, particularly for older women” and that “contrary to popular belief, screening also does harm...for every 200 women attending screening between 50 and 70, we would expect one to have her early death from breast cancer prevented, but three to be unnecessarily treated for a harmless cancer that would not have troubled them".

Do you recall seeing or hearing anything about this aspect of the news story before now?

1: Yes

2: No

Not sure

For peer review only

[All Adults 16+ in England willing to continue]

Q.16 In general, how much do you trust your general practitioner?

1: Not at all

2: A little

3: Somewhat

4: A lot

Not sure

[All Adults 16+ in England willing to continue]

Q.17 In general, how much do you trust the NHS?

1: Not at all

2: A little

3: Somewhat

4: A lot

Not sure

[All Adults 16+ in England willing to continue]

Q.18 Routine screening means testing healthy people to find cancer before they have any symptoms.

Do you think routine cancer screening tests for healthy people are almost always a good idea?

1: Yes

2: No

For peer review only

Not sure

[Participants were handed an information card with the following text and asked to read it]

You may have some questions about breast cancer screening after this part of the survey. You can find out more by calling the NHS on a Freephone number (0800 169 2692) or via the web on https:\\www.nhs.uk\conditions\breast-cancer-screening\missed-invitations\.

[All Adults 16+ in England willing to continue]

Q.19 It was also reported in May that a volcano had erupted in Hawaii, leading to officials declaring a state of emergency and mandatory evacuation of 1,700 residents in the area.

Do you recall seeing or hearing anything about this news story before now?

1: Yes

2: No

Not sure

[All Adults 16+ in England willing to continue]

Q.20 The results of local elections held in England were also reported in May. The Labour Party won 2,350 seats, the Conservative Party won 1,332 seats, and the Liberal Democrats won 536 seats.

Do you recall seeing or hearing anything about this news story before now?

1: Yes

2: No

Not sure

For peer review only

[All Adults 16+ in England willing to continue]

Q.21 Please can I ask you what is the highest level of qualification you have received?

1: A. Graduate level qualifications and above: including higher degrees, professional qualifications at HE standard (e.g. chartered accountant, surveyor, Nursing, Teaching), NVQ and SVQ Level 4 or 5, Higher Education Diplomas, HNC and HND and BTEC Higher, RSA Higher Diploma

2: B. A-levels and AS levels and equivalents: including SCE Higher, Scottish Certificate 6th Year Studies, NVQ and SVQ and GSVQ level 3, GNVQ Advanced, ONC and OND and BTEC National, City and Guilds Advanced Craft, City and Guilds Final level or Part III, RSA Advanced Diploma

3: C. Trade apprenticeships

4: D. GCSEs and equivalents: including O level, SCE Standard, CSEs, NVQ and SVQ and GSVQ level 1 and 2, GNVQ and BTEC and SCOTVEC first, General diploma, City and Guilds Ordinary level, City and Guilds Ordinary level Part II, RSA State I-III or Diploma, SCOTVEC modules

5: E. Other qualifications (including overseas)

6: F. No formal qualifications

Don’t know

Refused

For peer review only

APPENDIX 2 – SUPPLMENTARY TABLES

Table A – Summary statistics describing the sample

Measure	Total* (n=1,894)	%	(95% CI)
**Awareness of the news about breast screening**			
Aware of the main story and both follow-up commentaries	250	13.2	11.7 to 14.8
Aware of the main story and overdiagnosis follow-up	188	9.9	8.6 to 11.3
Aware of the main story and all-cause mortality follow-up	117	6.2	5.2 to 7.3
Aware of the main story only	709	37.4	35.3 to 39.6
Unaware of the story	630	33.3	31.2 to 35.4
**Recruitment wave**			
Wave 2: 20-26th June	606	32.0	29.9 to 34.1
Wave 1: 6-10th June	1,288	68.0	65.9 to 70.1
**Gender**			
Male	801	42.3	40.1 to 44.5
Female	1,093	57.7	55.5 to 59.9
**Ethnicity**			
White British	1,555	82.4	80.7 to 84.1
Other groups	331	17.6	15.9 to 19.3
**Marital status**			
Married or living as a married	1,039	54.9	52.6 to 57.1
Widowed, divorced and separated	382	20.2	18.4 to 22.0
Single	473	25.0	23.1 to 27.0
**Highest level of education**			
Graduate level qualifications and above	530	28.2	26.2 to 30.3
A-levels and AS levels and equivalents	461	24.6	22.7 to 26.5
GCSEs and equivalents	459	24.5	22.5 to 26.4
Trade apprenticeships or other qualifications	93	5.0	4.0 to 6.0
No formal qualifications	334	17.8	16.1 to 19.6
**Social class grade**			
Grade A or B	336	17.7	16.1 to 19.5
Grade C1	539	28.5	26.5 to 30.5
Grade C2	423	22.3	20.5 to 24.3
Grade D or E	596	31.5	29.4 to 33.6
**Employment status**			
Working	862	45.5	43.3 to 47.8
Not working	1,032	54.5	52.2 to 56.7
**Area type**			
Urban	1,542	81.4	79.6 to 83.1
Rural	352	18.6	16.9 to 20.4
**Personal diagnosis of cancer**			
Yes	156	8.4	7.2 to 9.7
No	1,705	91.6	90.3 to 92.8
**Personal experience of breast screening**			
Taken part	441	23.7	21.8 to 25.7
Invited, never taken part	56	3.0	2.3 to 3.9
Not eligible or not invited	1,364	73.3	71.2 to 75.3
**Belief that screening is almost always a good idea**			
Yes	1,737	91.7	90.4 to 92.9
No or not sure	157	8.3	7.1 to 9.6
**Awareness of the news about the volcanic eruption**			
Yes	1,435	75.8	73.8 to 77.7
No or not sure	459	24.2	22.3 to 26.2
**Awareness of the news about the local elections**			
Yes	1,198	63.3	61.1 to 65.4
No or not sure	696	36.7	34.6 to 38.9
**General level of trust in participants' GPs**			
A lot	1,009	55.2	52.9 to 57.5
Somewhat	540	29.6	27.5 to 31.7
A little	219	12.0	10.6 to 13.5
Not at all	59	3.2	2.5 to 4.1
**General level of trust in the NHS**			

For peer review only - http://bmjopen.bmj.com/site/about/guidelines.xhtml

A lot	1,016	54.2	54.9 to 59.5
Somewhat	619	33.0	32.7 to 37.1
A little	184	9.8	9.0 to 11.8
Not at all	56	3.0	2.4 to 4.0
**Frequency of worry about breast cancer**			
Very often	46	4.6	3.4 to 6.0
Often	55	5.4	4.2 to 7.0
Sometimes	216	21.4	18.9 to 24.0
Occasionally	302	29.9	27.1 to 32.8
Never	391	38.7	35.7 to 41.7
**Breast screening intentions for next invitation**			
Yes, definitely	690	84.7	82.1 to 87.0
Yes, probably	88	10.8	8.8 to 13.1
No, probably not	18	2.2	1.4 to 3.4
No, definitely not	19	2.3	1.5 to 3.5

Table B – Full results of the binary logistic regression model testing for variables associated with whether participants responded to questions on the survey module

		Responded vs. Did not respond to the survey questions: n (%)		Adjusted OR, 95% CI	p-value
**Characteristic**	**Total (n=2,665)**	**Did not respond (n=779; 29.2%)**	**Responded (n=1,886; 70.8%)**	**Responded to questions (vs. Did not respond)**	
**Recruitment wave**					
Wave 2: 20-26th June	908	303 (33.4)	605 (66.6)	0.73, 0.61 to 0.87	**<.0005**
vs. Wave 1: 6-10th June	1,757	476 (27.1)	1,281 (72.9)		
**Gender**					
Male	1,270	474 (37.3)	796 (62.7)	0.46, 0.39 to 0.55	**<.0005**
vs. Female	1,395	305 (21.9)	1,090 (78.1)		
**Ethnicity**					
White British	2,139	584 (27.3)	1,555 (72.7)	1.69, 1.37 to 2.10	**<.0005**
vs. Other groups	526	195 (37.1)	331 (62.9)		
**Marital status**					Overall: **.001**
Married/Living as a couple	1,441	407 (28.2)	1,034 (71.8)	1.48, 1.18 to 1.85	**.001**
Widowed/Divorced/Separated	517	135 (26.1)	382 (73.9)	1.65, 1.21 to 2.24	**.002**
vs. Single	707	237 (33.5)	470 (66.5)		
**Social class grade**					Overall: **.003**
Grade A or B	450	115 (25.6)	335 (74.4)	1.54, 1.18 to 2.02	**.002**
Grade C1	726	190 (26.2)	536 (73.8)	1.44, 1.15 to 1.81	**.002**
Grade C2	596	174 (29.2)	422 (70.8)	1.28, 1.01 to 1.63	**.045**
vs. Grade D or E	893	300 (33.6)	593 (66.4)		
**Employment status**					
Working	1,225	366 (29.9)	859 (70.1)	0.79, 0.65 to 0.97	**.026**
vs. Not working	1,440	413 (28.7)	1,027 71.3)		
**Area type**					
Urban	2,164	629 (29.1)	1,535 (70.9)	1.14, 0.91 to 1.42	.246
vs. Rural	501	150 (29.9)	351 (70.1)		
**Age (in years)**	2,665	52.1 (21.0)	50.8 (20.5)	0.99, 0.98 to 1.00	**<.0005**

Table C – Sources of news about the breast screening story

Source of information	Total (n=1,264)	%	(95% CI)
Television	971	76.8	74.4 to 79.1
Radio	271	21.4	19.2 to 23.8
Print newspaper(s)	169	13.4	11.6 to 15.3
Online news websites	134	10.6	9.0 to 12.4
Social media websites	68	5.4	4.2 to 6.7
Other websites	11	0.9	0.5 to 1.5

For peer review only - http://bmjopen.bmj.com/site/about/guidelines.xhtml

Word of mouth	43	3.4	2.5 to 4.5
Other sources	8	0.6	0.3 to 1.2
Discussed or shared the story with someone else	450	35.6	33.0 to 38.3

Table D – Full results of the binary logistic regression model testing for an association between awareness of the breast screening media coverage and trust in participants' GPs

		A lot vs. Not at all; a little; somewhat: n (%)		Adjusted OR, 95% CI	p-value
**Characteristic**	**Total (n=1,746)**	**Less than a lot (n=781; 44.7%)**	**A lot (n=965; 55.3%)**	**A lot (vs. Less than a lot)**	
**Screening story awareness**					Overall: .729
Aware of the main story and both follow-up commentaries	238	98 (41.2)	140 (58.8)	1.10, 0.74 to 1.64	.653
Aware of the main story and overdiagnosis follow-up	172	66 (38.4)	106 (61.6)	1.31, 0.85 to 2.03	.218
Aware of the main story and all-cause mortality follow-up	107	49 (45.8)	58 (54.2)	1.21, 0.73 to 2.02	.459
Aware of the main story only	655	280 (42.7)	375 (57.3)	1.17, 0.88 to 1.57	.283
vs. Unaware of the story	574	288 (50.2)	286 (49.8)		
**Recruitment wave**					
Wave 2: 20-26th June	557	255 (45.8)	663 (55.8)	0.81, 0.64 to 1.04	.097
vs. Wave 1: 6-10th June	1,189	526 (44.2)	302 (54.2)		
**Gender**					
Male	754	317 (42.0)	437 (58.0)	1.15, 0.86 to 1.54	.334
vs. Female	992	464 (46.8)	528 (53.2)		
**Ethnicity**					
White British	1,450	614 (42.3)	836 (57.7)	1.17, 0.85 to 1.61	.328
vs. Other groups	296	167 (54.4)	129 (43.6)		
**Marital status**					Overall: .504
Married/Living as a couple	964	422 (42.8)	542 (56.2)	1.04, 0.77 to 1.42	.782
Widowed/Divorced/Separated	341	139 (40.8)	202 (59.2)	0.86, 0.57 to 1.29	.460
vs. Single	441	220 (49.9)	221 (50.1)		
**Highest level of education**					Overall: .056
Graduate level/Above	494	230 (46.6)	264 (53.4)	0.64, 0.42 to 0.98	.042
A-levels/AS levels/Equivalents	438	201 (45.9)	237 (54.1)	0.70, 0.46 to 1.06	.089
GCSEs/Equivalents	429	213 (49.7)	216 (50.3)	0.63, 0.43 to 0.94	.022
Trade apprenticeships/Other	86	41 (47.7)	45 (52.3)	0.44, 0.25 to 0.80	.007
vs. No formal qualifications	299	96 (32.1)	203 (67.9)		
**Social class grade**					Overall: .711
Grade A or B	317	128 (40.4)	189 (59.6)	1.20, 0.82 to 1.76	.342
Grade C1	505	231 (45.7)	274 (54.3)	1.02, 0.74 to 1.39	.923
Grade C2	385	181 (47.0)	204 (53.0)	0.97, 0.70 to 1.36	.874
vs. Grade D or E	539	241 (44.7)	298 (55.3)		
**Employment status**					
Working	806	411 (51.0)	395 (49.0)	0.82, 0.63 to 1.07	.135
vs. Not working	940	370 (39.4)	570 (60.6)		
**Area type**					
Urban	1,420	635 (44.7)	785 (55.3)	1.12, 0.84 to 1.50	.430
vs. Rural	326	146 (44.8)	180 (55.2)		
**Personal diagnosis of cancer**					
Yes	1,599	718 (44.9)	881 (55.1)	0.84, 055 to 1.28	.404
vs. No	147	63 (42.9)	84 (57.1)		
**Personal experience of breast screening**					Overall: .284
Taken part	411	159 (38.7)	252 (61.3)	1.33, 0.91 to 1.95	.145
Invited, never taken part	48	21 (43.8)	27 (56.3)	0.95, 0.46 to 1.98	.894
vs. Not eligible or not invited	1,287	601 (46.7)	686 (53.3)		

For peer review only

**Belief that screening is almost always a good idea**					
Yes	1,609	701 (43.6)	908 (56.4)	1.30, 0.85 to 1.97	.230
vs. No or not sure	137	80 (58.4)	57 (41.6)		
**Awareness of volcano news**					
Yes	1,332	565 (42.4)	767 (57.6)	1.04, 0.77 to 1.40	.789
vs. No or not sure	414	216 (52.2)	198 (47.8)		
**Awareness of election news**					
Yes	1,114	467 (41.9)	647 (58.1)	1.19, 0.93 to 1.54	.172
vs. No or not sure	632	314 (49.7)	318 (50.3)		
**General level of trust in the NHS**					Overall: **<.0005**
A lot	943	202 (21.4	741 (78.6)	13.53, 6.65 to 27.54	**<.0005**
Somewhat	589	409 (69.4)	180 (30.6)	1.62, 0.80 to 3.31	.183
A little	163	130 (79.8)	33 (20.2)	1.00, 0.46 to 2.21	.994
vs. Not at all	51	40 (78.4)	11 (21.6)		
**Age (in years)**	1,746	46.9 (19.1)	53.9 (21.0)	1.01, 1.00 to 1.02	.087

Table E – Full results of the binary logistic regression model testing for an association between awareness of the breast screening media coverage and trust in the NHS

		**A lot vs. Not at all; a little; somewhat: n (%)**		**Adjusted OR, 95% CI**	**p-value**
**Characteristic**	**Total (n=1,746)**	**Less than a lot (n=803; 46.0%)**	**A lot (n=943; 54.0%)**	**A lot (vs. Less than a lot)**	
**Screening story awareness**					Overall: .290
Aware of the main story and both follow-up commentaries	238	102 (42.9)	136 (57.1)	0.87, 0.59 to 1.30	.503
Aware of the main story and overdiagnosis follow-up	172	76 (44.2)	96 (55.8)	0.78, 0.51 to 1.21	.267
Aware of the main story and all-cause mortality follow-up	107	57 (53.3)	50 (46.7)	0.58, 0.35 to 0.97	.039
Aware of the main story only	655	299 (45.6)	356 (54.4)	0.81, 0.60 to 1.09	.160
vs. Unaware of the story	574	269 (46.9)	305 (53.1)		
**Recruitment wave**					
Wave 2: 20-26th June	557	248 (44.5)	309 (55.5)	1.21, 0.95 to 1.55	.118
vs. Wave 1: 6-10th June	1,189	555 (46.7)	634 (53.3)		
**Age**					Overall: .052
65+	530	198 (37.4)	332 (62.6)	1.04, 0.61 to 1.79	.880
55-64	245	111 (45.3)	134 (54.7)	0.76, 0.44 to 1.30	.309
45-54	235	115 (48.9)	120 (51.1)	0.83, 0.50 to 1.39	.484
35-44	245	135 (55.1)	110 (44.9)	0.59, 0.36 to 0.96	.035
25-34	265	152 (57.4)	113 (42.6)	0.56, 0.35 to 0.91	.018
vs. 16-24	226	92 (40.7)	134 (59.3)		
**Gender**					
Male	754	319 (42.3)	435 (57.7)	0.99, 0.73 to 1.33	.985
vs. Female	992	484 (48.8)	508 (51.2)		
**Ethnicity**					
White British	1,450	634 (43.7)	816 (56.3)	1.47, 1.07 to 2.02	**.019**
vs. Other groups	296	169 (57.1)	127 (42.9)		
**Marital status**					Overall: .870
Married/Living as a couple	964	440 (45.6)	524 (54.4)	1.07, 0.78 to 1.47	.685
Widowed/Divorced/Separated	341	151 (44.3)	190 (55.7)	1.00, 0.66 to 1.50	.990
vs. Single	441	212 (48.1)	229 (51.9)		
**Highest level of education**					Overall: .076
Graduate level/Above	494	233 (47.2)	261 (52.8)	1.20, 0.79 to 1.83	.386
A-levels/AS levels/Equivalents	438	199 (45.4)	239 (54.6)	1.12, 0.75 to 1.67	.582
GCSEs/Equivalents	429	224 (52.2)	205 (47.8)	0.86, 0.59 to 1.26	.447
Trade apprenticeships/Other	86	32 (37.2)	54 (62.8)	1.85, 1.01 to 3.39	.047
vs. No formal qualifications	299	115 (38.5)	184 (61.5)		

For peer review only

**Social class grade**					Overall: .990
Grade A or B	317	140 (44.2)	177 (55.8)	0.96, 0.65 to 1.41	.828
Grade C1	505	232 (45.9)	273 (54.1)	0.99, 0.72 to 1.37	.968
Grade C2	385	179 (46.5)	206 (53.5)	1.02, 0.73 to 1.43	.892
vs. Grade D or E	539	252 (46.8)	206 (53.5)		
**Employment status**					
Working	806	410 (50.9)	396 (49.1)	0.94, 0.71 to 1.25	.673
vs. Not working	940	393 (41.8)	547 (58.2)		
**Area type**					
Urban	1,420	656 (46.2)	764 (53.8)	0.96, 0.72 to 1.29	.795
vs. Rural	326	147 (45.1)	179 (54.9)		
**Personal diagnosis of cancer**					
Yes	1,599	737 (46.1)	862 (53.9)	1.00, 0.66 to 1.50	.994
vs. No	147	66 (44.9)	81 (55.1)		
**Personal experience of breast screening**					Overall: .062
Taken part	411	193 (47.0)	218 (53.0)	0.65, 0.43 to 0.97	.035
Invited, never taken part	48	20 (41.7)	28 (58.3)	1.10, 0.51 to 2.35	.813
vs. Not eligible or not invited	1,287	590 (45.8)	697 (54.2)		
**Belief that screening is almost always a good idea**					
Yes	1,609	715 (44.4)	894 (55.6)	1.96, 1.28 to 3.00	**.002**
vs. No or not sure	137	88 (64.2)	49 (35.8)		
**Awareness of volcano news**					
Yes	1,332	594 (44.6)	738 (55.4)	1.00, 0.74 to 1.35	.987
vs. No or not sure	414	209 (50.5)	205 (49.5)		
**Awareness of election news**					
Yes	1,114	491 (44.1)	623 (55.9)	1.03, 0.80 1.33	.817
vs. No or not sure	632	312 (49.4)	320 (50.6)		
**General level of trust in participants' GPs**					Overall: **<.0005**
A lot	965	224 (23.2)	741 (76.8)	11.98, 6.07 to 23.64	**<.0005**
Somewhat	513	370 (72.1)	143 (27.9)	1.39, 0.70 to 2.76	.350
A little	212	165 (77.8)	47 (22.2)	1.12, 0.54 to 2.33	.770
vs. Not at all	56	44 (78.6)	12 (21.4)		

Table F – Full results of the binary logistic regression model testing for an association between awareness of the breast screening media coverage and frequency of worry about breast cancer

		**Never; occasionally vs. Sometimes; often; very often: n (%)/M (SD)**		**Adjusted OR, 95% CI**	**p-value**
**Characteristic**	**Total (n=700)**	**Never; occasionally (n=441; 63.0%)**	**Sometimes; often; very often (n=259; 37.0%)**	**Sometimes; often; very often (vs. Never; occasionally)**	
**Screening story awareness**					Overall: .198
Aware of the main story and both follow-up commentaries	88	65 (73.9)	23 (26.1)	0.85, 0.46 to 1.58	.614
Aware of the main story and overdiagnosis follow-up	63	42 (66.7)	21 (33.3)	1.05, 0.55 to 2.01	.878
Aware of the main story and all-cause mortality follow-up	36	25 (69.4)	11 (30.6)	1.10, 0.49 to 2.49	.819
Aware of the main story only	270	153 (56.7)	117 (43.3)	1.49, 0.98 to 2.25	.062
vs. Unaware of the story	243	156 (64.2)	87 (35.8)		
**Recruitment wave**					
Wave 2: 20-26th June	229	152 (66.4)	77 (33.6)	0.83, 0.58 to 1.19	.304
vs. Wave 1: 6-10th June	471	289 (61.4)	182 (38.6)		
**Ethnicity**					
White British	563	354 (62.9)	209 (37.1)	0.90, 0.58 to 1.39	.635
vs. Other groups	137	87 (63.5)	50 (36.5)		
**Marital status**					Overall: .272

For peer review only - http://bmjopen.bmj.com/site/about/guidelines.xhtml

Married/Living as a couple	403	255 (63.3)	148 (36.7)	1.14, 0.76 to 1.72	.519
Widowed/Divorced/Separated	100	58 (58.0)	42 (42.0)	1.61, 0.90 to 2.87	.110
vs. Single	197	128 (65.0)	69 (35.0)		
**Highest level of education**					Overall: **.026**
Graduate level/Above	230	149 (64.8)	81 (35.2)	0.40, 0.21 to 0.79	**.008**
A-levels/AS levels/Equivalents	206	138 (67.0)	68 (33.0)	0.67, 0.19 to 0.70	**.002**
GCSEs/Equivalents	176	105 (59.7)	71 (40.3)	0.57, 0.31 to 1.04	.068
Trade apprenticeships/Other	17	12 (70.6)	5 (29.4)	0.33, 0.10 to 1.10	.072
vs. No formal qualifications	71	37 (52.1)	34 (47.9)		
**Social class grade**					Overall: .704
Grade A or B	125	84 (67.2)	41 (32.8)	1.07, 0.60 to 1.90	.819
Grade C1	209	123 (58.9)	86 (41.1)	1.31, 0.80 to 2.13	.282
Grade C2	165	105 (63.6)	60 (36.4)	1.11, 0.67 to 1.83	.691
vs. Grade D or E	201	129 (64.2)	72 (35.8)		
**Employment status**					
Working	392	239 (61.0)	153 (39.0)	1.15, 0.81 to 1.64	.435
vs. Not working	308	202 (65.6)	106 (34.4)		
**Area type**					
Urban	574	366 (63.8)	208 (36.2)	0.83, 054 to 1.26	.378
vs. Rural	126	75 (59.5)	51 (40.5)		
**Personal diagnosis of cancer**					
Yes	44	26 (59.1)	18 (40.9)	1.62, 0.82 to 3.22	.169
vs. No	656	415 (63.3)	241 (36.7)		
**Personal experience of breast screening**					Overall: .428
Taken part	221	151 (68.3)	70 (31.7)	0.66, 0.36 to 1.23	.193
Invited, never taken part	34	23 (67.6)	11 (32.4)	0.75, 0.30 to 1.89	.537
vs. Not eligible or not invited	445	267 (60.0)	178 (40.0)		
**Belief that screening is almost always a good idea**					
Yes	660	412 (62.4)	248 (37.6)	1.12, 0.50 to 2.51	.779
vs. No or not sure	40	29 (72.5)	11 (27.5)		
**Awareness of volcano news**					
Yes	505	323 (64.0)	182 (36.0)	0.91, 0.61 to 1.36	.645
vs. No or not sure	195	118 (60.5)	77 (39.5)		
**Awareness of election news**					
Yes	424	275 (64.9)	149 (35.1)	0.84, 0.59 to 1.20	.340
vs. No or not sure	276	166 (60.1)	110 (39.9)		
**General level of trust in participants' GPs**					Overall: .791
A lot	339	220 (64.9)	119 (35.1)	1.26, 0.50 to 3.15	.626
Somewhat	221	131 (59.3)	90 (40.7)	1.47, 0.58 to 3.72	.412
A little	109	69 (63.3)	40 (36.7)	1.25, 0.49 to 3.22	.641
vs. Not at all	31	21 (67.7)	10 (32.3)		
**General level of trust in the NHS**					Overall: .744
A lot	344	223 (64.8)	121 (35.2)	1.14, 0.41 to 3.21	.802
Somewhat	257	151 (58.8)	106 (41.2)	1.39, 0.49 to 3.91	.535
A little	76	51 (67.1)	25 (32.9)	1.13, 0.38 to 3.36	.833
vs. Not at all	23	16 (69.6)	7 (30.4)		
**Breast screening intentions for next invitation**					Overall: .163
Yes, definitely	601	369 (61.4)	232 (38.6)	1.48, 0.47 to 4.68	.503
Yes, probably	70	49 (70.0)	21 (30.0)	0.93, 0.27 to 3.25	.913
No, probably not	13	12 (92.3)	1 (7.7)	0.25, 0.02 to 2.70	.254
vs. No, definitely not	16	11 (68.8)	5 (31.3)		
**Age (in years)**	700	43.7 (15.5)	41.8 (14.3)	0.99, 0.97 to 1.01	.463

Table G – Full results of the binary logistic regression model testing for an association between awareness of the breast screening media coverage and breast screening intentions

For peer review only

For peer review only - http://bmjopen.bmj.com/site/about/guidelines.xhtml

		Yes, definitely vs. Yes, probably; no, probably not; no, definitely not: n (%)		Adjusted OR, 95% CI	p-value
**Characteristic**	**Total (n=700)**	**No definite intention (n=99; 14.1%)**	**Definite intention (n=601; 85.9%)**	**Definite intention (vs. No definite intention)**	
**Screening story awareness**					Overall: .108
Aware of the main story and both follow-up commentaries	88	10 (11.4)	78 (88.6)	2.01, 0.74 to 5.48	.172
Aware of the main story and overdiagnosis follow-up	63	4 (4.3)	59 (93.7)	2.66, 0.79 to 8.89	.113
Aware of the main story and all-cause mortality follow-up	36	6 (16.7)	30 (83.3)	0.66, 0.20 to 2.13	.486
Aware of the main story only	270	22 (8.1)	248 (91.9)	1.88, 0.99 to 3.57	.054
vs. Unaware of the story	243	57 (23.5)	186 (76.5)		
**Recruitment wave**					
Wave 2: 20-26th June	229	40 (17.5)	189 (82.5)	0.71, 0.42 to 1.21	.211
vs. Wave 1: 6-10th June	471	59 (12.5)	412 (87.5)		
**Age**					Overall: .050
65+	69	7 (10.1)	62 (89.9)	0.84, 0.13 to 5.37	.855
55-64	123	5 (4.1)	118 (95.9)	2.49, 0.42 to 14.74	.313
45-54	128	10 (7.8)	118 (92.2)	3.59, 1.10 to 11.69	.034
35-44	143	21 (14.7)	122 (85.3)	2.72, 1.16 to 6.41	.022
25-34	144	29 (20.1)	115 (79.9)	2.82, 1.24 to 6.42	.014
vs. 16-24	93	27 (29.0)	66 (71.0)		
**Ethnicity**					
White British	563	72 (12.8)	491 (87.2)	0.96, 0.51 to 1.83	.905
vs. Other groups	137	27 (19.7)	110 (80.3)		
**Marital status**					Overall: .321
Married/Living as a couple	403	49 (12.2)	354 (87.8)	0.99, 0.53 to 1.84	.970
Widowed/Divorced/Separated	100	6 (6.0)	94 (94.0)	2.18, 0.73 to 6.53	.163
vs. Single	197	44 (22.3)	153 (77.7)		
**Highest level of education**					Overall: .169
Graduate level/Above	230	35 (15.2)	195 (84.8)	0.69, 0.22 to 2.20	.533
A-levels/AS levels/Equivalents	206	35 (17.0)	171 (83.0)	0.87, 0.29 to 2.62	.808
GCSEs/Equivalents	176	19 (10.8)	157 (89.2)	1.92, 0.62 to 5.92	.259
Trade apprenticeships/Other	17	3 (17.6)	14 (82.4)	1.38, 0.24 to 0.81	.720
vs. No formal qualifications	71	7 (9.9)	64 (90.1)		
**Social class grade**					Overall: .186
Grade A or B	125	14 (11.2)	111 (88.8)	1.87, 0.76 to 4.61	.177
Grade C1	209	25 (12.0)	184 (88.0)	2.36, 1.08 to 5.16	.032
Grade C2	165	24 (14.5)	141 (85.5)	1.85, 0.86 to 3.98	.118
vs. Grade D or E	201	36 (17.9)	165 (82.1)		
**Employment status**					
Working	392	56 (14.3)	336 (85.7)	0.70, 0.39 to 1.26	.238
vs. Not working	308	43 (14.0)	265 (86.0)		
**Area type**					
Urban	574	83 (14.5)	491 (85.5)	0.79, 0.39 to 1.59	.512
vs. Rural	126	16 (12.7)	110 (87.3)		
**Personal diagnosis of cancer**					
Yes	44	7 (15.9)	37 (84.1)	0.94, 0.30 to 2.98	.918
vs. No	656	92 (14.0)	564 (86.0)		
**Personal experience of breast screening**					Overall: **.013**
Taken part	221	9 (4.1)	212 (95.9)	6.12, 1.37 to 27.33	**.018**
Invited, never taken part	34	6 (17.6)	28 (82.4)	0.99, 0.21 to 4.61	.986
vs. Not eligible or not invited	445	84 (18.9)	361 (81.1)		
**Belief that screening is almost always a good idea**					
Yes	660	81 (12.3)	579 (87.7)	9.08, 3.77 to 21.88	**<.0005**
vs. No or not sure	40	18 (45.0)	22 (55.0)		
**Awareness of volcano news**					
Yes	505	54 (10.7)	451 (89.3)	1.34, 0.74 to 2.41	.335
vs. No or not sure	195	45 (23.1)	150 (76.9)		

For peer review only

For peer review only - http://bmjopen.bmj.com/site/about/guidelines.xhtml

**Awareness of election news**					
Yes	424	47 (11.1)	377 (88.9)	1.42, 0.82 to 2.46	.208
vs. No or not sure	276	52 (18.8)	224 (81.2)		
**General level of trust in participants' GPs**					Overall: .025
A lot	339	31 (9.1)	308 (90.9)	1.55, 0.45 to 5.29	.487
Somewhat	221	44 (19.9)	177 (80.1)	0.76, 0.23 to 5.67	.663
A little	109	16 (14.7)	93 (85.3)	2.48, 0.69 to 8.90	.163
vs. Not at all	31	8 (25.8)	23 (74.2)		
**General level of trust in the NHS**					Overall: .007
A lot	344	34 (9.9)	310 (90.1)	1.16, 0.29 to 4.64	.832
Somewhat	257	42 (16.3)	215 (83.7)	0.70, 0.18 to 2.79	.614
A little	76	18 (23.7)	58 (76.3)	0.27, 0.06 to 1.11	.068
vs. Not at all	23	5 (21.7)	18 (78.3)		
**Frequency of worry about breast cancer**					Overall: **.028**
Very often	39	3 (7.7)	36 (92.3)	3.00, 0.72 to 12.51	.132
Often	43	5 (9.3)	39 (90.7)	2.95, 0.85 to 10.26	.089
Sometimes	177	20 (11.3)	157 (88.7)	2.59, 1.31 to 5.15	**.006**
Occasionally	231	27 (11.7)	204 (88.3)	2.15, 1.15 to 4.02	**.016**
vs. Never	210	45 (21.4)	165 (78.6)		

Table H – Number of participants with missing data for each variable of interest

**Measure**	**Total (n=1,894)**
Awareness of the news about breast screening	0
Recruitment wave	0
Gender	0
Ethnicity	8
Marital status	0
Highest level of education	17
Social class grade	0
Employment status	0
Area type	0
Personal diagnosis of cancer	33
Personal experience of breast screening	33
Belief that screening is almost always a good idea	**0**
Awareness of the news about the volcanic eruption	0
Awareness of the news about the local elections	0
General level of trust in participants' GPs	67
General level of trust in the NHS	19
Frequency of worry about breast cancer	22
Breast screening intentions for next invitation	9

For peer review only - http://bmjopen.bmj.com/site/about/guidelines.xhtml

STROBE Statement—Checklist of items that should be included in reports of ***cross-sectional studies***

	Item No	Recommendation	Page number
**Title and abstract**	1	(*a*) Indicate the study's design with a commonly used term in the title or the abstract	1
		(*b*) Provide in the abstract an informative and balanced summary of what was done and what was found	2
**Introduction**			
Background/rationale	2	Explain the scientific background and rationale for the investigation being reported	3-4
Objectives	3	State specific objectives, including any prespecified hypotheses	4
**Methods**			
Study design	4	Present key elements of study design early in the paper	4
Setting	5	Describe the setting, locations, and relevant dates, including periods of recruitment, exposure, follow-up, and data collection	4
Participants	6	(*a*) Give the eligibility criteria, and the sources and methods of selection of participants	4-5
Variables	7	Clearly define all outcomes, exposures, predictors, potential confounders, and effect modifiers. Give diagnostic criteria, if applicable	5-7
Data sources/ measurement	8*	For each variable of interest, give sources of data and details of methods of assessment (measurement). Describe comparability of assessment methods if there is more than one group	5-7
Bias	9	Describe any efforts to address potential sources of bias	4-8
Study size	10	Explain how the study size was arrived at	5
Quantitative variables	11	Explain how quantitative variables were handled in the analyses. If applicable, describe which groupings were chosen and why	7-8
Statistical methods	12	(*a*) Describe all statistical methods, including those used to control for confounding	7-8
		(*b*) Describe any methods used to examine subgroups and interactions	7-8
		(*c*) Explain how missing data were addressed	8
		(*d*) If applicable, describe analytical methods taking account of sampling strategy	N/A
		(*e*) Describe any sensitivity analyses	N/A
**Results**			
Participants	13*	(a) Report numbers of individuals at each stage of study—eg numbers potentially eligible, examined for eligibility, confirmed eligible, included in the study, completing follow-up, and analysed	8-13
		(b) Give reasons for non-participation at each stage	8
		(c) Consider use of a flow diagram	N/A
Descriptive data	14*	(a) Give characteristics of study participants (eg demographic, clinical, social) and information on exposures and potential confounders	8-9
		(b) Indicate number of participants with missing data for each variable of interest	P8 (appendix 2)
Outcome data	15*	Report numbers of outcome events or summary measures	8-13
Main results	16	(*a*) Give unadjusted estimates and, if applicable, confounder-adjusted estimates and their precision (eg, 95% confidence interval). Make clear which confounders were adjusted for and why they were included	11-13

For peer review only - http://bmjopen.bmj.com/site/about/guidelines.xhtml

		(*b*) Report category boundaries when continuous variables were categorized	11-13
		(*c*) If relevant, consider translating estimates of relative risk into absolute risk for a meaningful time period	N/A
Other analyses	17	Report other analyses done—eg analyses of subgroups and interactions, and sensitivity analyses	N/A
**Discussion**			
Key results	18	Summarise key results with reference to study objectives	8-10
Limitations	19	Discuss limitations of the study, taking into account sources of potential bias or imprecision. Discuss both direction and magnitude of any potential bias	15-16
Interpretation	20	Give a cautious overall interpretation of results considering objectives, limitations, multiplicity of analyses, results from similar studies, and other relevant evidence	16
Generalisability	21	Discuss the generalisability (external validity) of the study results	14-16
**Other information**			
Funding	22	Give the source of funding and the role of the funders for the present study and, if applicable, for the original study on which the present article is based	16

*Give information separately for exposed and unexposed groups.

Note: An Explanation and Elaboration article discusses each checklist item and gives methodological background and published examples of transparent reporting. The STROBE checklist is best used in conjunction with this article (freely available on the Web sites of PLoS Medicine at http://www.plosmedicine.org/, Annals of Internal Medicine at http://www.annals.org/, and Epidemiology at http://www.epidem.com/). Information on the STROBE Initiative is available at www.strobe-statement.org.

For peer review only
